# Uncovering the Potential Pan Proteomes Encoded by Genomic Strand RNAs of Influenza A Viruses

**DOI:** 10.1371/journal.pone.0146936

**Published:** 2016-01-13

**Authors:** Chu-Wen Yang, Mei-Fang Chen

**Affiliations:** 1 Department of Microbiology, Soochow University, Shih-Lin, Taipei 111, Taiwan, R.O.C.; 2 Department of Medical Research, Taipei Veterans General Hospital, Taipei 112, Taiwan, R.O.C.; Deakin University, AUSTRALIA

## Abstract

Influenza A virus genomes are composed of eight negative sense RNAs. In total, 16 proteins encoded by eight positive sense RNAs were identified. One putative protein coding sequence (PCS) encoded by genomic strand RNA of segment 8 has been previously proposed. In this study, 95,608, 123,965 and 35,699 genomic strand RNA sequences from influenza A viruses from avian, human and mammalian hosts, respectively, were used to identify PCSs encoded by the genomic strand RNAs. In total, 326,069 PCSs with lengths equal to or longer than 80 amino acids were identified and clustered into 270 PCS groups. Twenty of the 270 PCS groups which have greater than 10% proportion in influenza A viruses from avian, human or mammalian hosts were selected for detailed study. Maps of the 20 PCSGs in the influenza A virus genomes were constructed. The proportions of the 20 PCSGs in influenza A viruses from different hosts and serotypes were analyzed. One secretory and five membrane proteins predicted from the PCS groups encoded by genomic strand RNAs of segments 1, 2, 4, 6, 7 and 8 were identified. These results suggest the possibility of the ambisense nature of the influenza A virus genomic RNAs and a potential coding sequence reservoir encoding potential pan proteomes of influenza A viruses.

## Introduction

Influenza A virus (IAV) genomes are composed of eight negative (genomic) sense RNAs [1,2]. Currently, 16 proteins encoded by eight positive sense RNAs have been identified. Three proteins (PB1, PB1-F2 and N40) encoded by the positive sense RNA of segment 2 start at the 1st, 4th and 5th AUG, respectively [3,4,5]. Four proteins (PA, PA-X, PA-N155 and PA-N182) are encoded by the positive sense RNA of segment 3. The PA-X protein is a ribosomal frame-shifting product composed of the N-terminal domain of the PA protein (191 amino acids) and a short C-terminal domain (61 amino acids) that results from a +1 frameshift of the PA open reading frame (ORF) [6,7]. The PA-N155 and PA-N182 proteins are translated from the 11th and 13th in-frame AUGs in the PA ORF and are, therefore, N-terminally truncated forms of PA protein [8]. Three proteins (M1, M2 and M42) are encoded by the positive sense RNA of segment 7. An alternatively spliced mRNA encodes an M2 variant, called M42, which functionally complements M2 *in vitro* and *in vivo* [9]. The genomic map of these ORFs is summarized in S1 Fig. In addition to the proteins encoded by eight positive sense RNAs, a hypothetical protein sequence encoded by the genomic strand RNA of segment 8 was proposed [10,11,12]. These studies raise the possibility regarding the coding potential of the eight genomic strand RNAs of IAVs.

In this study, a large-scale *in silico* investigation was performed using the IAV genome sequences from the NCBI Influenza Database to identify putative protein coding sequences (PCSs) in the genomic strand RNAs of IAVs. In total, 270 PCS groups (PCSGs) composed of 326,069 PCSs with lengths equal to or longer than 80 amino acids were identified. Twenty of the 270 PCS groups with greater than 10% proportions in IAVs from avian, human or mammalian hosts (AIAV, HIAV and MIAV) were selected for further study.

## Materials and Methods

### Data Collection

In total, 322,235 IAV genome sequences were retrieved from the NCBI Influenza Database. After checking completeness by length and integrity of open reading frame in the positive strand, 255,273 IAV genomic strand RNA sequences were used. This data set includes 95,608, 123,965 and 35,699 genomic strand RNA sequences of IAVs from avian, human and mammalian hosts (AIAVs, HIAVs and MIAVs), respectively (S1 Table). A set of genomic strand RNA sequences from pandemic, outbreak or highly virulent HIAVs, including H1N1 1918 HIAV from the "Spanish Flu", H2N2 HIAV from the 1957 pandemic, H3N2 HIAV from the 1968 pandemic, H1N1 HIAV from the 1977 Russia outbreak, 2009 H1N1 HIAV from the “swine flu”, H5N1 HIAV from the 1997 Hong Kong outbreak, the 2004–2008 highly pathogenic H5N1 HIAVs from Vietnam, Indonesia and Thailand and the highly pathogenic H7N9 HIAV from China 2013 were used as models of special strains.

### PCS Prediction, Sequence Clustering and Phylogenetic Analysis

The computer programs for data manipulation and putative protein coding sequence (PCS) prediction were written by the authors using the Perl programming language. In total, 326,069 PCSs with a length equal to or longer than 80 amino acids were identified from 255,273 IAV genomic strand RNAs. The PCSs predicted from each genome segment were clustered using the CD-HIT software [13]. After sequence clustering, the CD-HIT software chose a longest sequence as a representative sequence for each sequence cluster and computed the identities between the representative sequence and the other sequences within the sequence group. Protein sequence identity of 60% was used as the cutoff of the first run of sequence clustering because the protein sequences from these PCSs exhibit both sequence and length variations. After first run clustering, 270 PCS groups (PCSGs) were obtained. Twenty of the 270 PCSGs with greater than 10% proportion in at least one of AIAVs, HIAVs or MIAVs were chosen for further study. Each of the 20 PCSGs was divided by IAV types (AIAV, HIAV and MIAV) and serotypes (H1N1, H2N2, H3N2, H5N1, H7N9 and other). Each of the 20 PCSGs was used to perform the second run of sequence clustering based on 95% identity. Representative protein sequences of sub-clusters from each of the 20 PCSGs were used to perform phylogenetic analysis using the MEGA6 software [14]. Phylogenetic trees of the 20 PCSGs were built by the NJ method with bootstrap 1000 times. One-way ANOVA analyses of the sequence identity and length variations of 20 PCSGs among the AIAVs, HIAVs and MIAVs were performed using EXCEL.

### Protein Function Domain Prediction

The Simple Modular Architecture Research Tool (SMART) [15] and NCBI CD-Search [16] were used for protein function domain identification. The TMHMM Server v. 2.0 [17] was used to confirm the prediction of the trans-membrane domains and signal sequences.

## Results

### PCS groups identified in the genomic strands of IAV RNAs *in silico*

**Fig 1:**
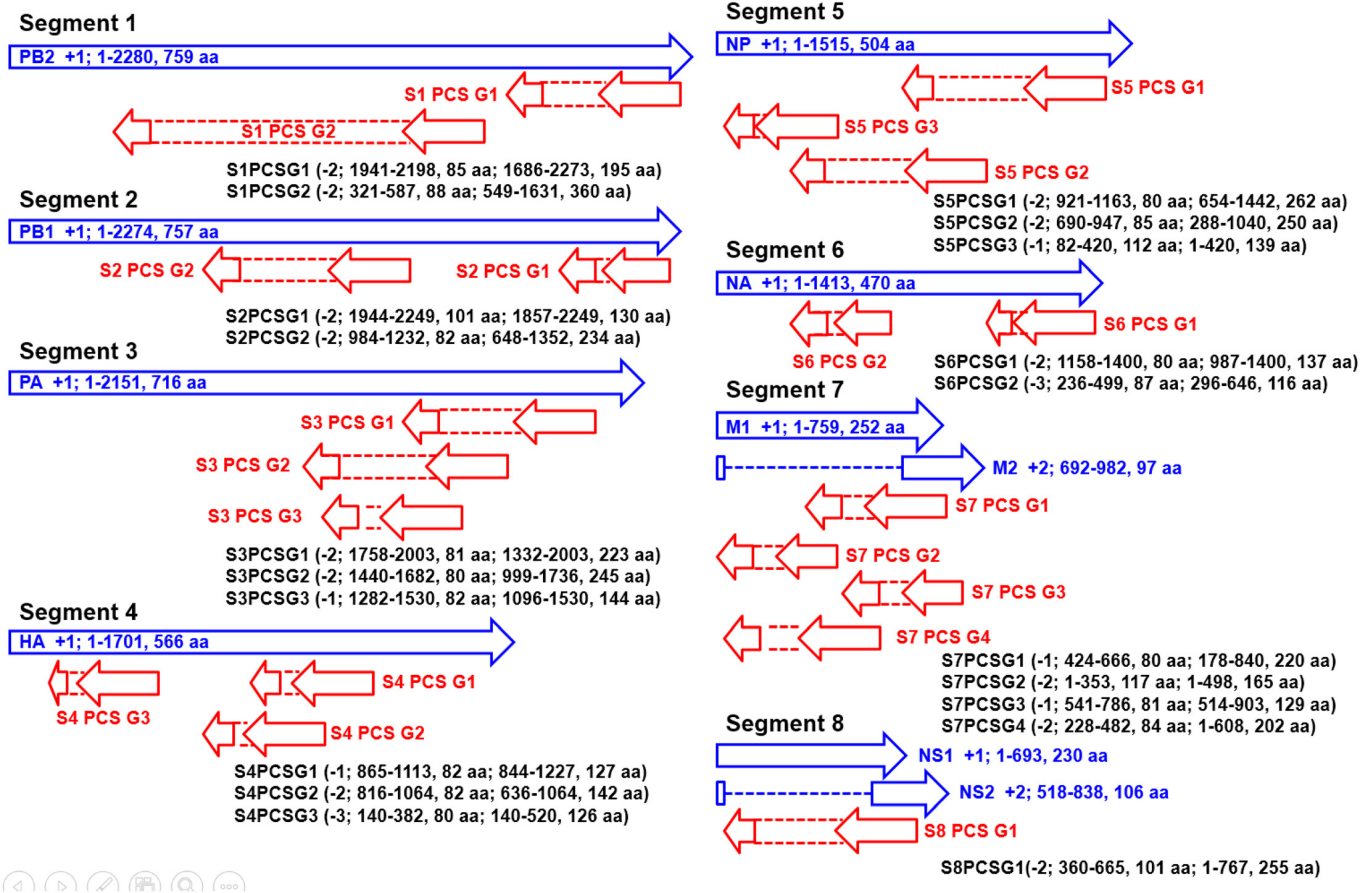
A genomic map of 20 putative protein coding sequence groups encoded by eight genomic strand RNAs of influenza A viruses identified in this study. Blue arrows indicate open reading frames encoded by eight positive sense RNAs of influenza A viruses. Red arrows indicate putative protein coding sequences encoded by eight genomic strand RNAs of influenza A viruses.

**Fig 2:**
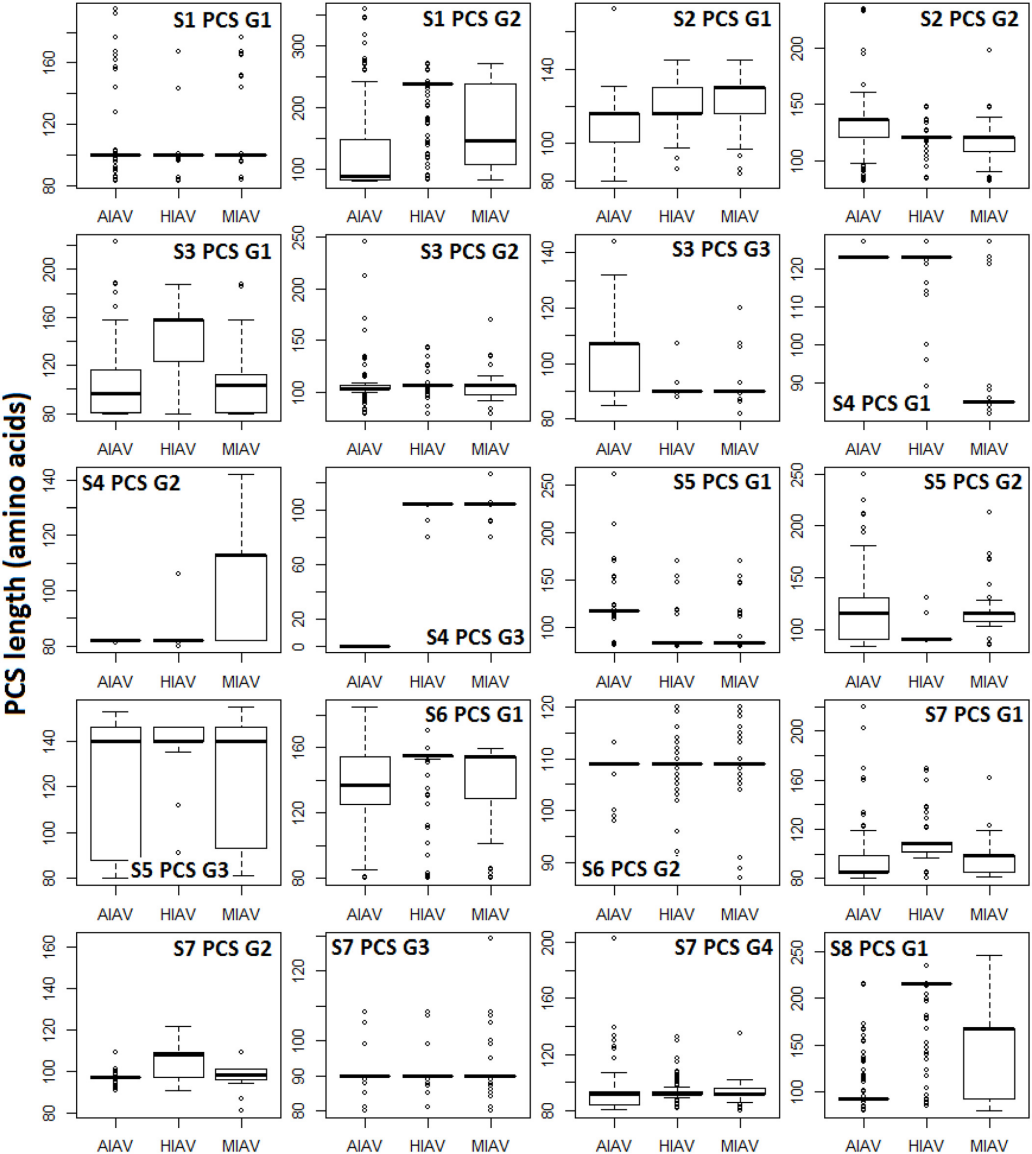
An illustration of the length variations of 20 putative protein coding sequence groups encoded by eight influenza a virus genomic strand RNAs. The y-axis indicates the length of the putative protein coding sequences. AIAV, HIAV and MIAV indicate influenza A viruses from avian, human and mammalian hosts, respectively. One-way ANOVA was performed and p values were used to indicate the differences between the putative protein coding sequence lengths among influenza A viruses from different hosts.

**Fig 3:**
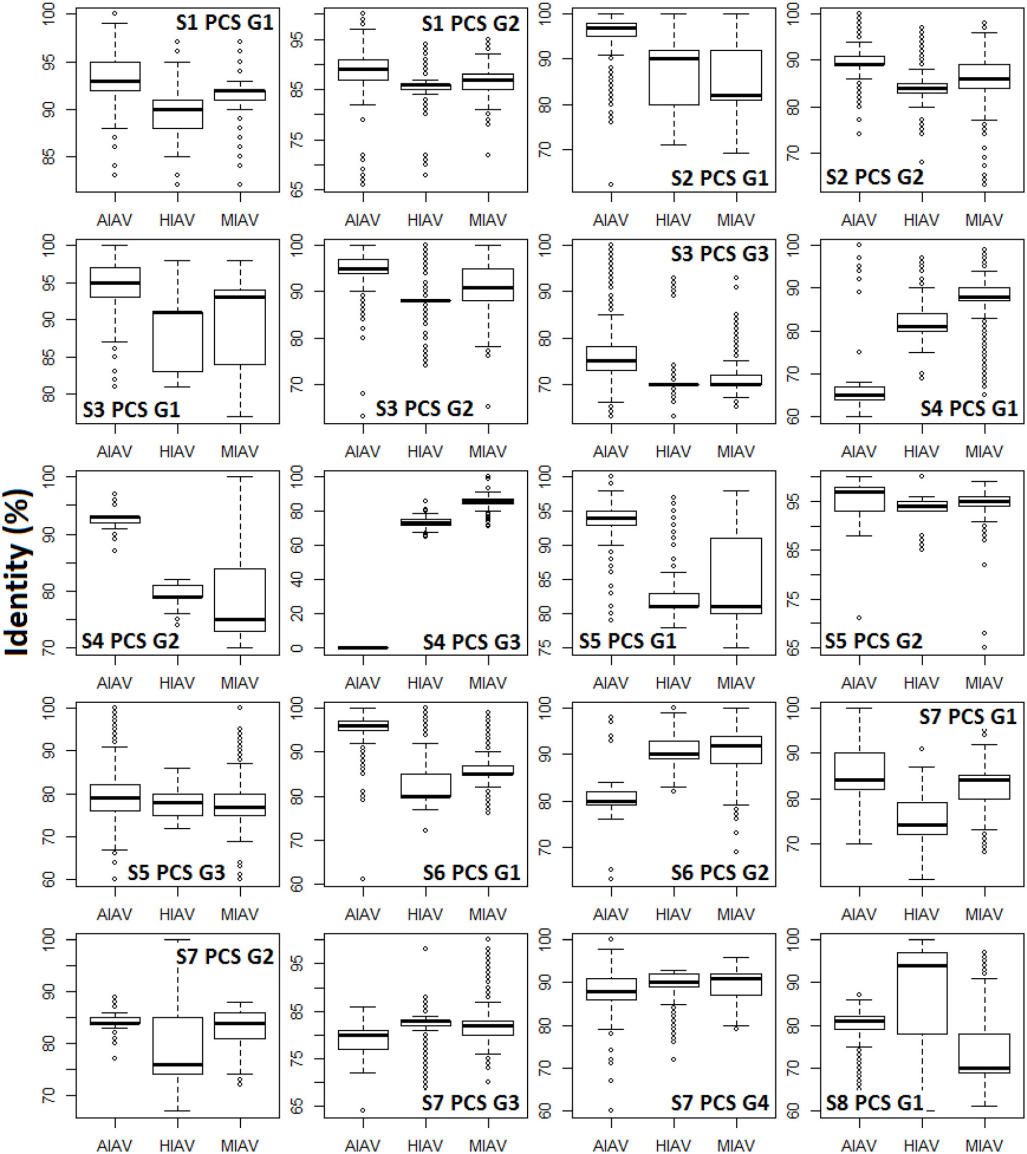
An illustration of the sequence identity variations of 20 putative protein coding sequence groups encoded by eight influenza a virus genomic strand RNAs. The y-axis indicates the sequence identity of putative protein coding sequences. AIAV, HIAV and MIAV indicate influenza A viruses from avian, human and mammalian hosts, respectively. One-way ANOVA was performed and p values were used to indicate the differences between the putative protein coding sequence identities among influenza A viruses from different hosts.

The genomic map of the 20 PCSGs (positions relative to the 10 proteins encoded by positive sense RNAs) is shown in Fig 1. PCSG y in the genomic strand RNA of IAV segment x is represented as Sx PCS Gy in the figures, tables and text in the remainder of the article. The eight ORFs (PB1, PB2, PA, HA, NP, NA, M1 and NS1) in the eight positive sense RNAs were used as reference reading frames (+1 reading frame) for PCSGs encoded by eight genomic strand RNAs. The length and location (start and end) of all PCSGs in the genomic strand RNAs are inconsistent between IAV genomes. Therefore, the genomic map in Fig 1 shows the regions covered by the shortest and longest PCSs of each PCSG in genomic strand RNAs. The length distributions of the 20 PCSGs in AIAVs, HIAVs and MIAVs are shown in Fig 2. S4 PCS G3 was not identified in AIAVs; therefore, the sequence length of S4 PCS G3 of AIAVs is 0 and ANOVA was not performed. Overall, two PCSGs (S5 PCS G2 and S7 PCS G3) have lower length variation (ANOVA p = 5.7E-8 and 3.28E-14) than the other 17 PCSGs (ANOVA p = 0). After sequence clustering, the CD-HIT software chose a longest sequence as a representative sequence for each sequence cluster and computed the identities between the representative sequence and the other sequences within the sequence group. The distributions of sequence identities for each PCSG in AIAVs, HIAVs and MIAVs are shown in Fig 3. S4 PCS G3 was not identified in AIAVs; therefore, the sequence identity of S4 PCS G3 of AIAVs is 0 and ANOVA was not performed. Overall, only three PCSGs (S4 PCS G1, S4 PCS G2 and S6 PCS G2) have lower identity variation (ANOVA p = 1.3E-289, 3.6E-287 and 7.6E-273) than the other 16 PCSGs (ANOVA p = 0).

### Phylogenetic analysis of the 20 PCSGs

The number of sequences in the 20 PCSGs is listed in S2 Table. Because a large amount of sequences cannot be used to perform phylogenetic analysis, protein sequences in each of the 20 PCSGs were further clustered into subgroups based on 95% sequence identity. The number of sequence clusters in the 20 PCSGs is listed in S2 Table. Representative sequences of the subgroups were used to perform multiple sequence alignment followed by phylogenetic analysis. Twenty phylogenetic trees of the corresponding 20 PCSGs are shown in S2 Fig. S7 PCS G2 exhibits the highest sequence to cluster ratio (14945:32), which indicates that this PCSG has the lowest sequence diversity. In contrast, S5 PCS G3 exhibits the lowest sequence to cluster ratio (7169:604), suggesting that this PCSG has the highest sequence diversity.

### Evolving histories of the 20 PCSGs

**Fig 4:**
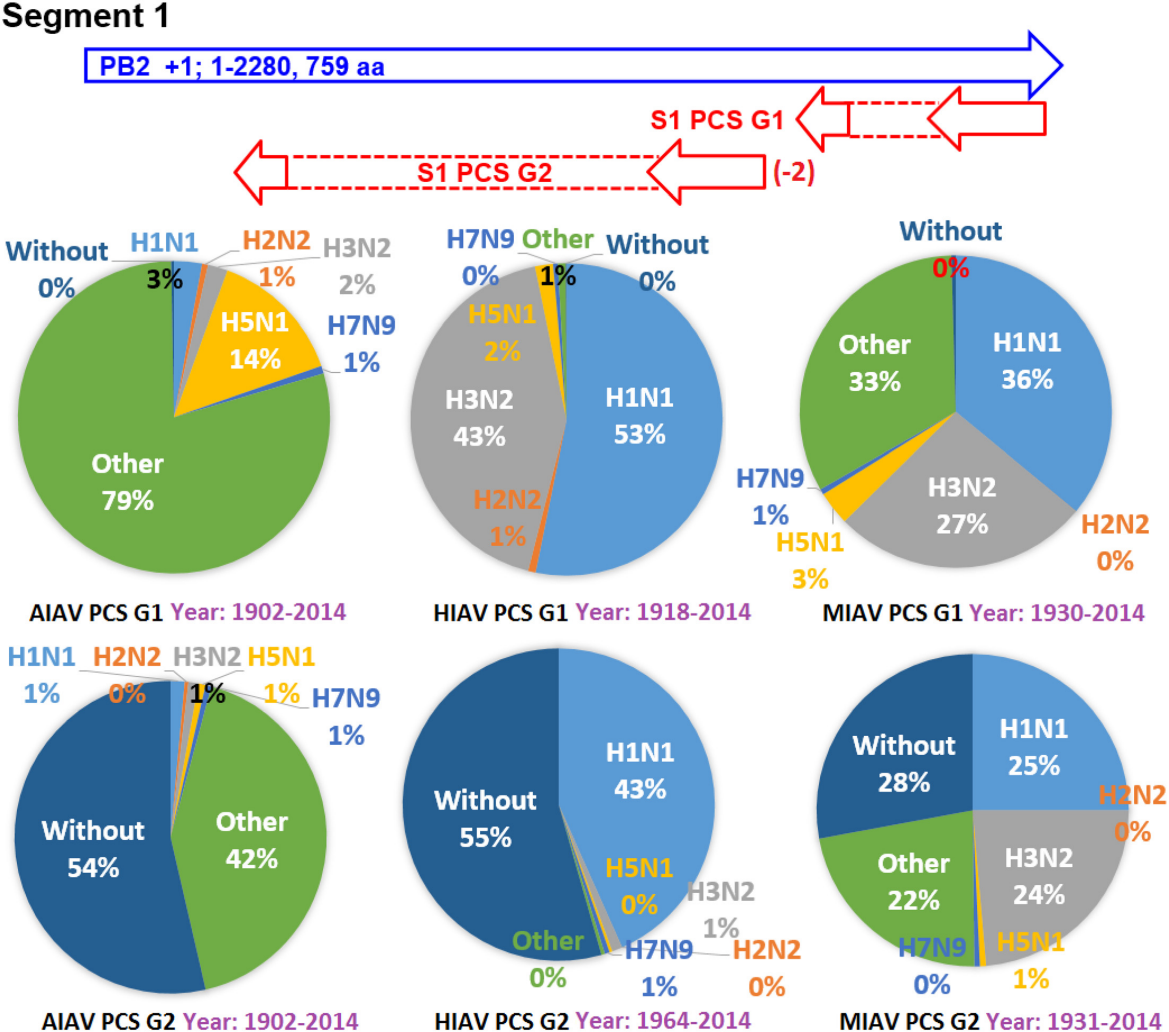
Proportions of putative protein coding sequence groups encoded by the segment 1 genomic strand RNAs of influenza A viruses among different serotypes. Blue arrows indicate open reading frames encoded by segment 1 positive sense RNAs of influenza A viruses. Red arrows indicate putative protein coding sequences encoded by segment 1 genomic strand RNAs of influenza A viruses. AIAV, HIAV and MIAV indicate influenza A viruses from avian, human and mammalian hosts, respectively. The “HxNy” in the pie chart indicates the proportion of segment 1 genomic strand RNAs with putative protein coding sequences that belong to H1N1, H2N2, H3N2, H5N1 or H7N9 serotypes. The “Other” in the pie chart indicates the proportion of segment 1 genomic strand RNAs with putative protein coding sequences that do not belong to the H1N1, H2N2, H3N2, H5N1 or H7N9 serotypes. The “Without” in the pie chart indicates the proportion of segment 1 genomic strand RNAs without a putative protein coding sequence.

**Fig 5:**
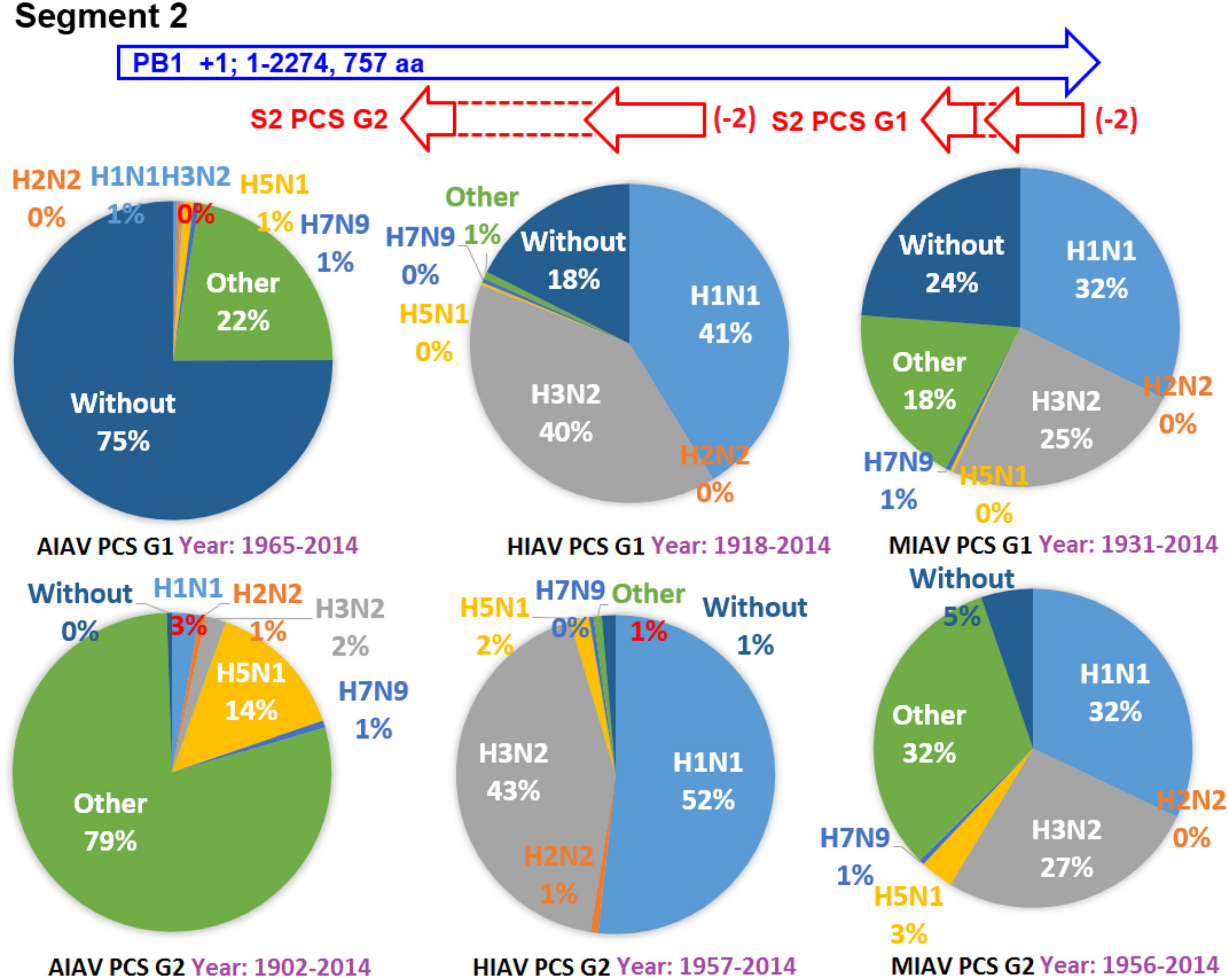
Proportions of putative protein coding sequence groups encoded by the segment 2 genomic strand RNAs of influenza A viruses among different serotypes. Blue arrows indicate open reading frames encoded by segment 2 positive sense RNAs of influenza A viruses. Red arrows indicate putative protein coding sequences encoded by segment 2 genomic strand RNAs of influenza A viruses. AIAV, HIAV and MIAV indicate influenza A viruses from avian, human and mammalian hosts, respectively. The “HxNy” in the pie chart indicates the proportion of segment 2 genomic strand RNAs with putative protein coding sequences that belong to H1N1, H2N2, H3N2, H5N1 or H7N9 serotypes. The “Other” in the pie chart indicates the proportion of segment 2 genomic strand RNAs with putative protein coding sequences that do not belong to the H1N1, H2N2, H3N2, H5N1 or H7N9 serotypes. The “Without” in the pie chart indicates the proportion of segment 2 genomic strand RNAs without a putative protein coding sequence.

**Fig 6:**
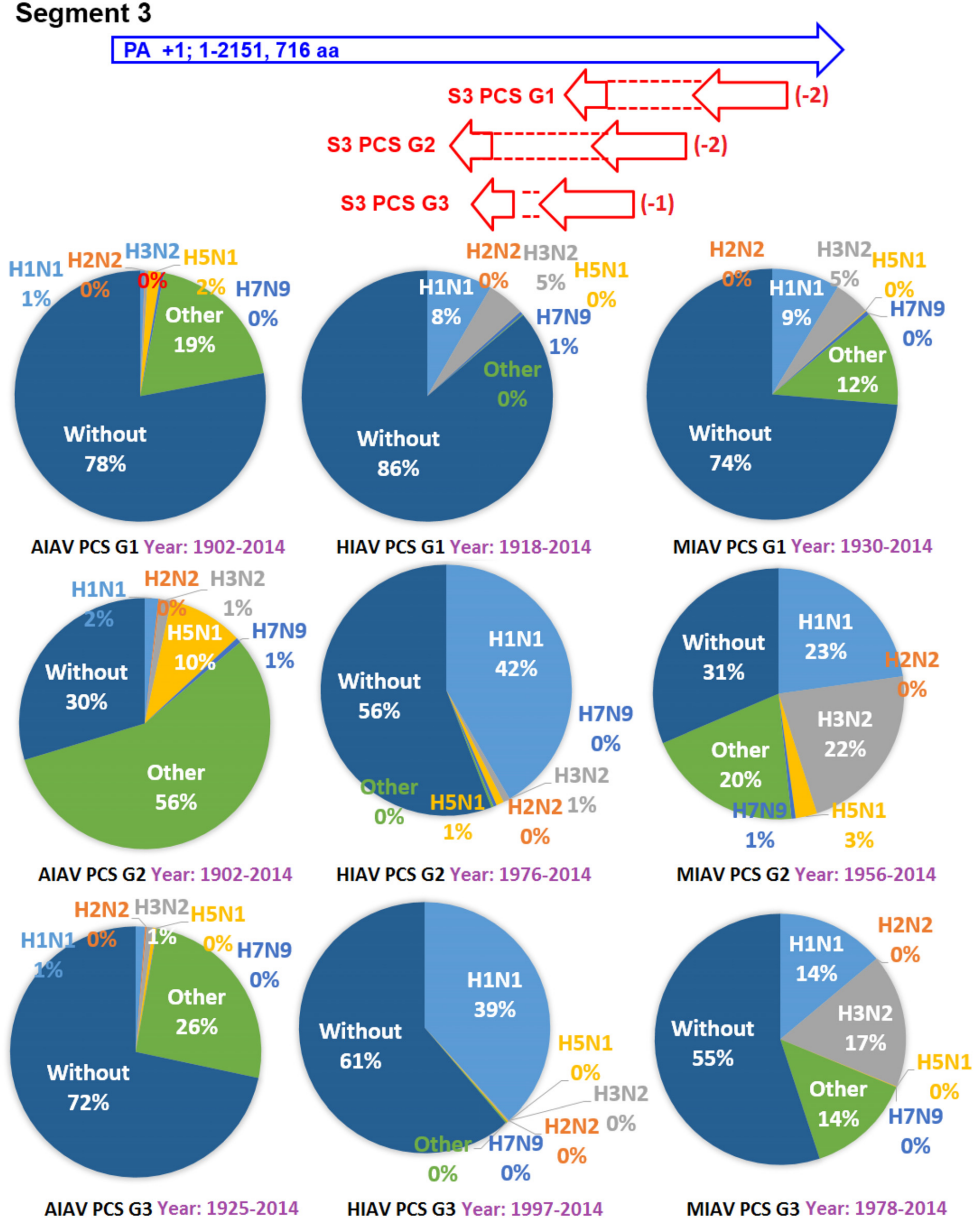
Proportions of putative protein coding sequence groups encoded by the segment 3 genomic strand RNAs of influenza A viruses among different serotypes. Blue arrows indicate open reading frames encoded by segment 3 positive sense RNAs of influenza A viruses. Red arrows indicate putative protein coding sequences encoded by segment 3 genomic strand RNAs of influenza A viruses. AIAV, HIAV and MIAV indicate influenza A viruses from avian, human and mammalian hosts, respectively. The “HxNy” in the pie chart indicates the proportion of segment 3 genomic strand RNAs with putative protein coding sequences that belong to H1N1, H2N2, H3N2, H5N1 or H7N9 serotypes. The “Other” the in pie chart indicates the proportion of segment 3 genomic strand RNAs with putative protein coding sequences that do not belong to the H1N1, H2N2, H3N2, H5N1 or H7N9 serotypes. The “Without” in the pie chart indicates the proportion of segment 3 genomic strand RNAs without a putative protein coding sequence.

**Fig 7:**
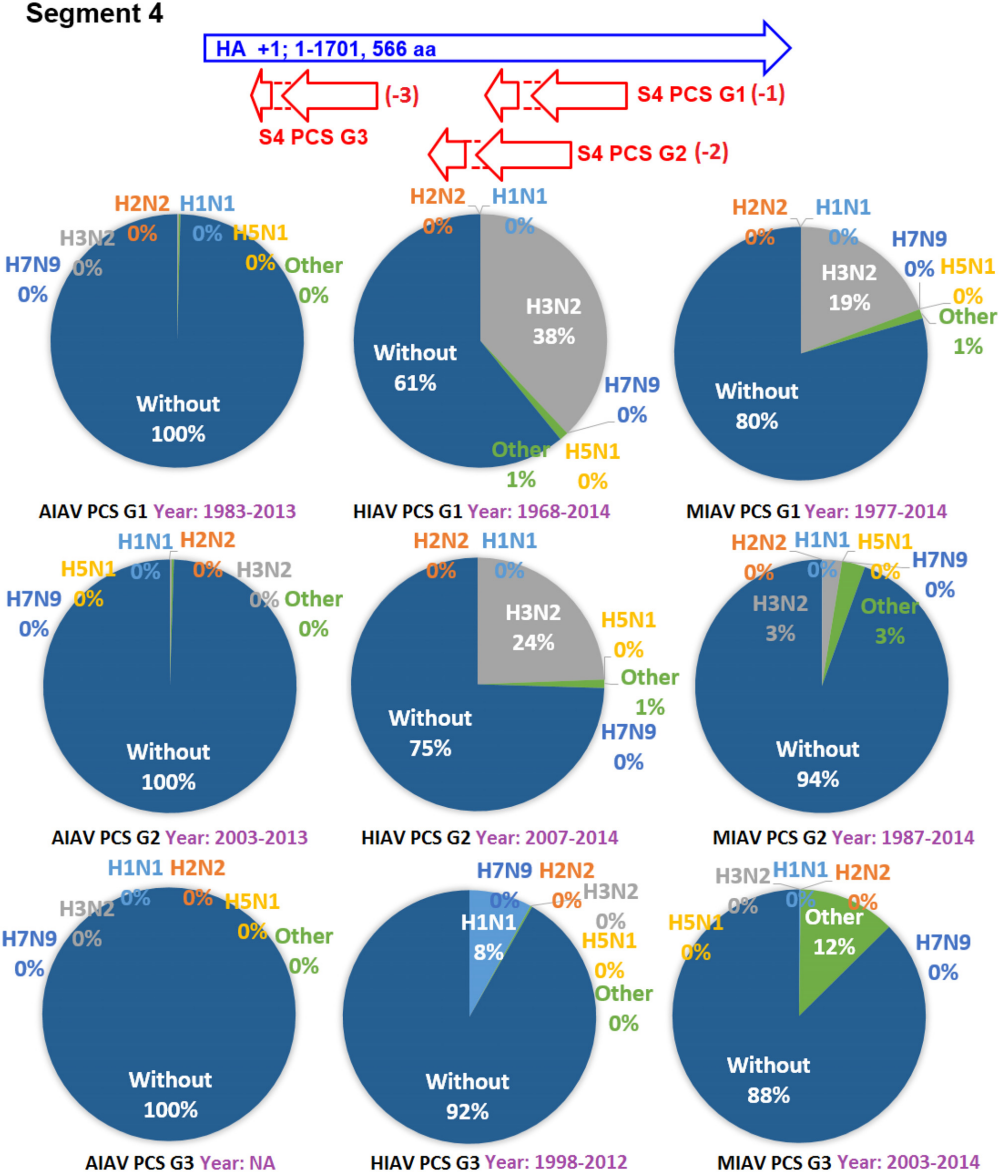
Proportions of putative protein coding sequence groups encoded by the segment 4 genomic strand RNAs of influenza A viruses among different serotypes. Blue arrows indicate open reading frames encoded by segment 4 positive sense RNAs of influenza A viruses. Red arrows indicate putative protein coding sequences encoded by segment 4 genomic strand RNAs of influenza A viruses. AIAV, HIAV and MIAV indicate influenza A viruses from avian, human and mammalian hosts, respectively. The “HxNy” in the pie chart indicates the proportion of segment 4 genomic strand RNAs with putative protein coding sequences that belong to H1N1, H2N2, H3N2, H5N1 or H7N9 serotypes. The “Other” in the pie chart indicates the proportion of segment 4 genomic strand RNAs with putative protein coding sequences that do not belong to the H1N1, H2N2, H3N2, H5N1 or H7N9 serotypes. The “Without” in the pie chart indicates the proportion of segment 4 genomic strand RNAs without a putative protein coding sequence.

**Fig 8:**
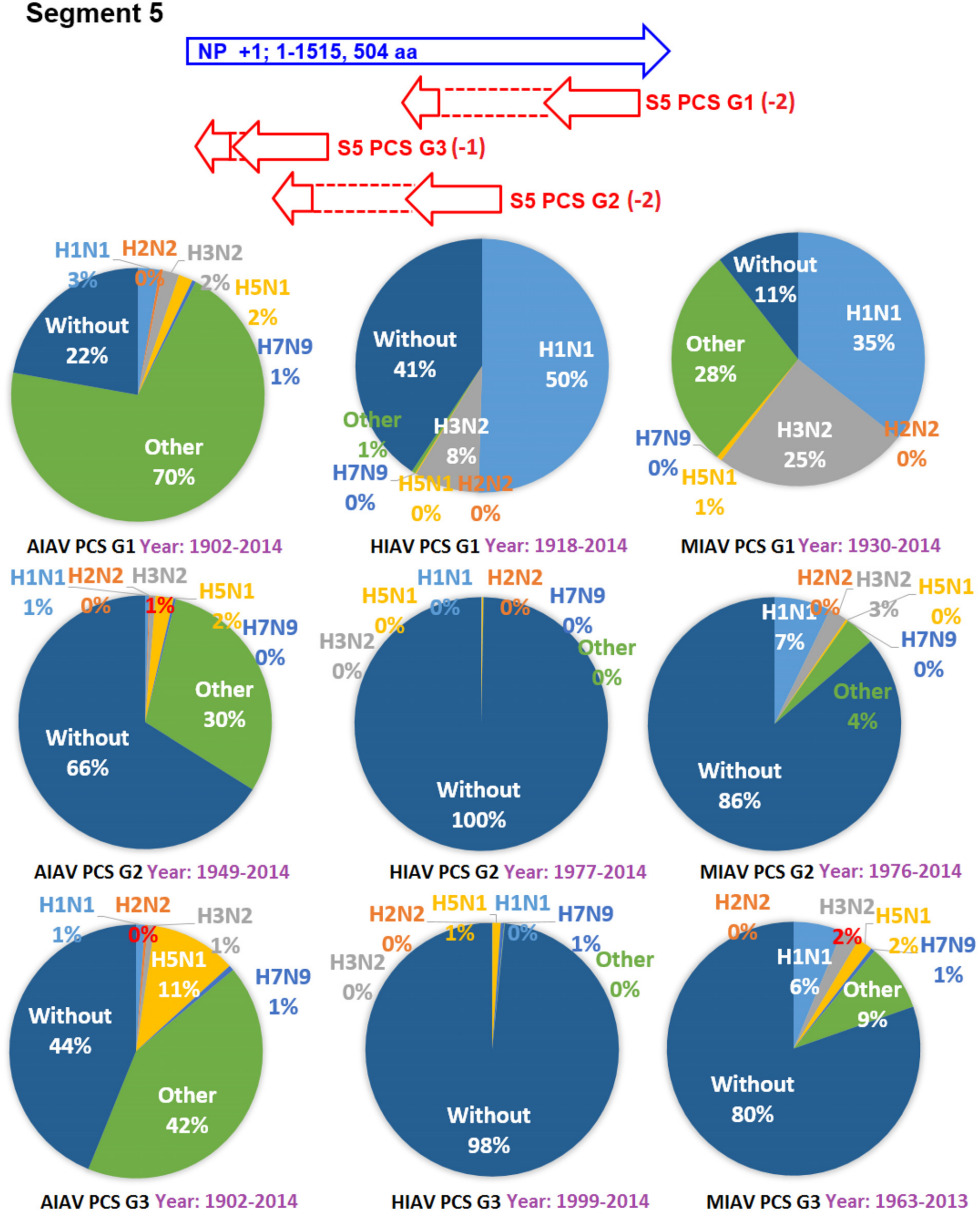
Proportions of putative protein coding sequence groups encoded by the segment 5 genomic strand RNAs of influenza A viruses among different serotypes. Blue arrows indicate open reading frames encoded by segment 5 positive sense RNAs of influenza A viruses. Red arrows indicate putative protein coding sequences encoded by segment 5 genomic strand RNAs of influenza A viruses. AIAV, HIAV and MIAV indicate influenza A viruses from avian, human and mammalian hosts, respectively. The “HxNy” in the pie chart indicates the proportion of segment 5 genomic strand RNAs with putative protein coding sequences that belong to H1N1, H2N2, H3N2, H5N1 or H7N9 serotypes. The “Other” in the pie chart indicates the proportion of segment 5 genomic strand RNAs with putative protein coding sequences that do not belong to the H1N1, H2N2, H3N2, H5N1 or H7N9 serotypes. The “Without” in the pie chart indicates the proportion of segment 5 genomic strand RNAs without a putative protein coding sequence.

**Fig 9:**
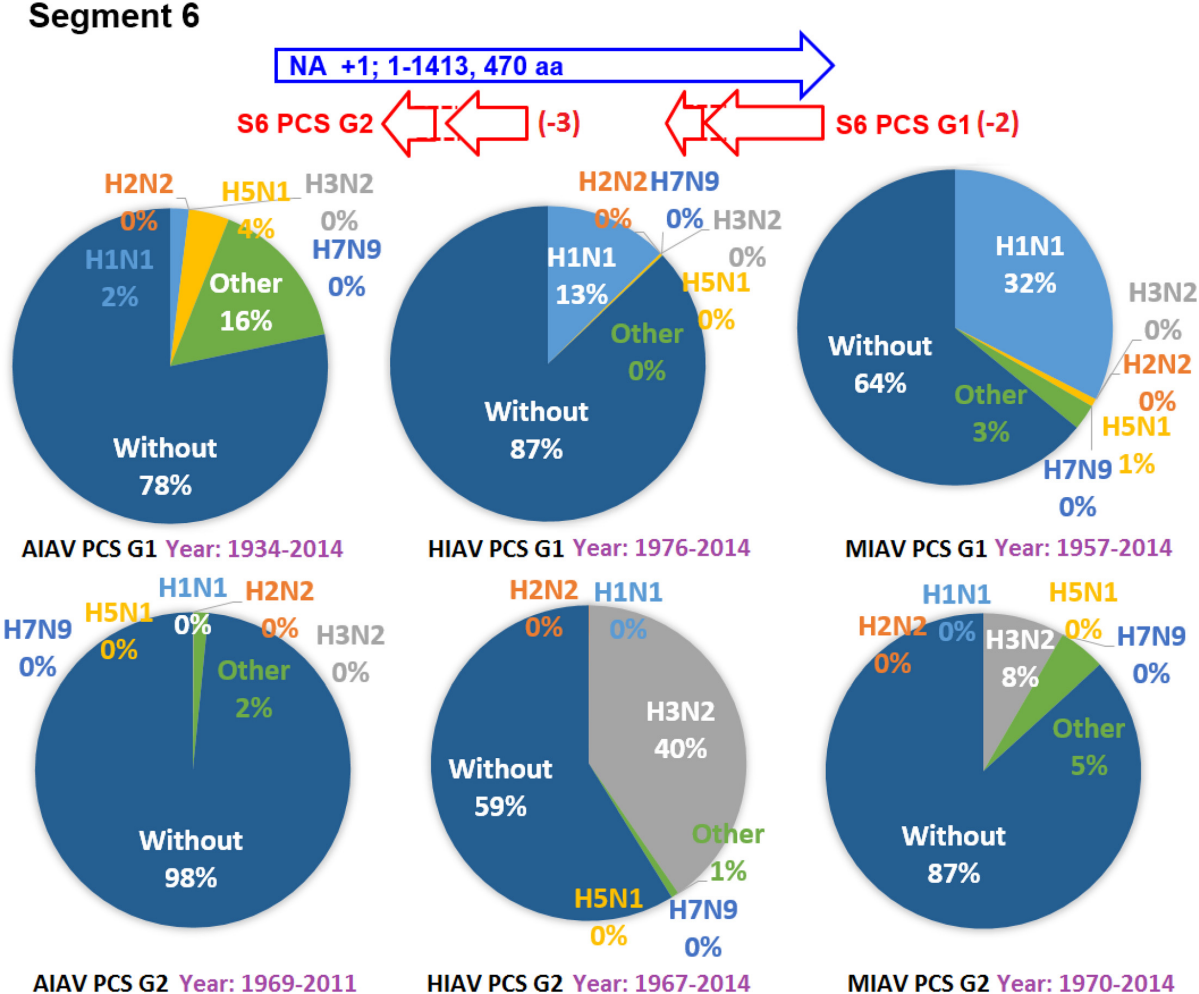
Proportions of putative protein coding sequence groups encoded by the segment 6 genomic strand RNAs of influenza A viruses among different serotypes. Blue arrows indicate open reading frames encoded by segment 6 positive sense RNAs of influenza A viruses. Red arrows indicate putative protein coding sequences encoded by segment 6 genomic strand RNAs of influenza A viruses. AIAV, HIAV and MIAV indicate influenza A viruses from avian, human and mammalian hosts, respectively. The “HxNy” in the pie chart indicates the proportion of segment 6 genomic strand RNAs with putative protein coding sequences that belong to H1N1, H2N2, H3N2, H5N1 or H7N9 serotypes. The “Other” in the pie chart indicates the proportion of segment 6 genomic strand RNAs with putative protein coding sequences that do not belong to the H1N1, H2N2, H3N2, H5N1 or H7N9 serotypes. The “Without” in the pie chart indicates the proportion of segment 6 genomic strand RNAs without a putative protein coding sequence.

**Fig 10:**
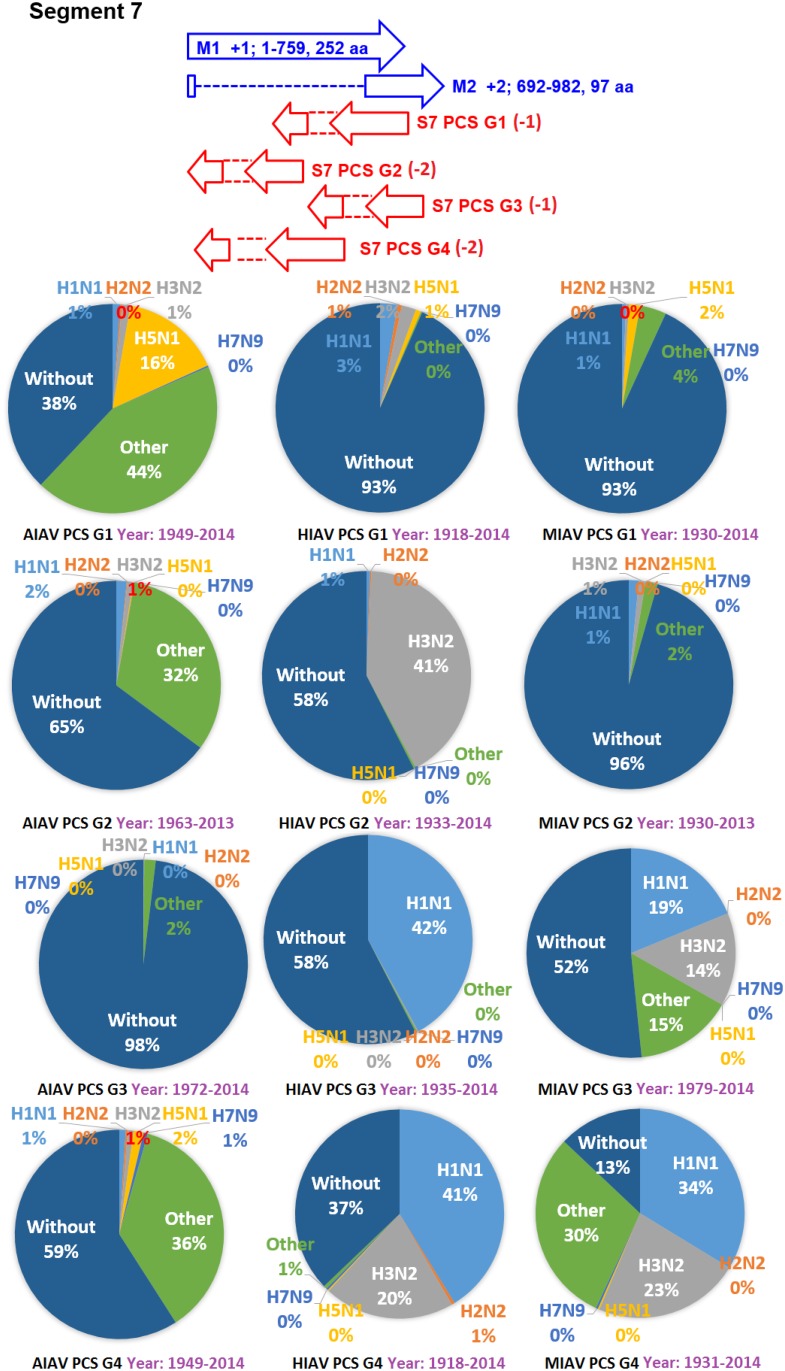
Proportions of putative protein coding sequence groups encoded by the segment 7 genomic strand RNAs of influenza A viruses among different serotypes. Blue arrows indicate open reading frames encoded by segment 7 positive sense RNAs of influenza A viruses. Red arrows indicate putative protein coding sequences encoded by segment 7 genomic strand RNAs of influenza A viruses. AIAV, HIAV and MIAV indicate influenza A viruses from avian, human and mammalian hosts, respectively. The “HxNy” in the pie chart indicates the proportion of segment 7 genomic strand RNAs with putative protein coding sequences that belong to H1N1, H2N2, H3N2, H5N1 or H7N9 serotypes. The “Other” in the pie chart indicates the proportion of segment 7 genomic strand RNAs with putative protein coding sequences that do not belong to the H1N1, H2N2, H3N2, H5N1 or H7N9 serotypes. The “Without” in the pie chart indicates the proportion of segment 7 genomic strand RNAs without a putative protein coding sequence.

**Fig 11:**
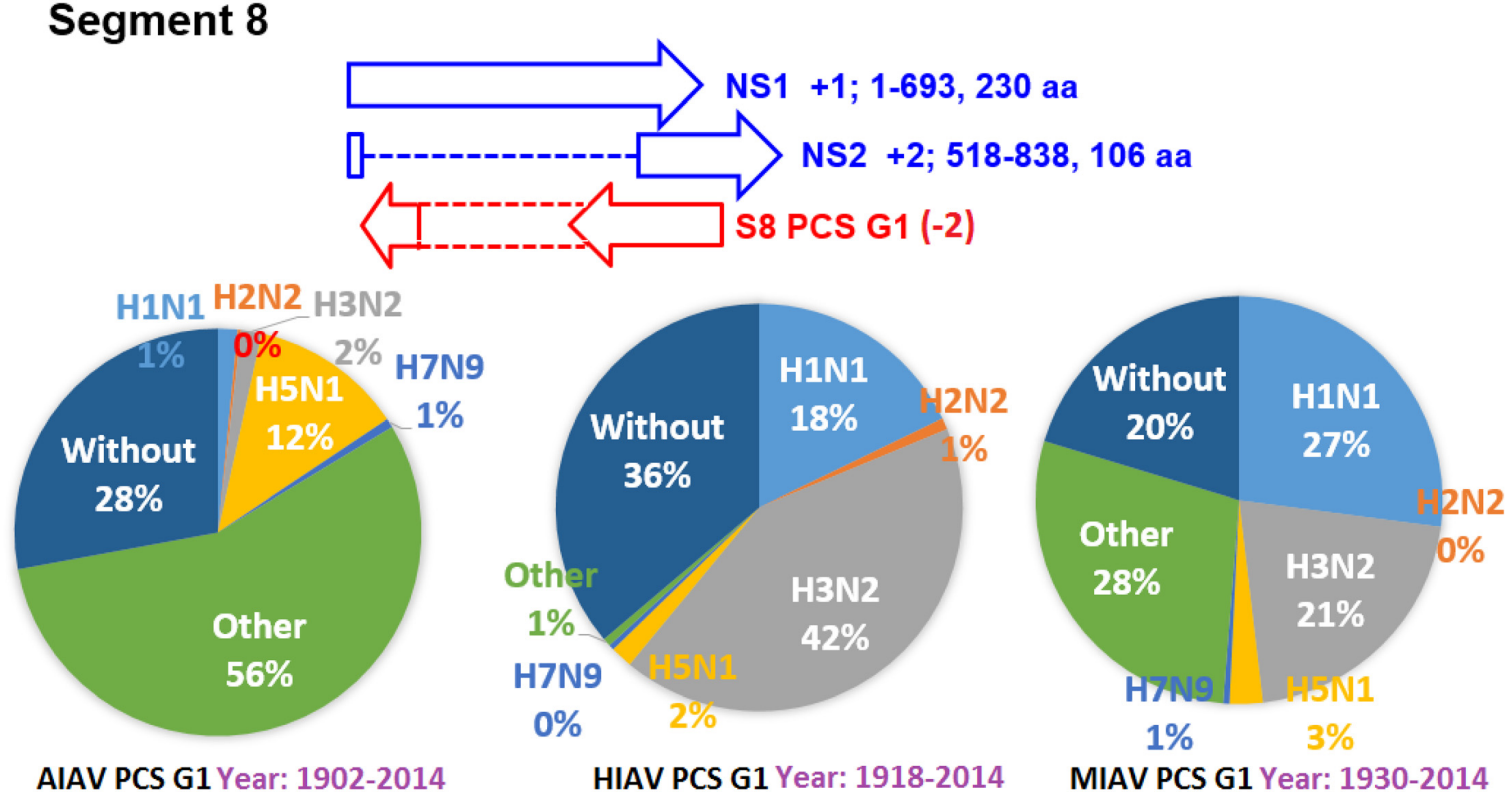
Proportions of putative protein coding sequence groups encoded by the segment 8 genomic strand RNAs of influenza A viruses among different serotypes. Blue arrows indicate open reading frames encoded by segment 8 positive sense RNAs of influenza A viruses. Red arrows indicate putative protein coding sequences encoded by segment 8 genomic strand RNAs of influenza A viruses. AIAV, HIAV and MIAV indicate influenza A viruses from avian, human and mammalian hosts, respectively. The “HxNy” in the pie chart indicates the proportion of segment 8 genomic strand RNAs with putative protein coding sequences that belong to H1N1, H2N2, H3N2, H5N1 or H7N9 serotypes. The “Other” in the pie chart indicates the proportion of segment 8 genomic strand RNAs with putative protein coding sequences that do not belong to the H1N1, H2N2, H3N2, H5N1 or H7N9 serotypes. The “Without” in the pie chart indicates the proportion of segment 8 genomic strand RNAs without a putative protein coding sequence.

The earliest and latest years of the sequences recorded in the NCBI Influenza Virus database for the PCSs of the 20 PCSGs are shown in Figs 4–11. Eight PCSGs (S1 PCS G1, S1 PCS G2, S2 PCS G2, S3 PCS G1, S3 PCS G2, S5 PCS G1, S5 PCS G3 and S8 PCS G1) were present in the records of AIAVs as early as 1902. Four of the eight PCSGs (S1 PCS G1, S3 PCS G1, S5 PCS G1 and S8 PCS G1) were present in the records of HIAVs as early as 1918. Three PCSGs (S2 PCS G1, S7 PCS G1 and S7 PCS G2) were present in the records of HIAVs as early as 1918 but appeared in the records of AIAVs as late as 1949 and 1965. In contrast, S4 PCS G2 was present in the records of AIAVs and HIAVs as late as 2003 and 2007, respectively. S4 PCS G3 was present in the records of MIAVs as late as 2003. Most of PCSGs appeared in the records from 1930 to 1980. Once they appeared, all of the 20 PCSGs were continuously identified in at least one of the AIAVs, HIAVs or MIAVs till 2013 or 2014.

### Proportions of the 20 PCSGs among IAVs from different hosts and serotypes

The proportions of the 20 PCSGs in the AIAVs, HIAVs and MIAVs are shown in Figs 4–11. Eight types of proportion distributions are found. The first PCSG type (order of proportions: AIAVs ≅ MIAVs ≅ HIAVs) has high proportion for all AIAVs, MIAVs and HIAVs. For example, S1 PCS G1 has proportions of 99.8%, 99.99% and 99.5% in the segment 1 RNAs of AIAVs, HIAVs and MIAVs, respectively. Another example is S2 PCS G2, which has proportions of 99.99%, 99.99% and 99.1% in the segment 2 RNAs of AIAVs, HIAVs and MIAVs, respectively. The second PCSG type (order of proportions: AIAVs ≅ MIAVs ≅ HIAVs), composed of S3 PCS G1, has low proportions of 25.4%, 28.77% and 13.8% in the segment 3 RNAs of AIAVs, MIAVs and HIAVs, respectively. The third PCSG type (order of proportions: AIAVs < MIAVs > HIAVs) is composed of two PCSGs (S1 PCS G2 and S7 PCS G4). The fourth PCSG type (order of proportions: AIAVs > MIAVs < HIAVs) is composed of S7 PCS G2. The fifth PCSG type (order of proportions: AIAVs < MIAVs ≅ HIAVs) is composed of three PCSGs (S2 PCS G1, S4 PCS G1 and S7 PCS G3). The sixth PCSG type (order of proportions: AIAVs ≅ MIAVs < HIAVs) is composed of two PCSGs (S4 PCS G2 and S6 PCS G2). The seventh PCSG type (order of proportions: AIAVs > MIAVs ≅ HIAVs) is composed of two PCSGs (S5 PCS G3 and S7 PCS G1). The eighth PCSG type (order of proportions: AIAVs ≅ MIAVs > HIAVs) is composed of two PCSGs (S3 PCS G2 and S5 PCS G1).

The proportions of the 20 PCSGs in the IAVs of different serotypes from different hosts are shown in Figs 4–11. Five PCSGs (S1 PCS G2, S3 PCS G2, S3 PCS G3, S5 PCS G1 and S7 PCS G3) have higher proportions from H1N1 than other serotypes in the HIAVs. In contrast, five PCSGs (S4 PCS G1, S4 PCS G2, S6 PCS G2, S7 PCS G2 and S8 PCS G1) have higher proportions from H3N2 than other serotypes in the HIAVs.

### PCSs encoded by the genomic RNAs of pandemic, outbreak and highly pathogenic IAVs

The PCSs encoded by the genomic strand RNAs of pandemic, outbreak and highly pathogenic IAVs from human hosts are listed in Table 1. The PCSs encoded by the genomic strand RNAs of H1N1 WSN33 and H1N1 PR8 HIAVs are also listed as reference IAV strains (frequently used in laboratory experiments). The genomic maps of these PCSs are shown in S3 Fig. Three PCSGs are worth noting. First, a 239-amino acid PCS belonging to S1 PCS G2 is present in segment 1 of H1N1 from the 2009 swine flu. Another 174-amino acid PCS belonging to S1 PCS G2 is present in segment 1 of the H5N1 HK 1997 and H7N9 2013 HIAVs. Second, a 109-amino acid PCS belonging to S3 PCS G2 is present in segment 3 of H1N1 1918 HIAV. Shorter forms of 95~98-amino acids PCSs belonging to S3 PCS G2 are also present in segment 3 of the H5N1 and H7N9 2013 HIAVs. Third, a 154-amino acid PCS belonging to S6 PCS G1 is present in segment 6 of the H5N1 HIAVs from Indonesia, Thailand and Vietnam. As shown in Figs 4, 6 and 9, the three PCSGs have higher proportions of H1N1 HIAVs than other serotypes. An additional PCSG which does not belong to the 20 PCSGs was found encoded in the segment 5 genomic strand RNAs of H5N1 Hong Kong 1997 (A/Hong Kong/156/97(H5N1)) (page 5 in S3 Fig). The proportions of this PCSG are 180/11165 (0.0161), 33/12806 (0.0026) and 48/3535 (0.0136) in AIAVs, HIAVs and MIAVs, respectively.

**Table 1:** Length (amino acids) of the putative protein coding sequences identified in the genomic strand RNAs of pandemic, outbreak and highly pathogenic HIAVs.

	H1N1 1918	H1N1 2009	H2N2 1957	H3N2 1968	H5N1 HK	H5N1 IN	H5N1 TL	H5N1 VN	H7N9 2013	H1N1WSN33	H1N1 PR8
S1 PCS G1	100	100	100	100	100	100	100	100	85*	100	100
S1 PCS G2		239			174				174		
S2 PCS G1	130	116			116				101	130	
S2 PCS G2		121	121	121	137	101	137	137	137		
S3 PCS G1									81		
S3 PCS G2	109						98	98	95	102	109
S3 PCS G3		90									
S4 PCS G1											
S4 PCS G2											
S4 PCS G3											
S5 PCS G1	83	83			117					83	83
S5 PCS G2											
S5 PCS G3											
S6 PCS G1					81	154	154	154	91		
S6 PCS G2			87	87							
S7 PCS G1	108,99	90	108,109	109		85	85	85	84	102	99
S7 PCS G2											
S7 PCS G3											
S7 PCS G4					84						
S8 PCS G1	167	85	216	216	93	93	93	93	93	167	167

S1, S2, … S8 in the first column represent Segment 1 … Segment 8 of the influenza A virus genomes.

PCSG: putative protein coding sequence group.

H1N1 1918: Influenza A virus (A/Brevig Mission/1/1918(H1N1))

H1N1 2009: Influenza A virus (A/Mexico/LaGloria-8/2009(H1N1))

H2N2 1957: Influenza A virus (A/Guiyang/1/1957(H2N2)). Segment 4 of (A/Guiyang/1/1957(H2N2)) is not complete and was replaced with segment 4 of Influenza A virus (A/Singapore/1/1957(H2N2)).

H3N2 1968: Influenza A virus (A/Hong Kong/1/1968(H3N2))

H5N1 HK: Influenza A virus (A/Hong Kong/481/97(H5N1))

H5N1 IN: Influenza A virus (A/Indonesia/283H/2006(H5N1))

H5N1 TL: Influenza A virus (A/Thailand/1(KAN-1)/2004(H5N1))

H5N1 VN: Influenza A virus (A/Viet Nam/1203/2004(H5N1))

H7N9 2013: Influenza A virus (A/Shanghai/02/2013(H7N9))

H1N1 WSN 33: Influenza A virus (A/WSN/1933(H1N1))

H1N1 PR8: Influenza A virus (A/Puerto Rico/8/1934(H1N1))

* indicates N-terminal truncated form without the predicted signal sequence.

### Protein function domains predicted *in silico*

**Fig 12:**
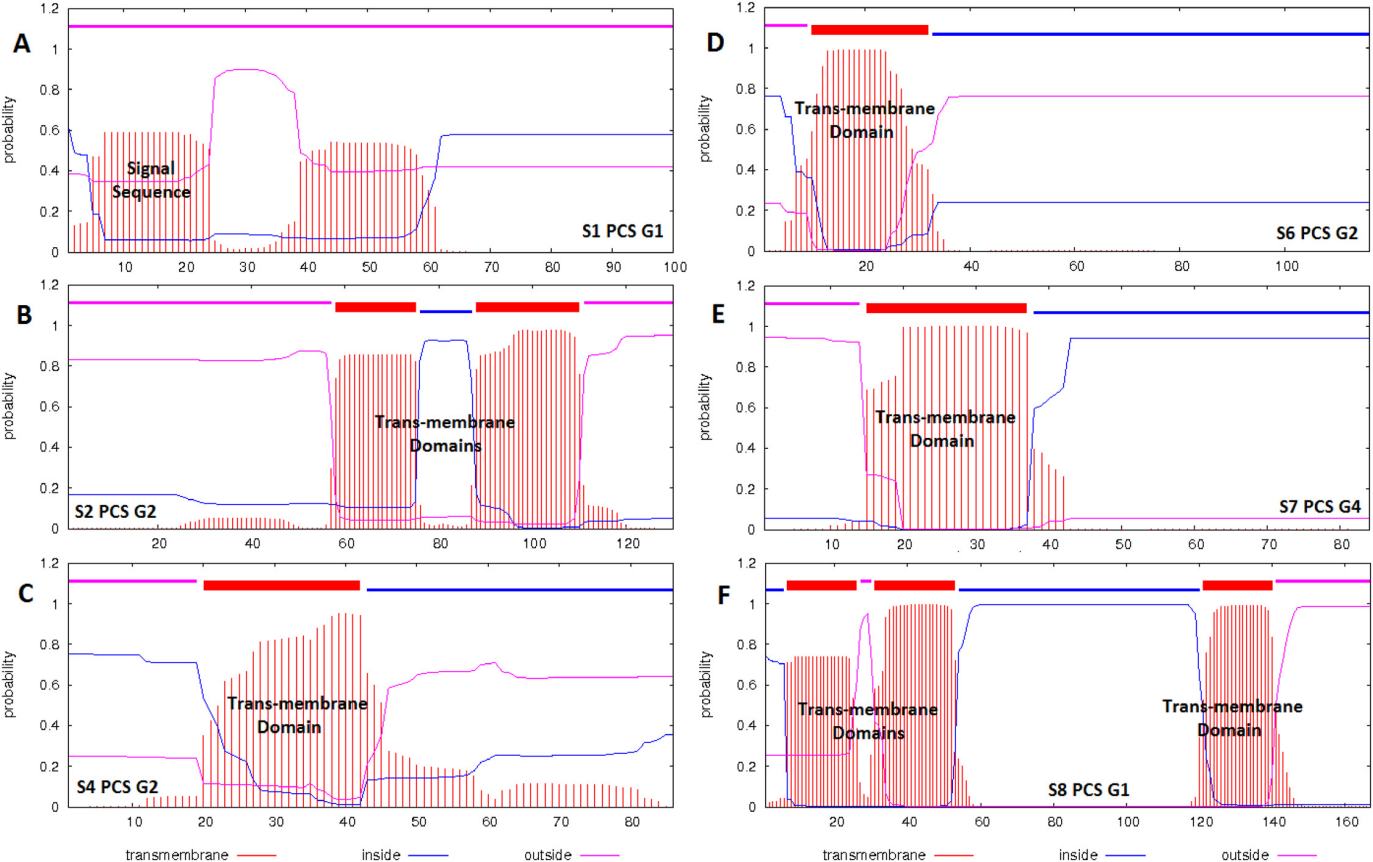
Prediction of signal sequences and trans-membrane domains. The y-axis indicates the probability of prediction. An N-terminal sequence with probability between 0.6 and 0.7 may be interpreted as a signal sequence. An internal sequence with a probability greater than 0.7 may be interpreted as a trans-membrane domain.

Signal sequences (for the protein secretory pathway) and trans-membrane domains were predicted in six PCSGs (S1 PCS G1, S2 PCS G1, S4 PCS G2, S6 PCS G2, S7 PCS G4 and S8 PCS G1) *in silico* (Fig 12). A signal sequence (and no trans-membrane domain) was identified in S1 PCS G1, suggesting that S1 PCS G1 may be a secretory protein (Fig 12A). Trans-membrane domains (and no signal sequence) were identified in S2 PCS G1, S4 PCS G2, S6 PCS G2, S7 PCS G4 and S8 PCS G1 suggesting that these PCSGs may be encoded membrane proteins on organelle or plasma membranes (Fig 12B–12F). Except for the signal sequences and trans-membrane domains in the three PCSGs, no protein function domain was identified in the 20 PCSGs using the SMART Database and NCBI Conserved Domain Database.

### Positional overlapping between critical amino acids in the proteins encoded by the positive strand RNAs and 20 PCSGs encoded by the genomic strand RNAs

**Fig 13:**
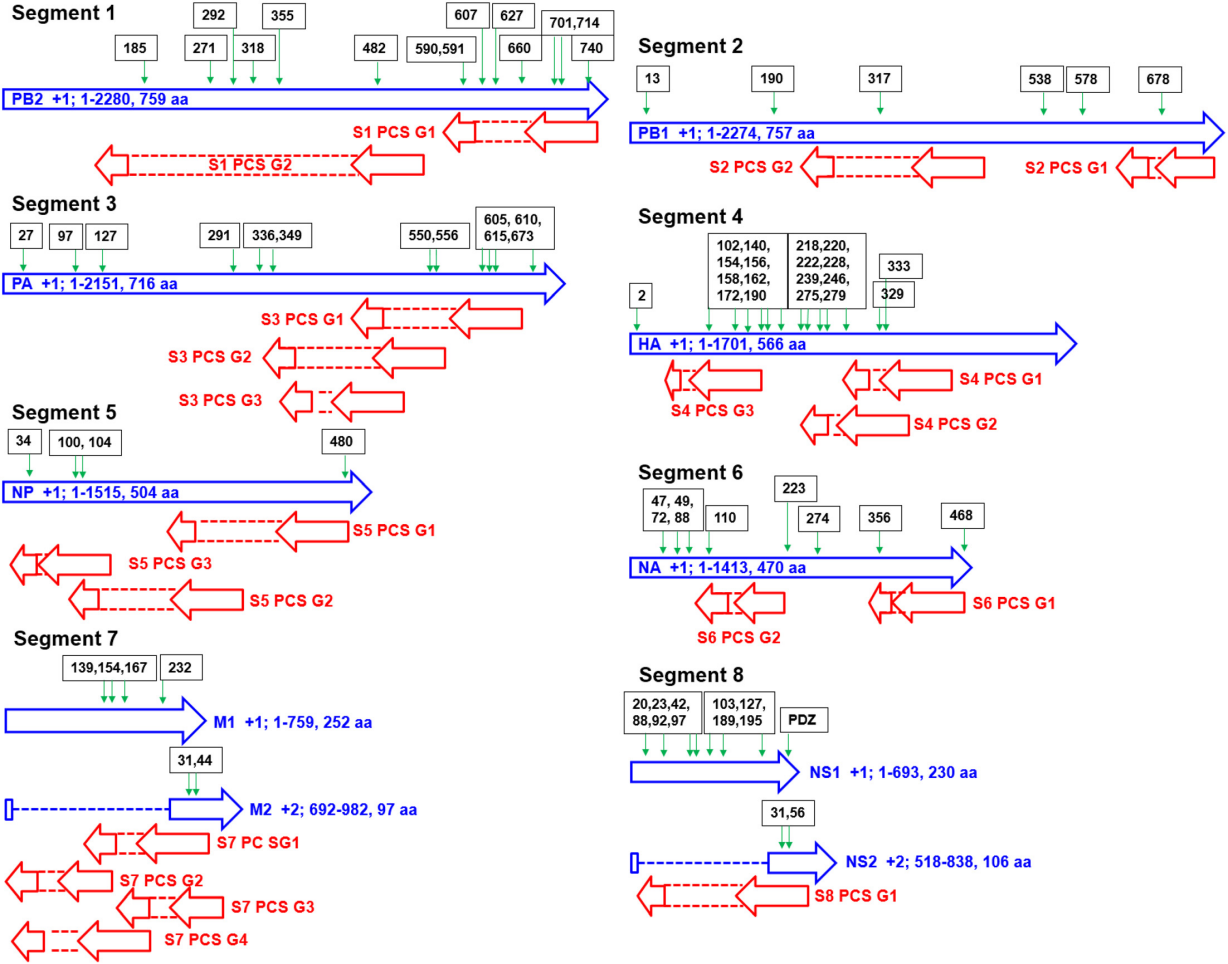
Map of the positional overlapping of previously reported amino acids associated with virulence in the proteins encoded by positive sense RNAs and the 20 PCSGs encoded by genomic strand RNAs. Amino acid sites in this figure are summarize from reference 19–26.

**Fig 14:**
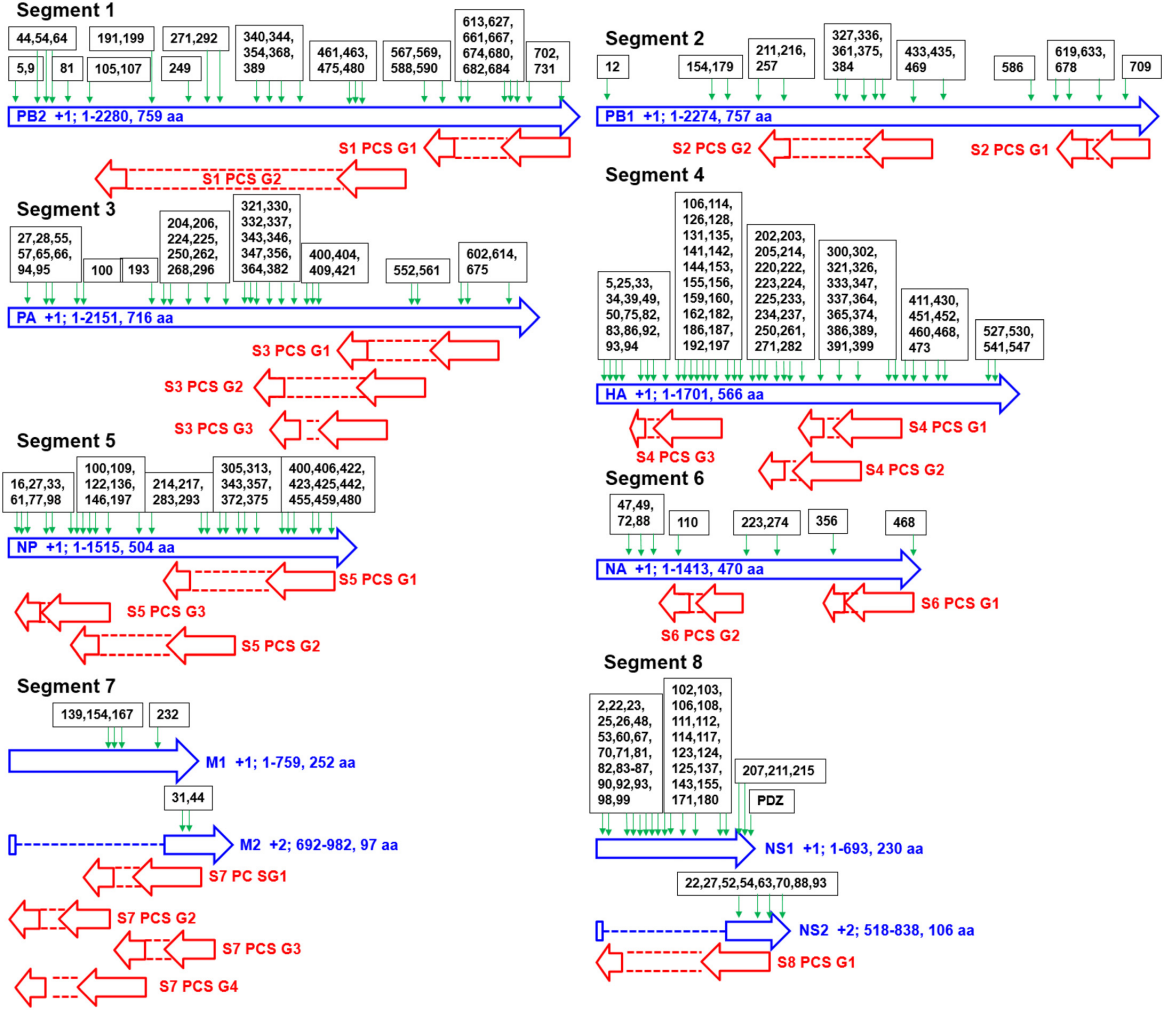
Map of the positional overlapping of previously reported amino acids used as genetic markers and associated with host adaptation in the proteins encoded by positive sense RNAs and the 20 PCSGs encoded by genomic strand RNAs. Amino acid sites in this figure are summarize from reference 27–40.

Several amino acids in the proteins encoded by positive sense RNAs have been reported to be associated with virulence and host adaptation and can be used as genetic markers. Many of these amino acid sites overlap the 20 PCSGs identified in this study. Maps of the positional overlapping of the critical amino acids, which in the proteins encoded by positive sense RNAs are associated with virulence [18–26], with the 20 PCSGs (53 amino acid sites) are shown in Fig 13. Maps of the positional overlapping of critical amino acids, which in the proteins encoded by positive sense RNAs are associated with genetic markers and host adaptation [27–40], with the 20 PCSGs (198 amino acid sites) are shown in Fig 14.

## Discussion

In this study, 20 PCSGs were proposed to be encoded by the genomic strand of IAV RNAs. If the prediction is true, it has several implications. The first and simplest consequence is that IAV genome segments are “ambisense”. Originally, the term “ambisense” was used to describe the coding strategies of arenaviruses (family Arenaviridae) and members of the *Phlebovirus* genus of the Bunyaviridae in that some proteins are encoded by viral-complementary RNA sequences and others are encoded by the viral RNA sequence [41]. In those cases, ORFs are not overlapped. In contrast, the 20 IAV PCSGs proposed in this study overlapped with ORFs encoded by positive sense RNAs. Overlapping coding sequences may undergo co-evolution in a sophisticated manner. Amino acids encoded by overlapping coding sequences are structurally, functionally, and co-evolutionarily constrained.

The distribution of the length of the 5'-UTR in human mRNA is between 100 to 500 bp [42]. As shown in Fig 1, eight PCSGs (S1 PCS G1, S2 PCS G1, S3 PCS G1, S5 PCS G1, S6 PCS G1, S7 PCS G1, S7 PCS G2 and S8 PCS G1) have a start codon near from the 5'-end (eg. shorter than 500 bp) of the IAV genomic strand RNAs. The PCSGs near the middle and 3'-end of the genomic strand RNAs may either need a mechanism for internal translation initiation for protein synthesis or have a very low efficiency of translation initiation and protein synthesis activities. Alternatively, they may form a reservoir of coding sequences. These potential coding sequences may provide additional protein motifs for coding sequences near the 5’-end, whereas frame shift mutations occur or new splicing sites are generated by random mutations.

The difference in the host adaption and virulence between IAVs may be derived from the different composition of the viral core and pan proteomes. The 10 well-studied viral proteins (PB2, PB1, PA, HA, NP, NA, M1, M2, NS1 and NS2) comprise the core proteome of IAVs. Sequence and length variations of IAV proteins in the core proteome among different virus strains may lead to function or activity diversity of proteins and differences in virulence among IAVs. For example, the C-terminal PDZ domain of the NS1 protein is associated with the virulence of IAVs. IAVs with C-terminal deletion of the NS1 protein exhibit relatively lower pathogenicity than IAVs harboring NS1 proteins with the C-terminal PDZ domain [23]. In contrast, based on the counts of CDSs from the NCBI Influenza A Virus Database, the frequencies of PB1-F2 protein are 1, 0.76 and 0.61 for AIAV, MIAV and HIAV, respectively. The PB1-F2 protein may be considered a protein belongs to the pan proteome of IAVs. The PB1-F2 protein was reported to exhibit contributions to IAV pathogenesis in mice [43,44,45]. The lower frequency of the PB1-F2 protein in MIAVs and HIAVs than in AIAVs may suggest a disadvantage of the protein for IAVs in mammalian and human hosts. Similarly, the frequencies of PCSGs among AIAVs, MIAVs and HIAVs suggest that the functions of proteins encoded by these PCSGs may be associated with host adaptation (Figs 4–11). For example, S1 PCS G1 has frequencies of 99.8%, 99.99% and 99.5% in the segment 1 genomic strand RNAs of AIAVs, HIAVs and MIAVs, respectively. The function of the protein encoded by this PCSG may have large advantages for viral replication or survival. S2 PCS G1 has frequencies of 24.9%, 82.4% and 76.2% in the segment 2 genomic strand RNAs of AIAVs, HIAVs and MIAVs, respectively. The function of the protein encoded by this PCSG may have more advantages for viral adaptation in mammalian and human hosts than in avian hosts. Similarly, the function of the protein encoded by S7 PCS G3 may also have greater advantages for viral adaptation in mammalian and human hosts than in avian hosts (Fig 10). Alternatively, the function of the protein encoded by S7 PCS G1 may have greater advantages for viral adaptation in avian hosts than in mammalian and human hosts (Fig 10). The contributions of the 20 PCSGs to viral evolution, host adaptation and pathogenicity are worth further investigation.

The sequence and length variations of the 20 PCSGs among different virus strains may also lead to function or activity diversity of proteins synthesized from these PCSGs. For instance, the protein encoded by S1 PCS G1 has a predicted signal sequence (approximately 21 amino acids in length) and may be a secretory protein. However, the H7N9 2013 HIAV has an N-terminal deletion (15 amino acids in length) in the S1 PCS 1 protein. Whether this deletion leads to the accumulation of S1 PCS 1 protein inside cells and results in cell damages is worth further investigation.

## Conclusions

The results of this study suggest the possibility of the ambisense nature of IAV genomes. A potential reservoir encoding the pan proteome may exist in the genomic strand RNAs of IAVs. The composition variations of the pan proteome (such as with or without the PB1-F2 protein) among IAV strains may contribute to viral evolution, host adaptation and pathogenicity.

## Supporting Information

S1 Fig A current model of the influenza A virus core proteome. (PDF)

S2 Fig Phylogenetic trees of representative sequences from 20 putative protein coding sequence groups encoded by genomic strand RNAs of the influenza A viruses. (PDF)

S3 Fig Genomic maps of 20 putative protein coding sequences encoded by eight genomic strand RNAs of pandemic, outbreak and highly pathogenic HIAVs. (PDF)

S1 Table Number of IAV genomic RNA sequences used in this study. (DOC)

S2 Table Number of sequences and sub-clusters of the 20 putative protein coding sequence groups. (DOC)
