# Supplementary material for: Uncovering the Potential Pan Proteomes Encoded by Genomic Strand RNAs of Influenza A Viruses
Source: PLoS One. 2016 Jan 13;11(1):e0146936. doi: 10.1371/journal.pone.0146936 (PMC4711952; doi:10.1371/journal.pone.0146936)
Supplement: S1 Fig — (PDF) [file pone.0146936.s001.pdf]

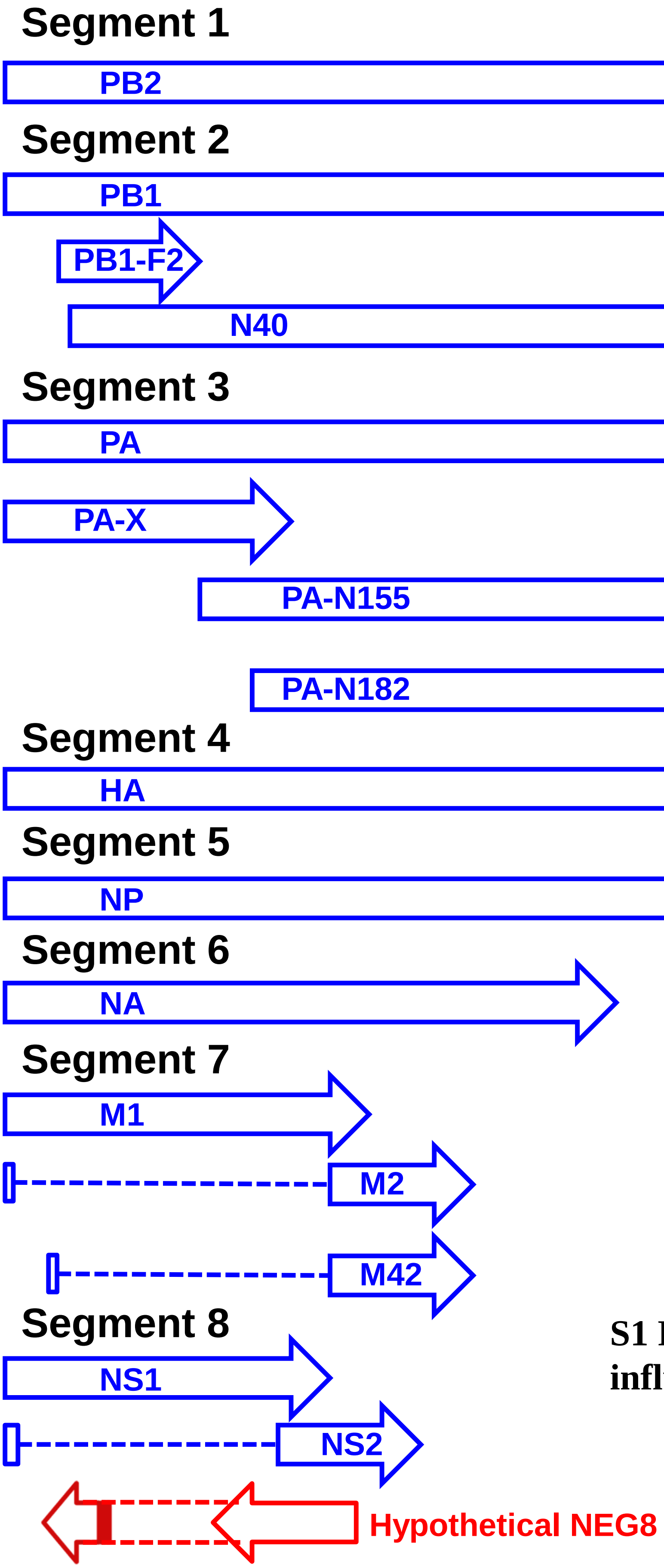

S1 Fig. A current model of the influenza A virus core proteome.
