# Supplementary material for: Uncovering the Potential Pan Proteomes Encoded by Genomic Strand RNAs of Influenza A Viruses
Source: PLoS One. 2016 Jan 13;11(1):e0146936. doi: 10.1371/journal.pone.0146936 (PMC4711952; doi:10.1371/journal.pone.0146936)
Supplement: S2 Fig — (PDF) [file pone.0146936.s002.pdf]

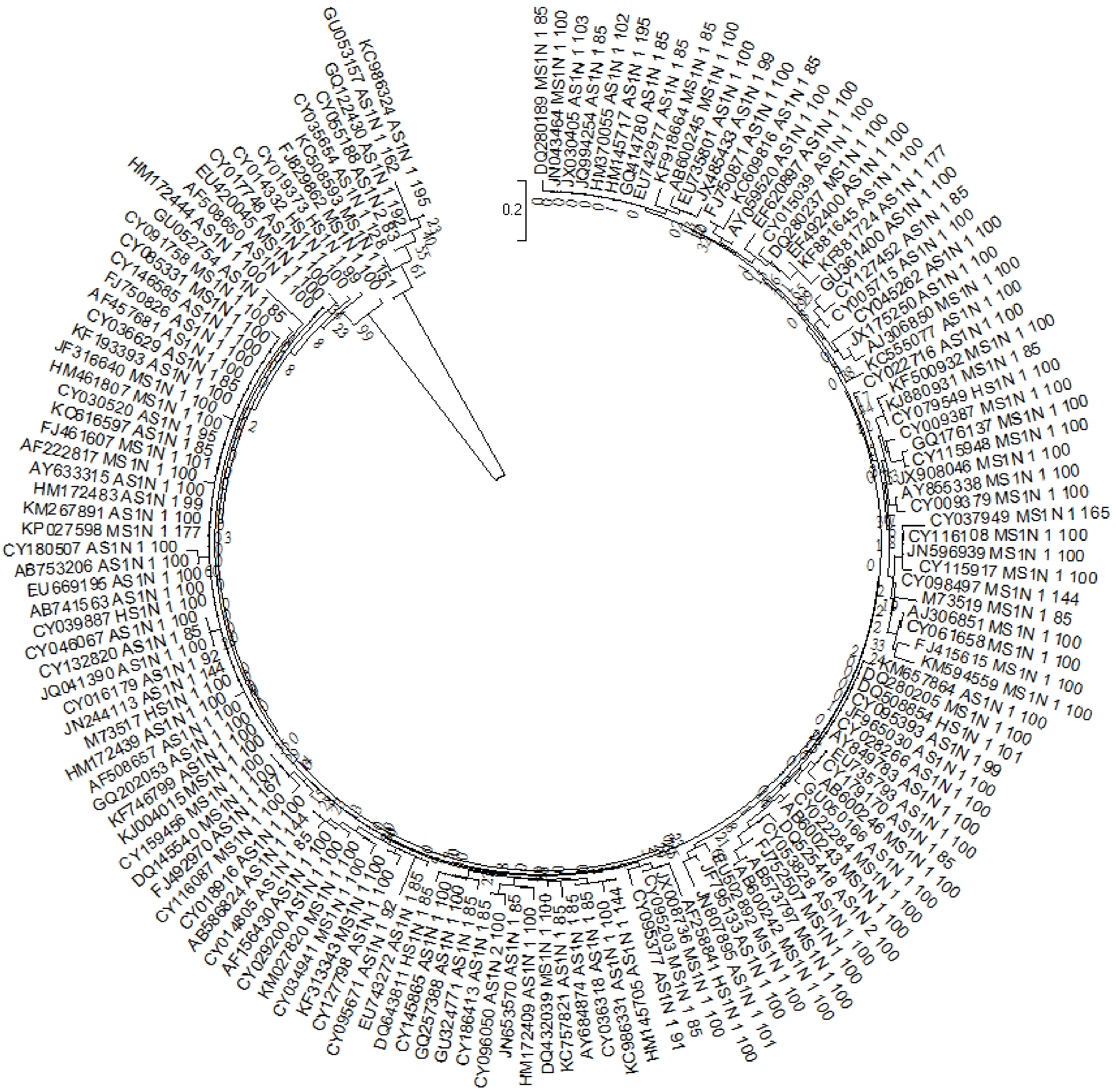

Figure 1. Phylogenetic tree of 149 representative sequences from 149 groups of S1 PCS G1. In total 26,527 protein sequences from S1 PCS G1 of genomic strand of IAV segment 1 were clustered into 149 subgroups by CD-HIT software based on 95% sequence identity. Phylogenetic tree of 149 representative sequences was constructed using the Neighbor joining method with bootstrapping 1000 times by MEGA 6.0.

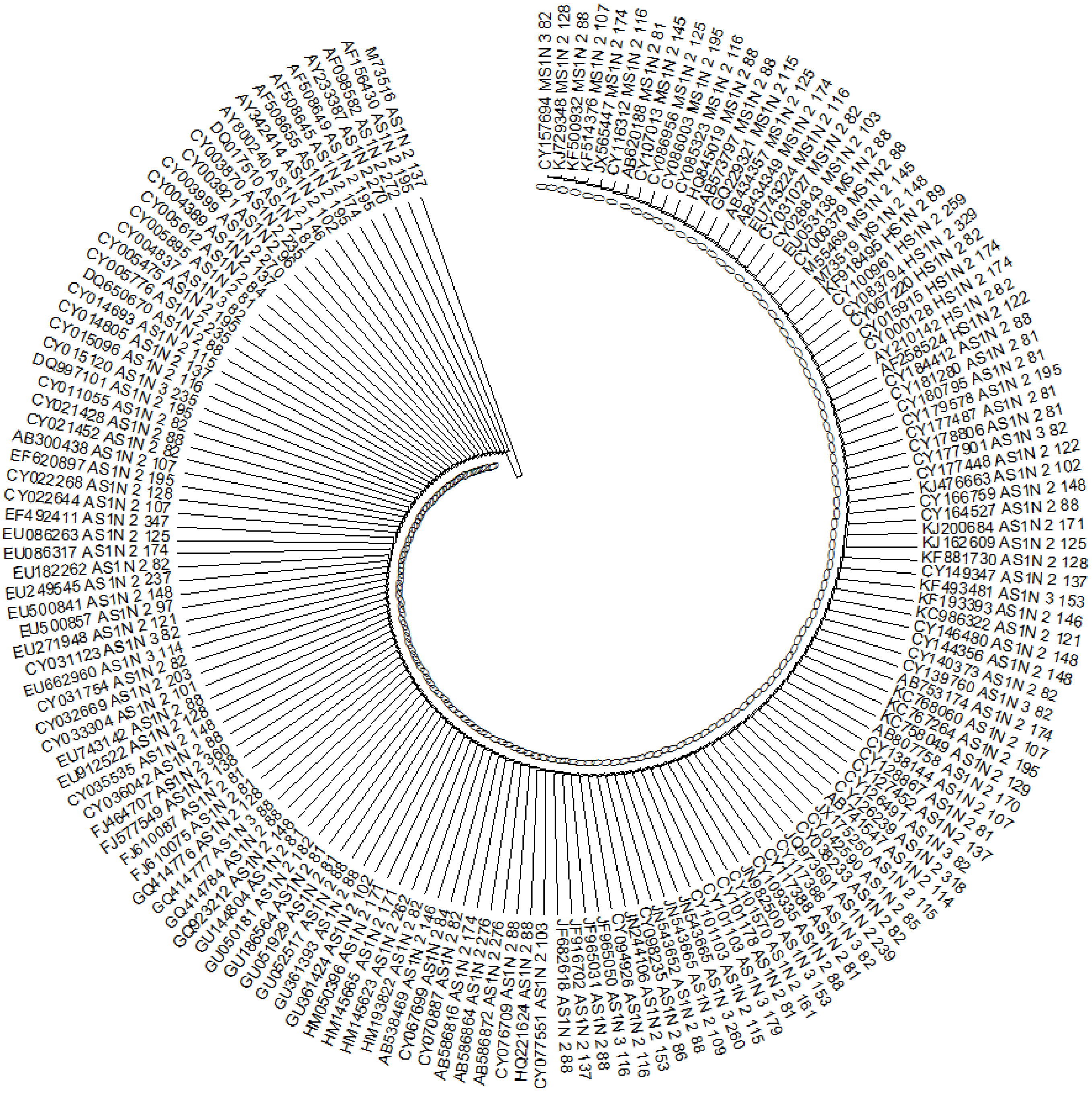

Figure 2. Phylogenetic tree of 163 representative sequences from 163 groups of S1 PCS G2. In total 15,207 protein sequences from S1 PCS G2 of genomic strand of IAV segment 1 were clustered into 163 subgroups by CD-HIT software based on 95% sequence identity. Phylogenetic tree of 163 representative sequences was constructed using the Neighbor joining method with bootstrapping 1000 times by MEGA 6.0.

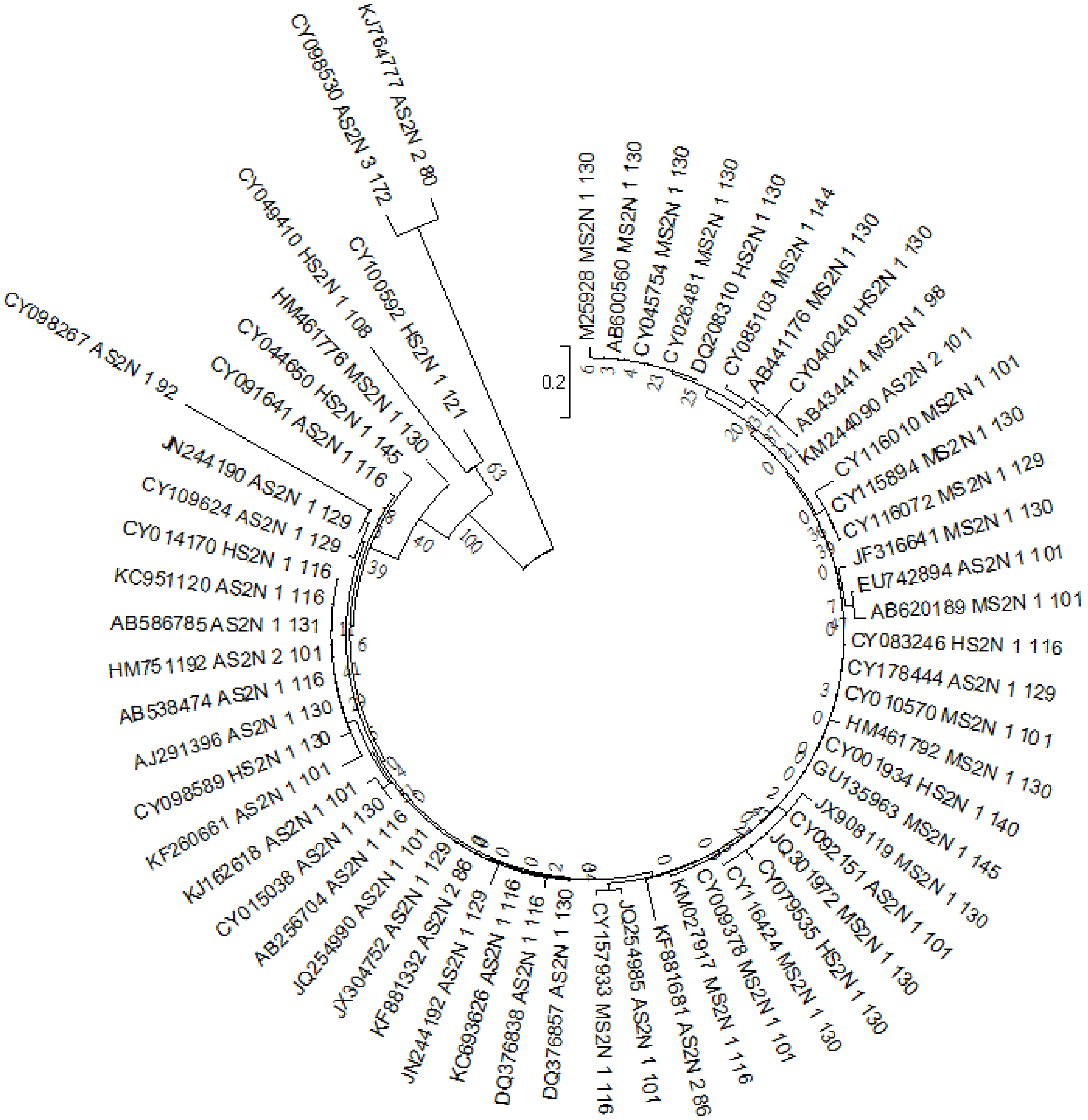

Figure 3. Phylogenetic tree of 60 representative sequences from 60 groups of S2 PCS G1. In total 15,436 protein sequences from S2 PCS G1 of genomic strand of IAV segment 2 were clustered into 60 subgroups by CD-HIT software based on 95% sequence identity. Phylogenetic tree of 60 representative sequences was constructed using the Neighbor joining method with bootstrapping 1000 times by MEGA 6.0.

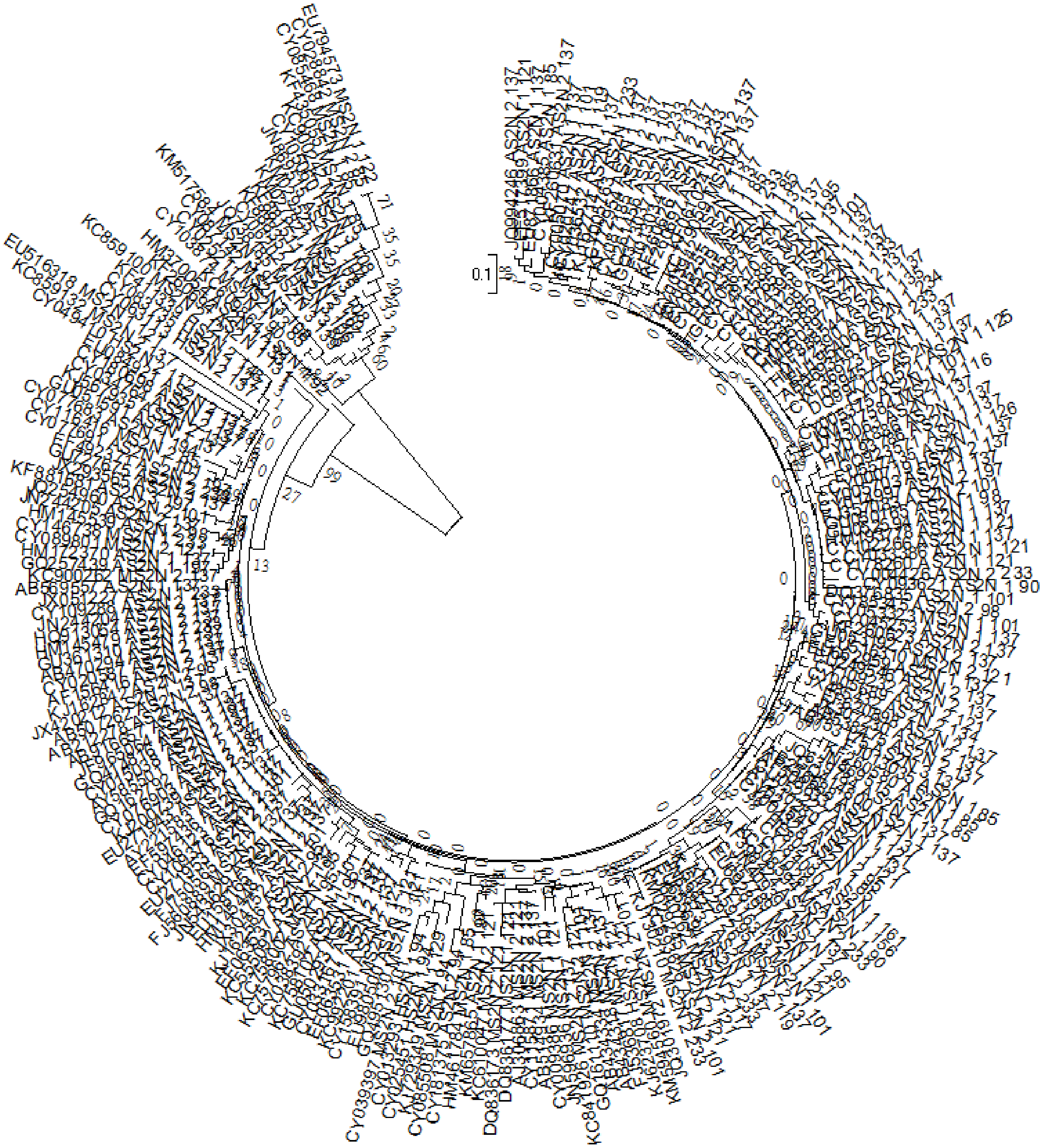

Figure 4. Phylogenetic tree of 225 representative sequences from 225 groups of S2 PCS G2. In total 27,254 protein sequences from S2 PCS G2 of genomic strand of IAV segment 2 were clustered into 225 subgroups by CD-HIT software based on 95% sequence identity. Phylogenetic tree of 225 representative sequences was constructed using the Neighbor joining method with bootstrapping 1000 times by MEGA 6.0.

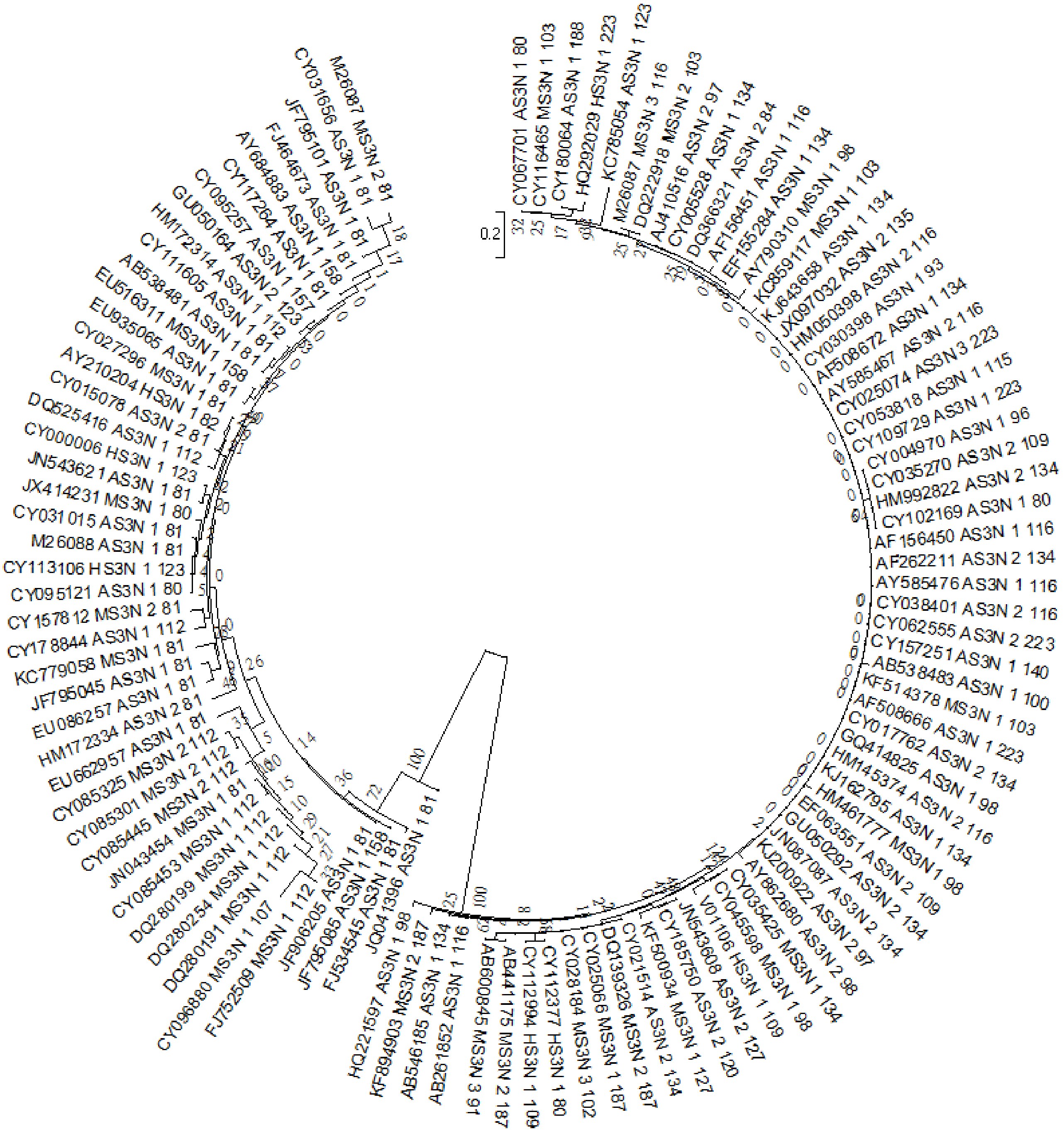

Figure 5. Phylogenetic tree of 109 representative sequences from 109 groups of S3 PCS G1. In total 5,594 protein sequences from S3 PCS G1 of genomic strand of IAV segment 3 were clustered into 109 subgroups by CD-HIT software based on 95% sequence identity. Phylogenetic tree of 109 representative sequences was constructed using the Neighbor joining method with bootstrapping 1000 times by MEGA 6.0.

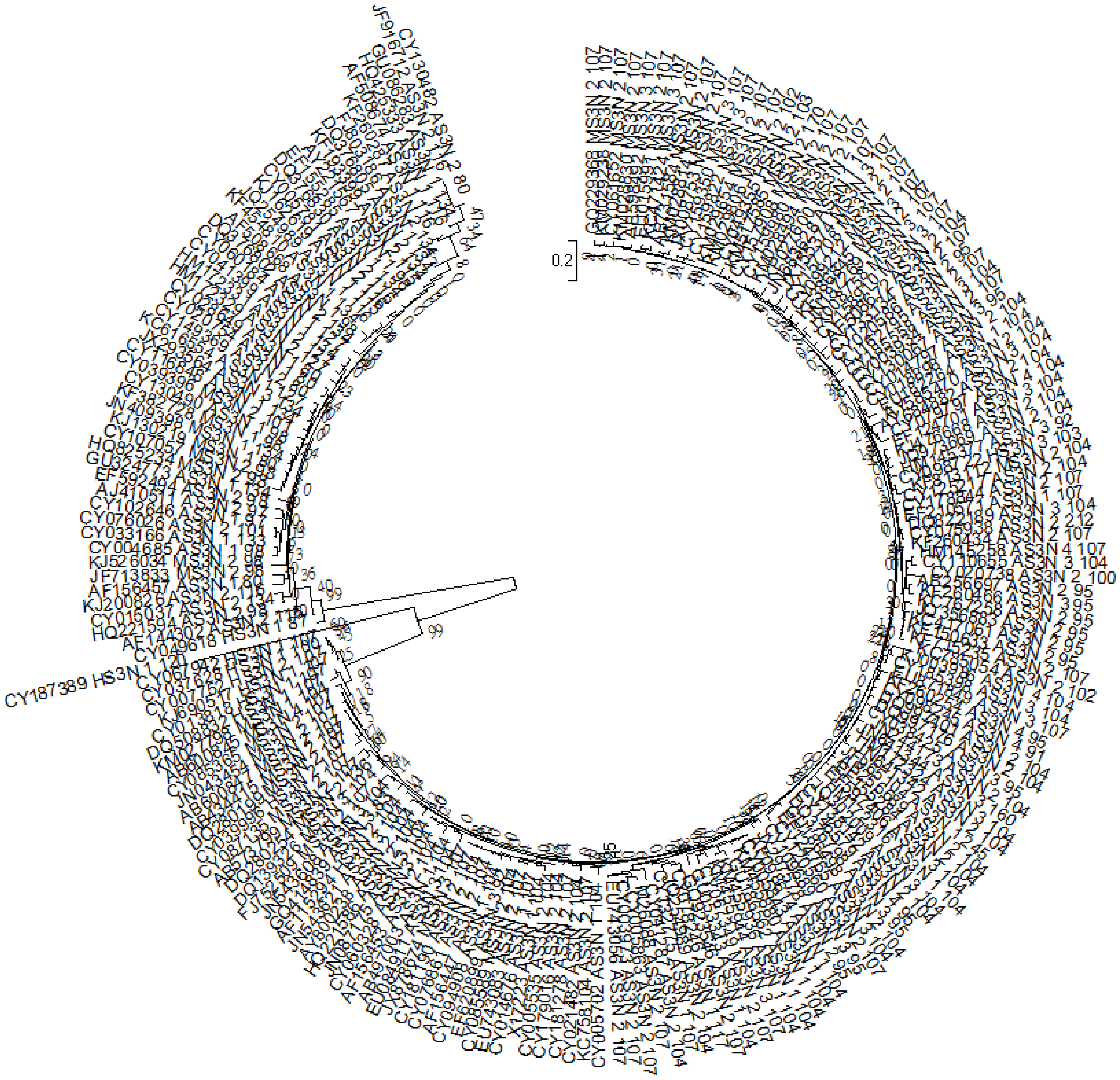

Figure 6. Phylogenetic tree of 204 representative sequences from 204 groups of S3 PCS G2. In total 18,216 protein sequences from S3 PCS G2 of genomic strand of IAV segment 3 were clustered into 204 subgroups by CD-HIT software based on 95% sequence identity. Phylogenetic tree of 204 representative sequences was constructed using the Neighbor joining method with bootstrapping 1000 times by MEGA 6.0.

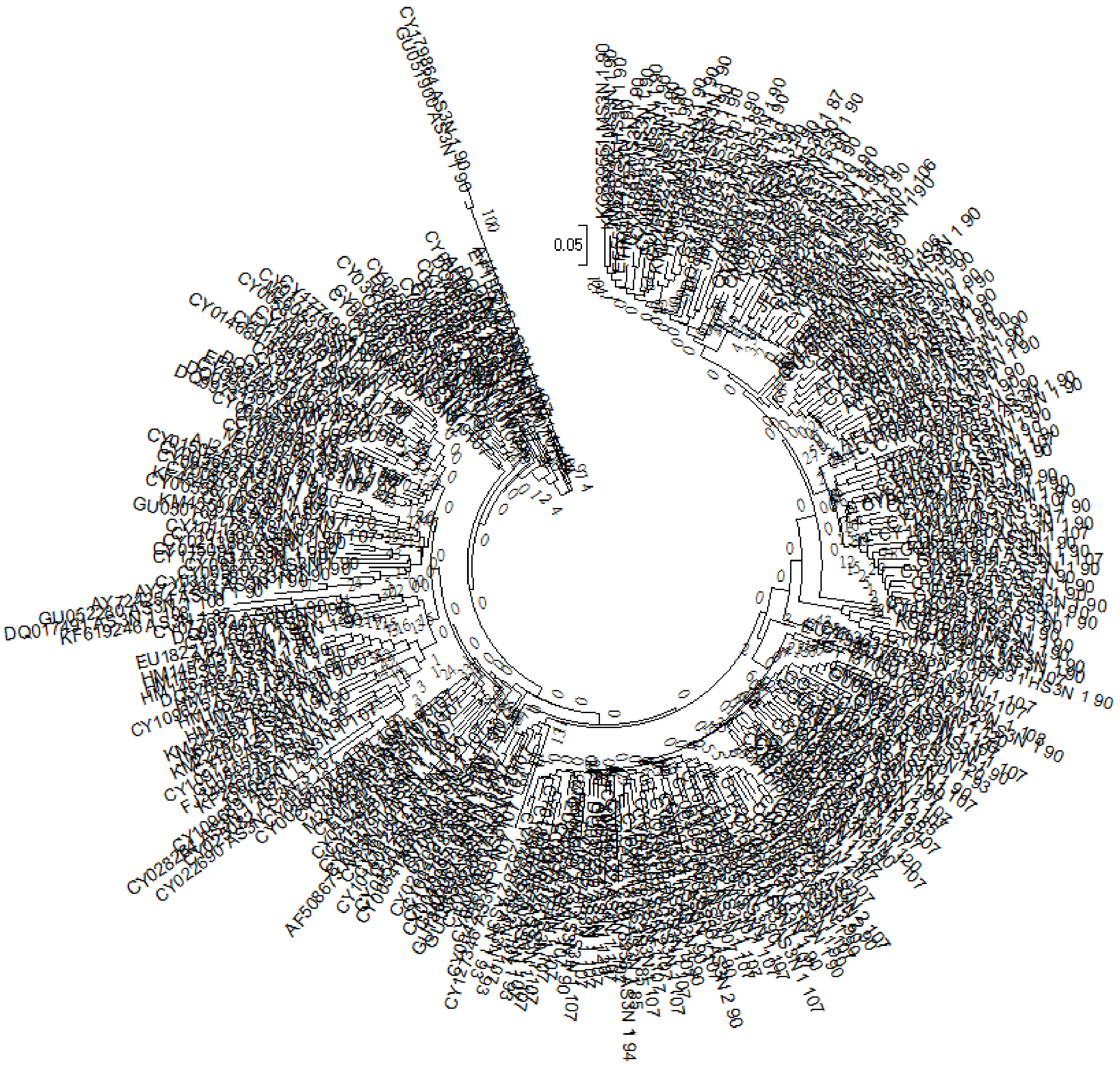

Figure 7. Phylogenetic tree of 319 representative sequences from 319 groups of S3 PCS G3. In total 9,554 protein sequences from S3 PCS G3 of genomic strand of IAV segment 3 were clustered into 319 subgroups by CD-HIT software based on 95% sequence identity. Phylogenetic tree of 319 representative sequences was constructed using the Neighbor joining method with bootstrapping 1000 times by MEGA 6.0.

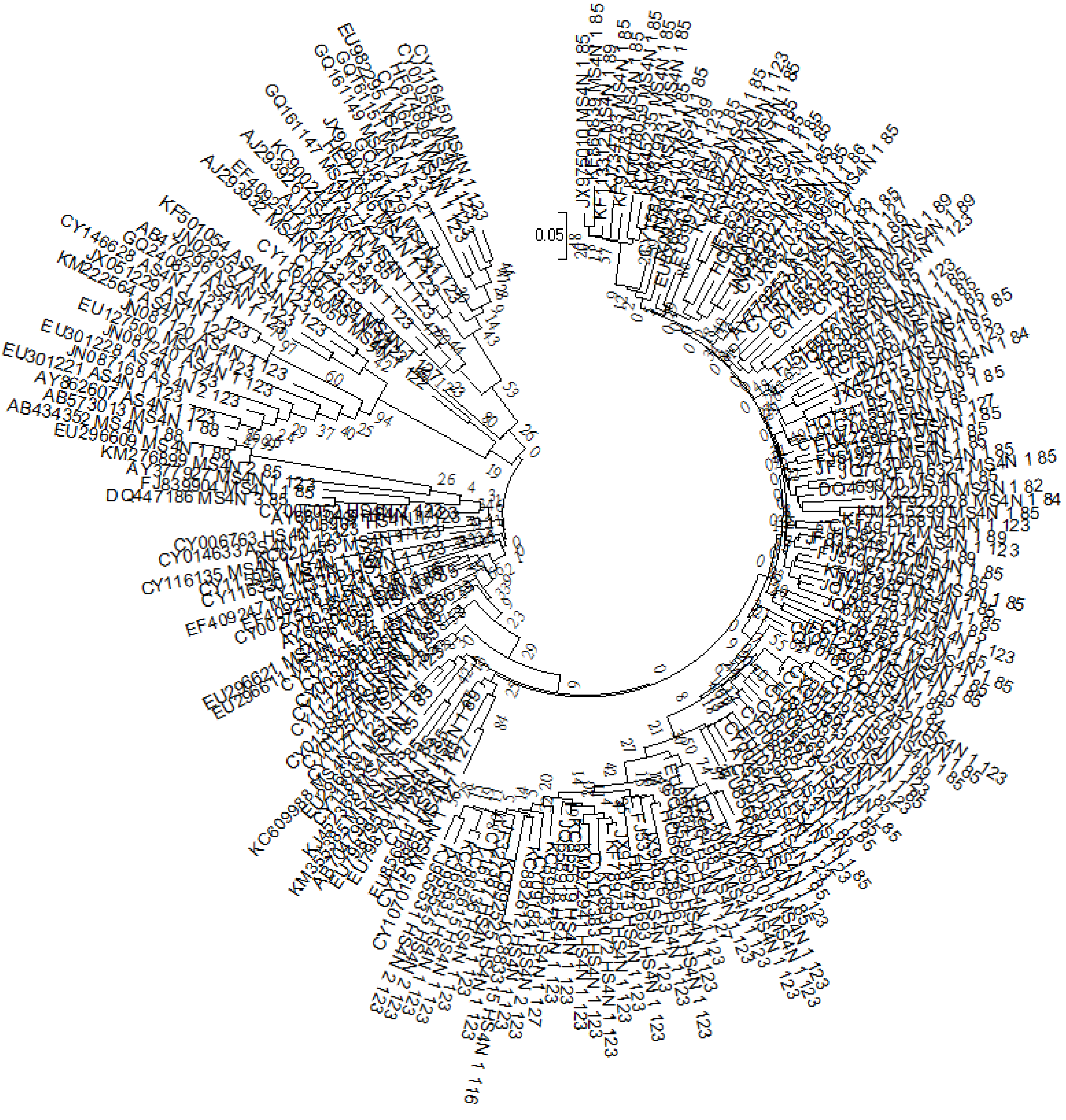

Figure 8. Phylogenetic tree of 200 representative sequences from 200 groups of S4 PCS G1. In total 10,130 protein sequences from S4 PCS G1 of genomic strand of IAV segment 4 were clustered into 200 subgroups by CD-HIT software based on 95% sequence identity. Phylogenetic tree of 200 representative sequences was constructed using the Neighbor joining method with bootstrapping 1000 times by MEGA 6.0.

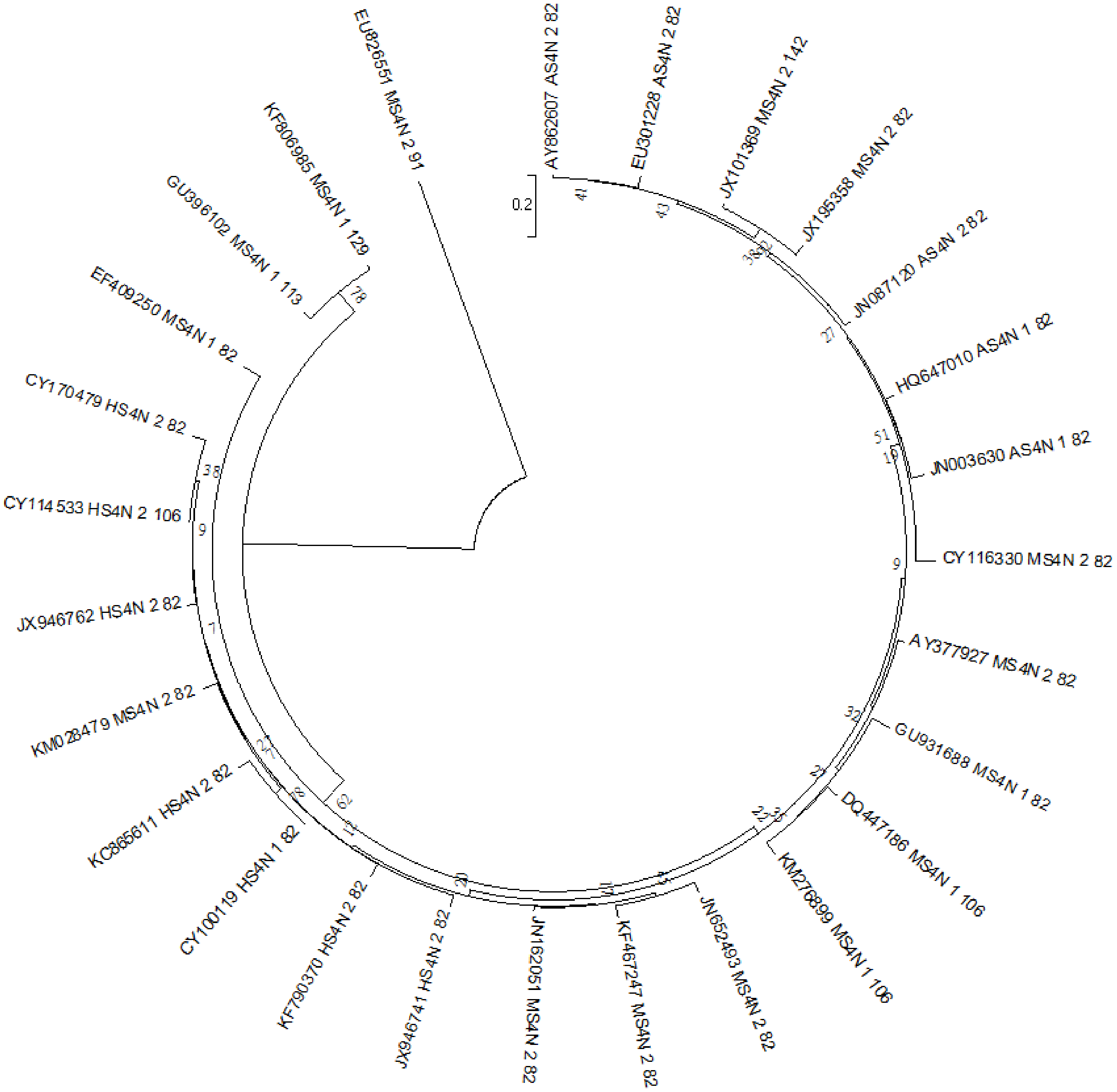

Figure 9. Phylogenetic tree of 27 representative sequences from 27 groups of S4 PCS G2. In total 6,098 protein sequences from S4 PCS G2 of genomic strand of IAV segment 4 were clustered into 27 subgroups by CD-HIT software based on 95% sequence identity. Phylogenetic tree of 27 representative sequences was constructed using the Neighbor joining method with bootstrapping 1000 times by MEGA 6.0.

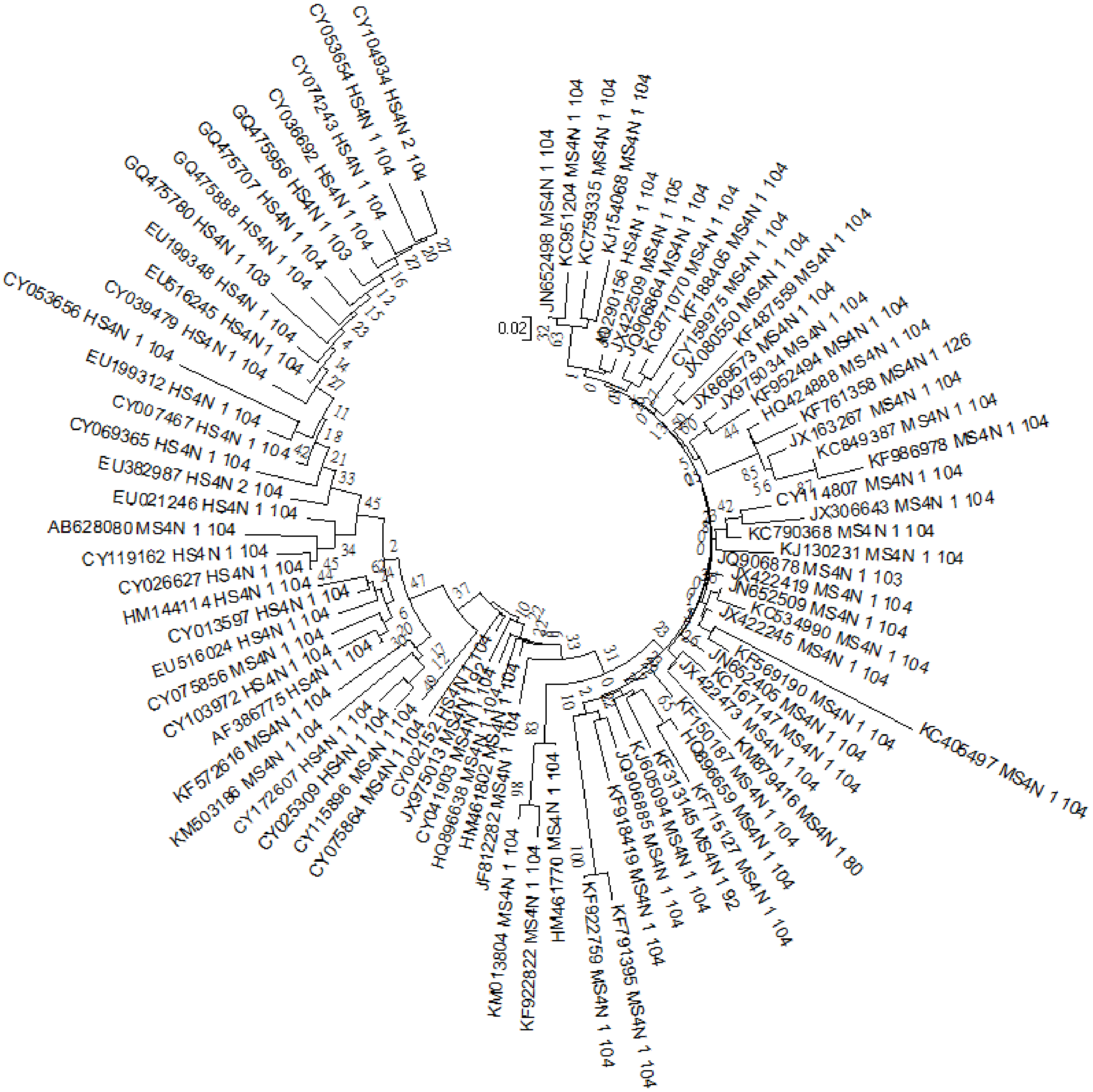

Figure 10. Phylogenetic tree of 85 representative sequences from 85 groups of S4 PCS G3. In total 2,632 protein sequences from S4 PCS G3 of genomic strand of IAV segment 4 were clustered into 85 subgroups by CD-HIT software based on 95% sequence identity. Phylogenetic tree of 85 representative sequences was constructed using the Neighbor joining method with bootstrapping 1000 times by MEGA 6.0.

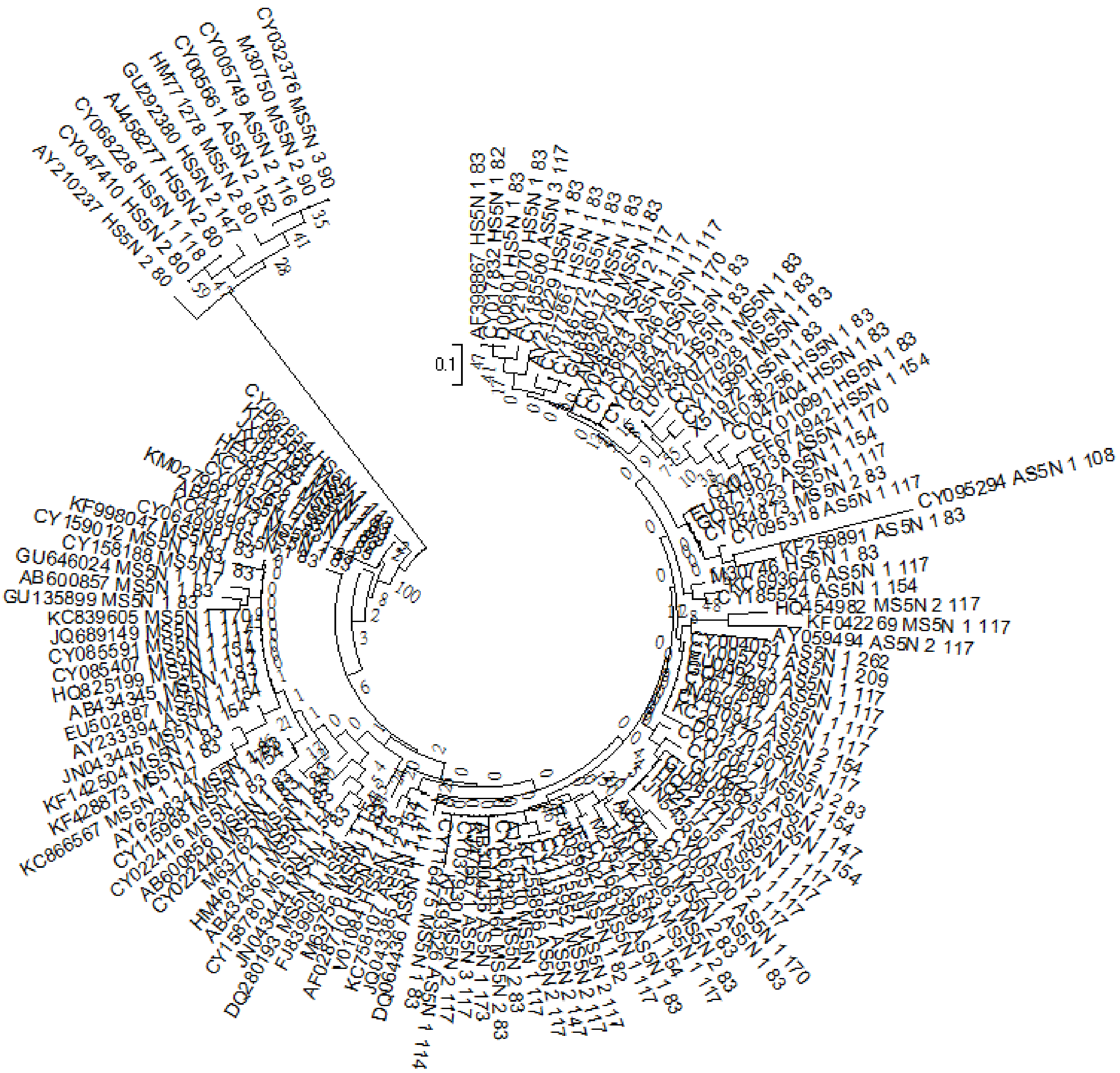

Figure 11. Phylogenetic tree of 131 representative sequences from 131 groups of S5 PCS G1. In total 19,571 protein sequences from S5 PCS G1 of genomic strand of IAV segment 5 were clustered into 131 subgroups by CD-HIT software based on 95% sequence identity. Phylogenetic tree of 131 representative sequences was constructed using the Neighbor joining method with bootstrapping 1000 times by MEGA 6.0.

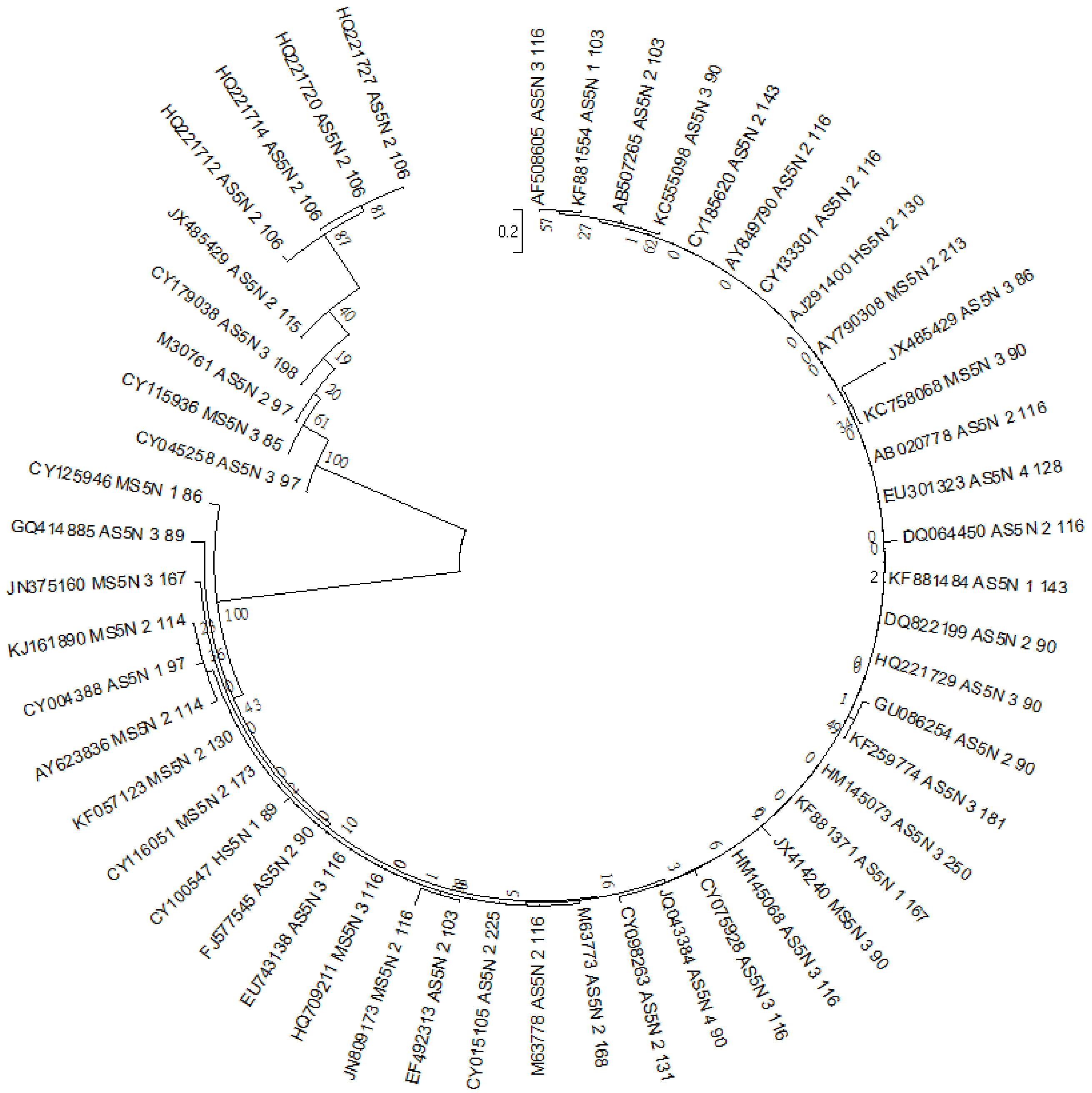

Figure 12. Phylogenetic tree of 52 representative sequences from 52 groups of S5 PCS G2. In total 4,375 protein sequences from S5 PCS G2 of genomic strand of IAV segment 5 were clustered into 52 subgroups by CD-HIT software based on 95% sequence identity. Phylogenetic tree of 52 representative sequences was constructed using the Neighbor joining method with bootstrapping 1000 times by MEGA 6.0.

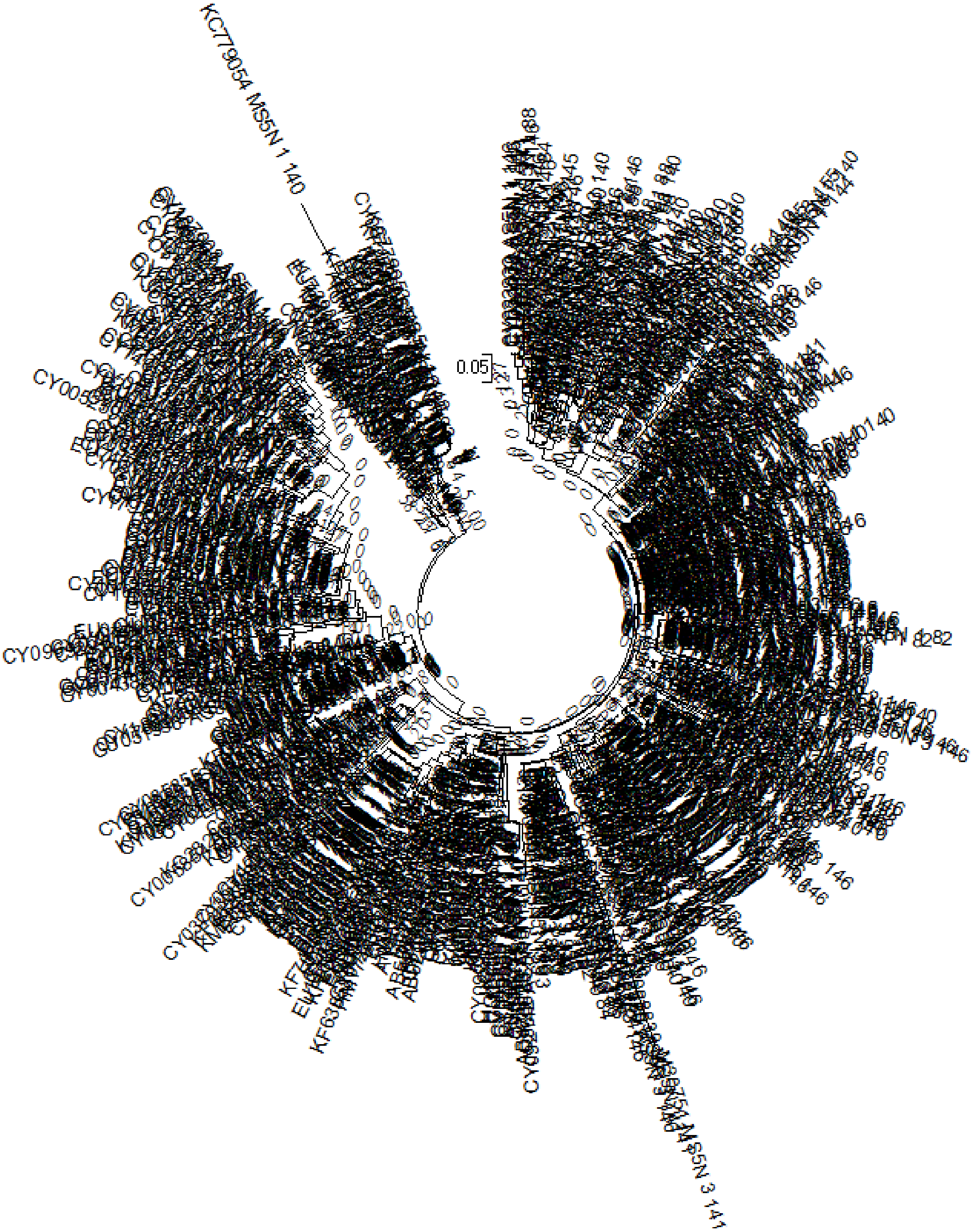

Figure 13. Phylogenetic tree of 604 representative sequences from 604 groups of S5 PCS G3. In total 7,169 protein sequences from S5 PCS G3 of genomic strand of IAV segment 5 were clustered into 604 subgroups by CD-HIT software based on 95% sequence identity. Phylogenetic tree of 604 representative sequences was constructed using the Neighbor joining method with bootstrapping 1000 times by MEGA 6.0.

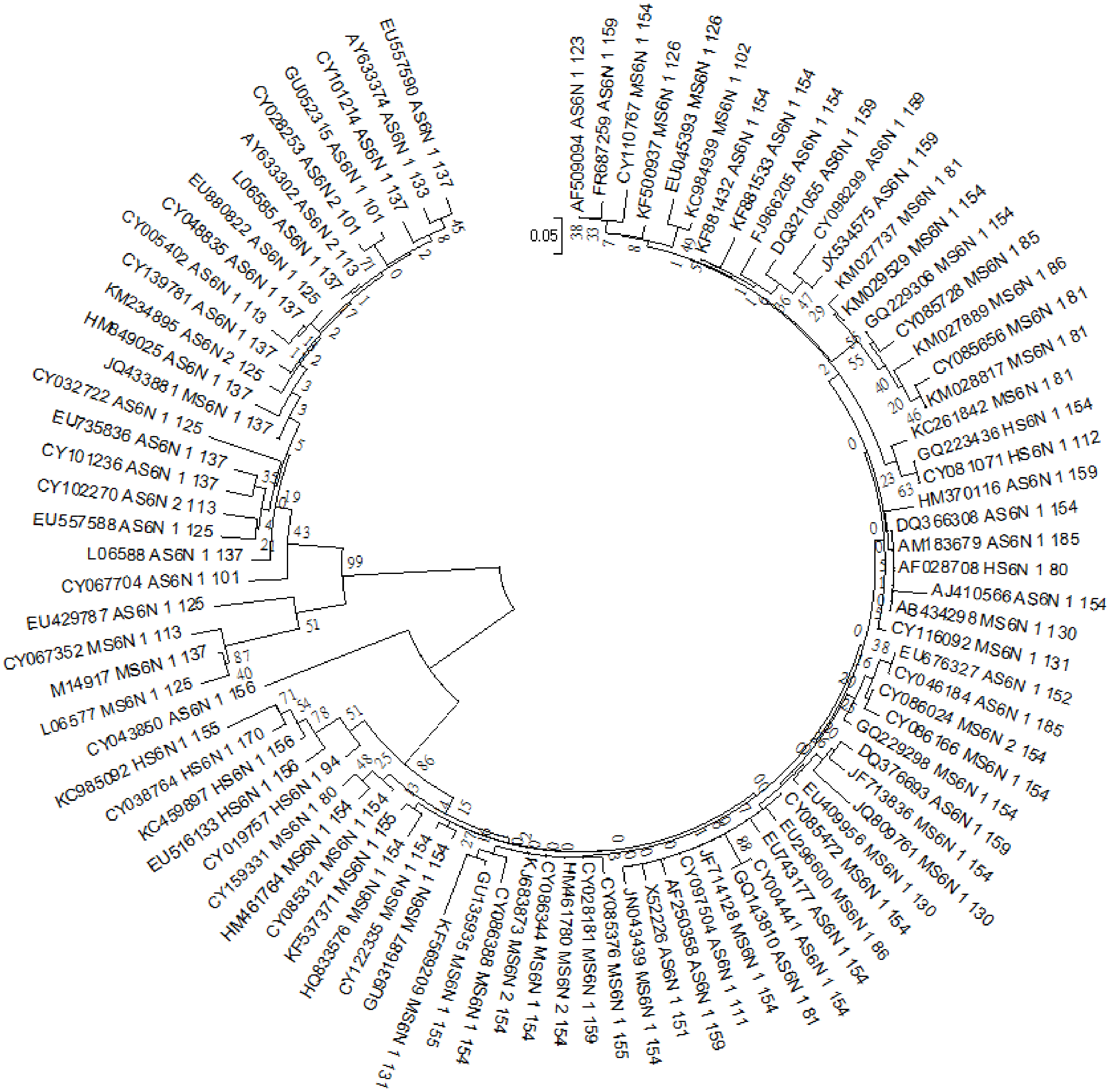

Figure 14. Phylogenetic tree of 94 representative sequences from 94 groups of S6 PCS G1. In total 7,319 protein sequences from S6 PCS G1 of genomic strand of IAV segment 6 were clustered into 94 subgroups by CD-HIT software based on 95% sequence identity. Phylogenetic tree of 94 representative sequences was constructed using the Neighbor joining method with bootstrapping 1000 times by MEGA 6.0.

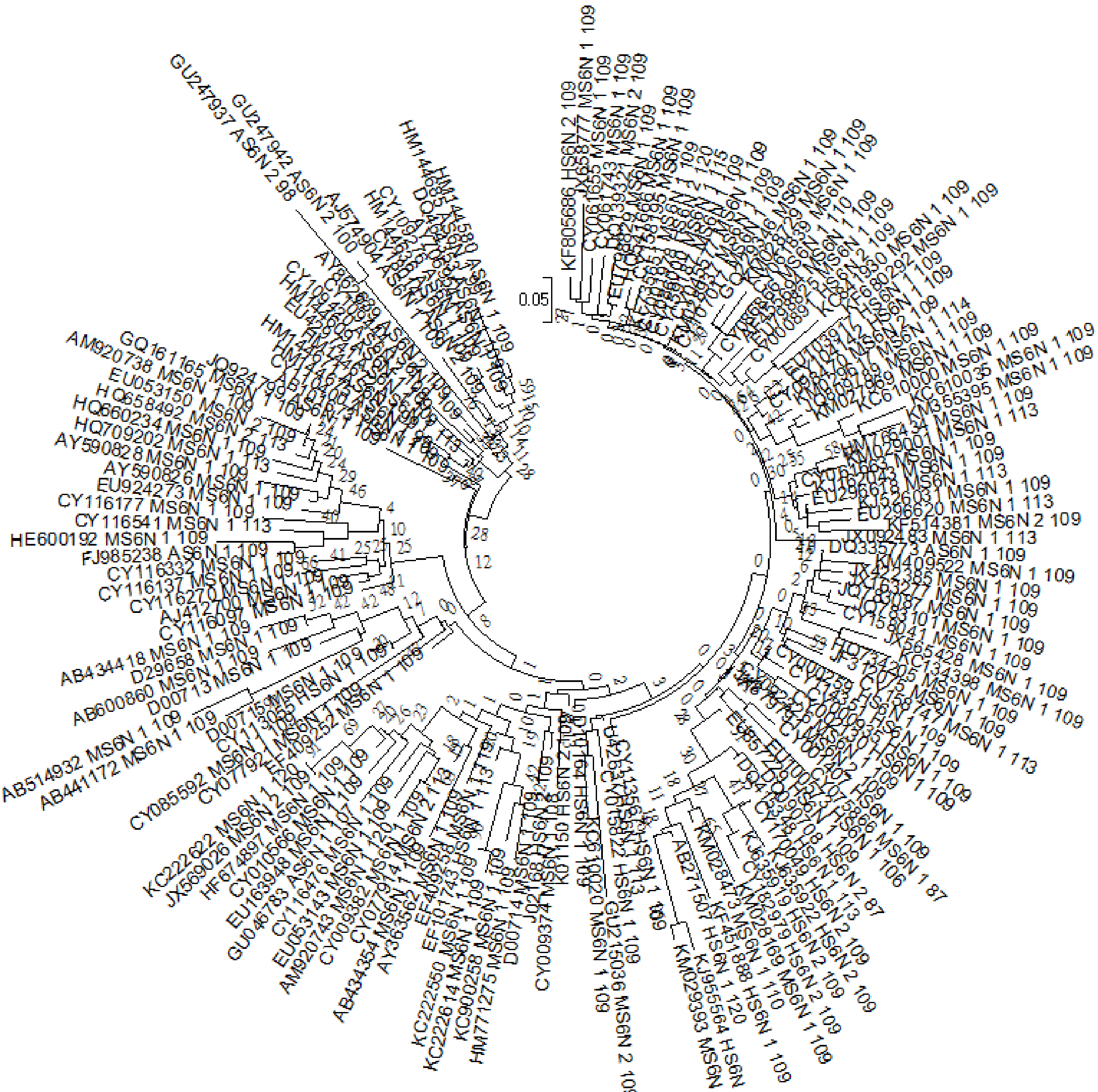

Figure 15. Phylogenetic tree of 155 representative sequences from 155 groups of S6 PCS G2. In total 9,841 protein sequences from S6 PCS G2 of genomic strand of IAV segment 6 were clustered into 155 subgroups by CD-HIT software based on 95% sequence identity. Phylogenetic tree of 155 representative sequences was constructed using the Neighbor joining method with bootstrapping 1000 times by MEGA 6.0.

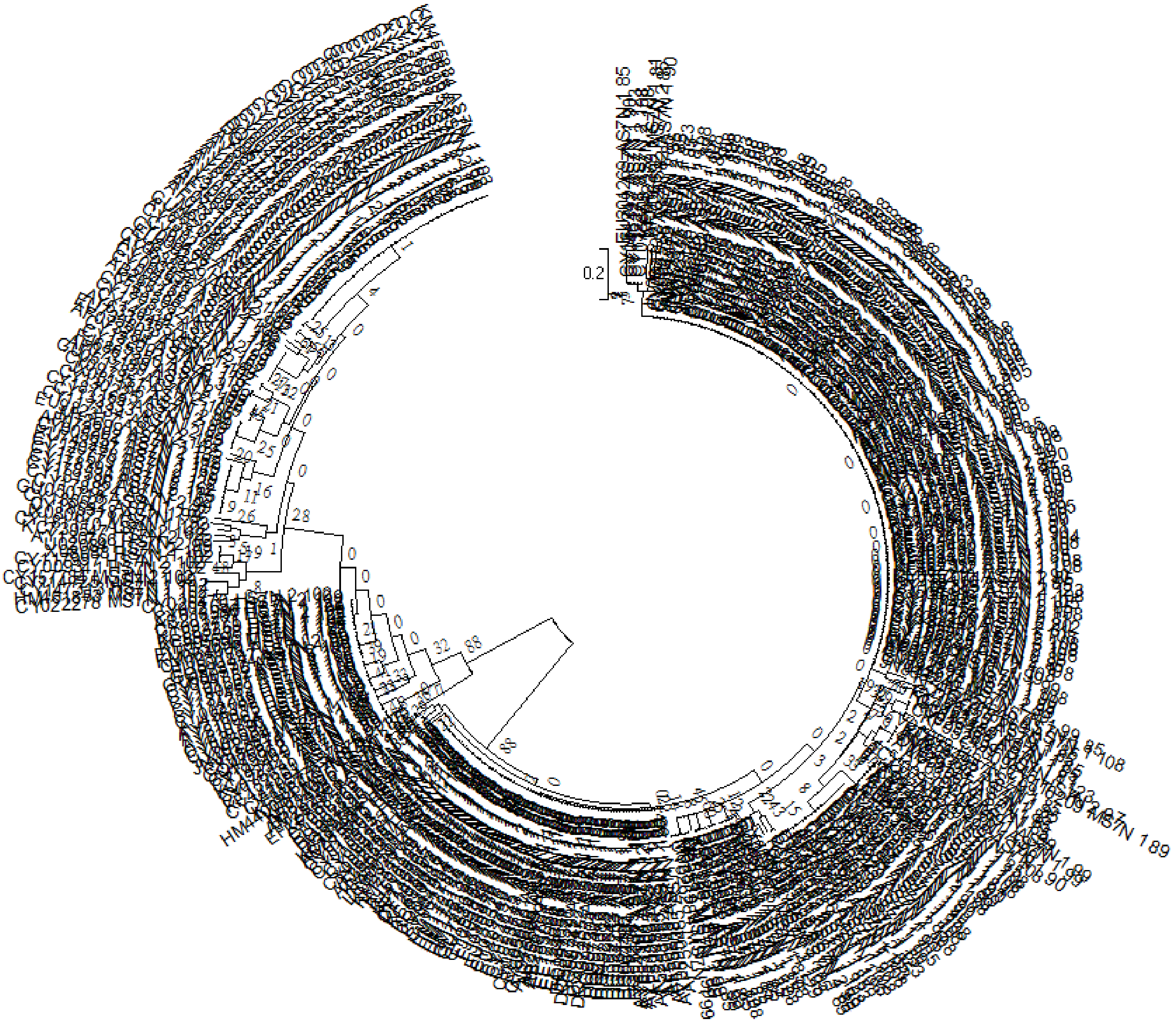

Figure 16. Phylogenetic tree of 447 representative sequences from 447 groups of S7 PCS G1. In total 9,580 protein sequences from S7 PCS G1 of genomic strand of IAV segment 7 were clustered into 447 subgroups by CD-HIT software based on 95% sequence identity. Phylogenetic tree of 447 representative sequences was constructed using the Neighbor joining method with bootstrapping 1000 times by MEGA 6.0.

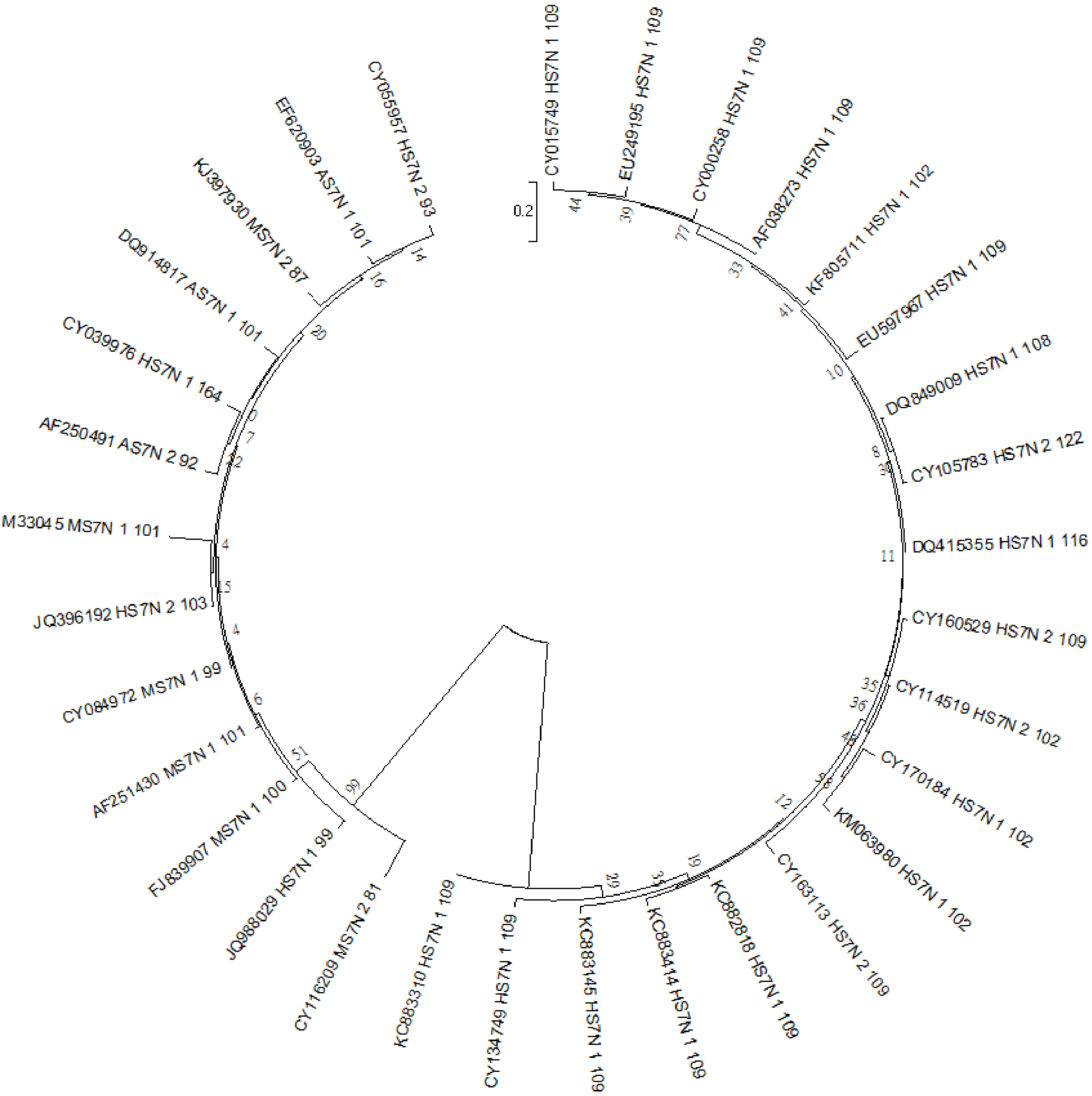

Figure 17. Phylogenetic tree of 32 representative sequences from 32 groups of S7 PCS G2. In total 14,945 protein sequences from S7 PCS G2 of genomic strand of IAV segment 7 were clustered into 32 subgroups by CD-HIT software based on 95% sequence identity. Phylogenetic tree of 32 representative sequences was constructed using the Neighbor joining method with bootstrapping 1000 times by MEGA 6.0.

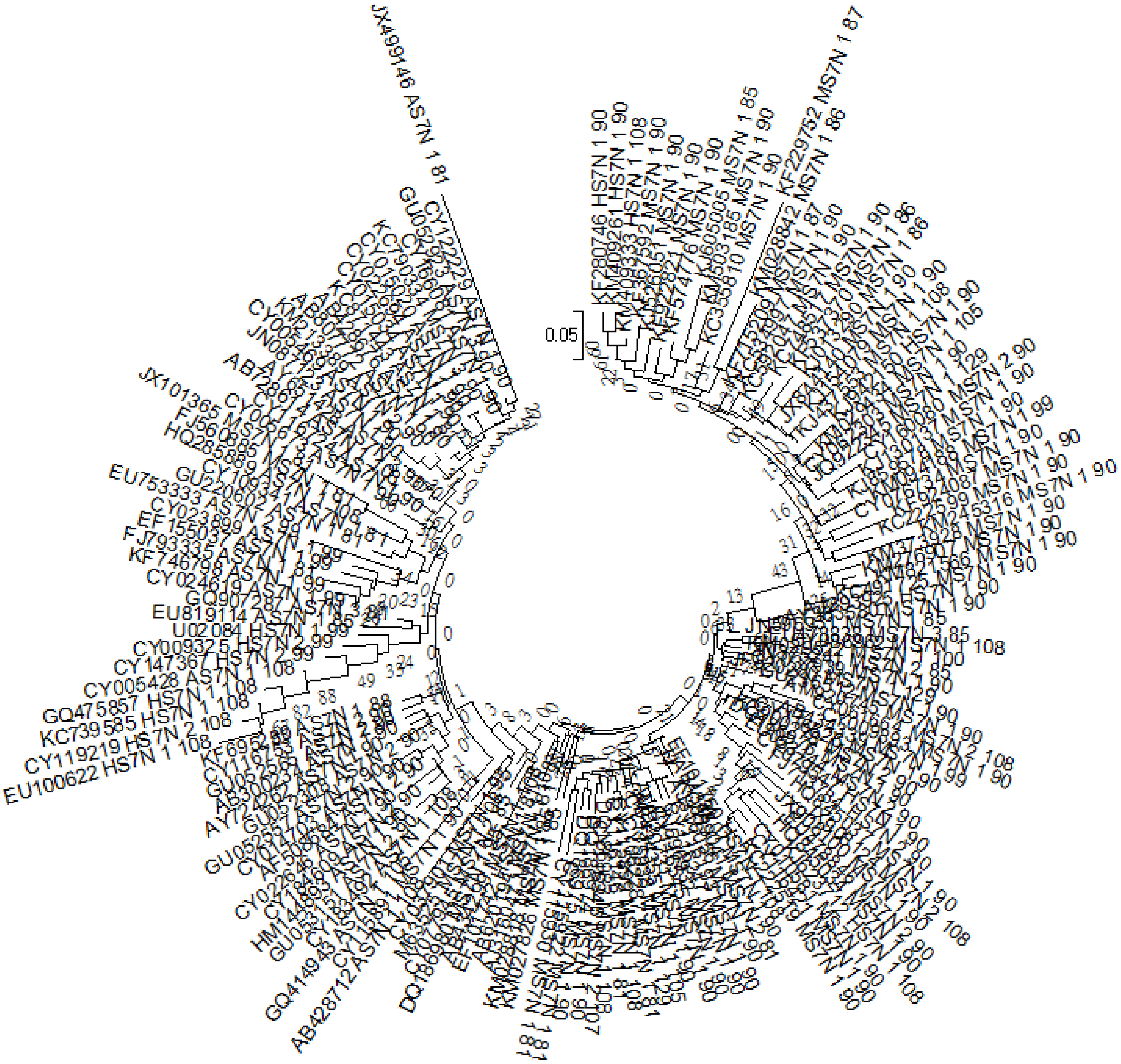

Figure 18. Phylogenetic tree of 152 representative sequences from 152 groups of S7 PCS G3. In total 10,398 protein sequences from S7 PCS G3 of genomic strand of IAV segment 7 were clustered into 152 subgroups by CD-HIT software based on 95% sequence identity. Phylogenetic tree of 152 representative sequences was constructed using the Neighbor joining method with bootstrapping 1000 times by MEGA 6.0.

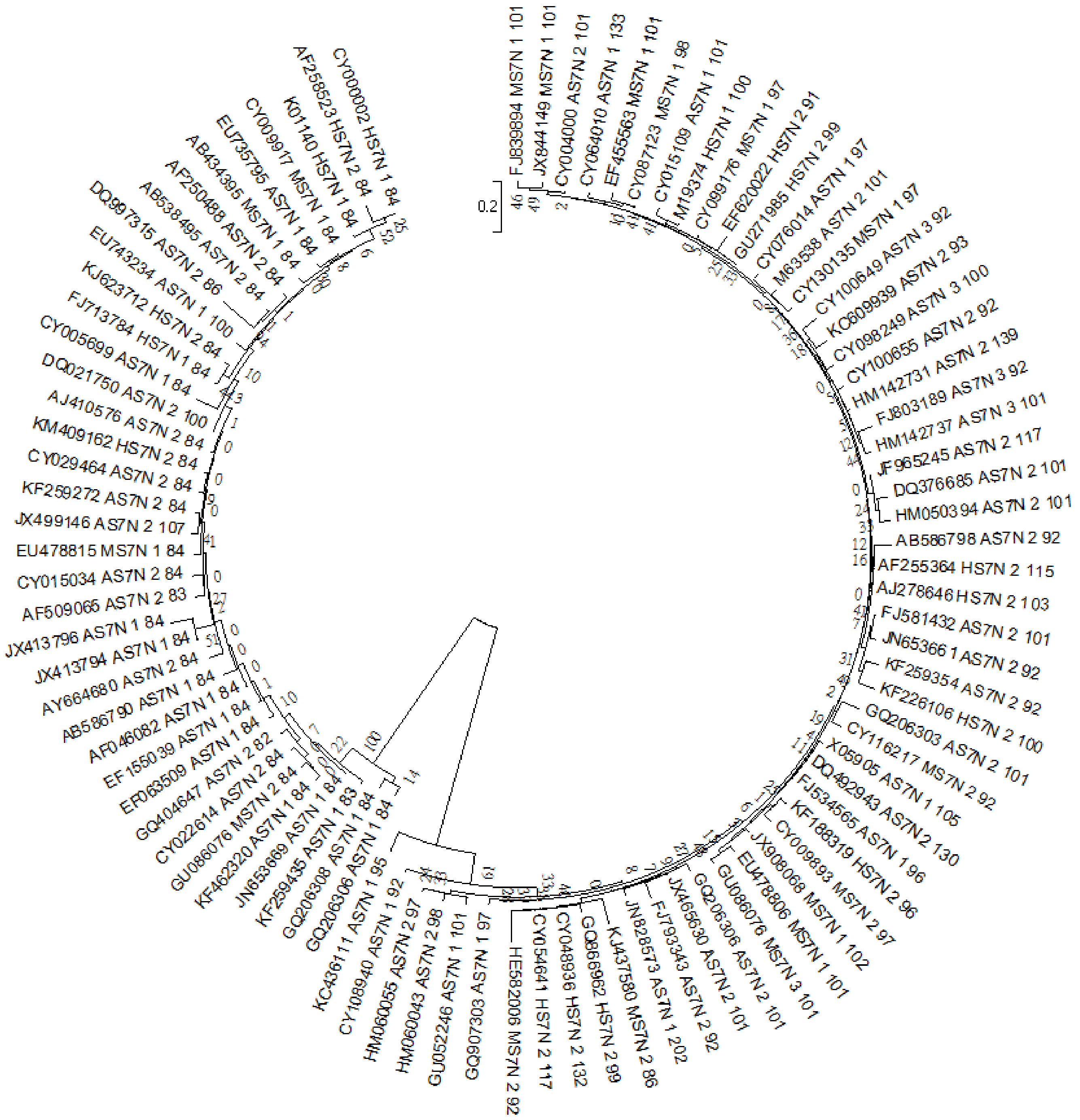

Figure 19. Phylogenetic tree of 93 representative sequences from 93 groups of S7 PCS G4. In total 23,751 protein sequences from S7 PCS G4 of genomic strand of IAV segment 7 were clustered into 93 subgroups by CD-HIT software based on 95% sequence identity. Phylogenetic tree of 93 representative sequences was constructed using the Neighbor joining method with bootstrapping 1000 times by MEGA 6.0.

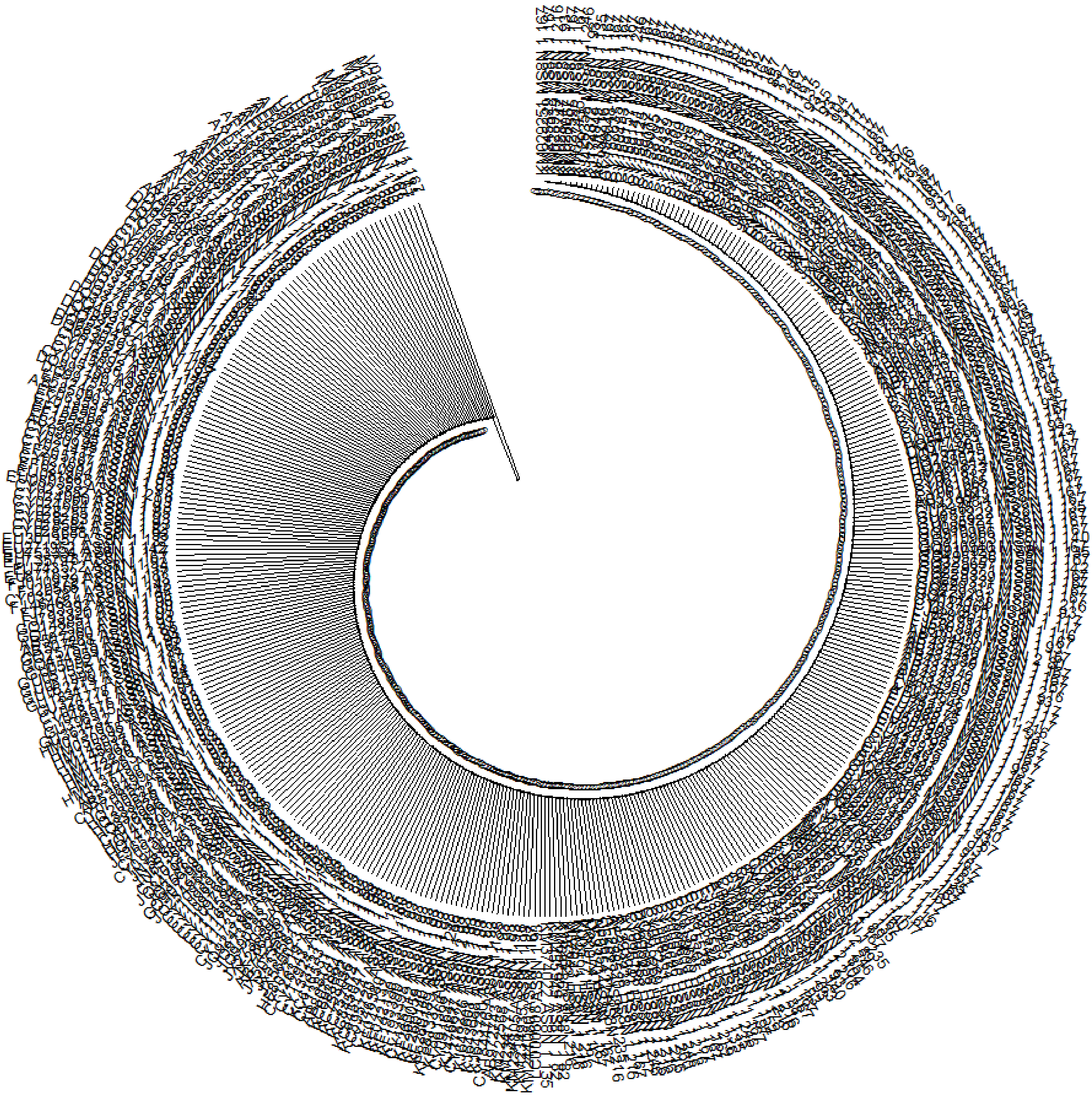

Figure 20. Phylogenetic tree of 413 representative sequences from 413 groups of S8 PCS G1. In total 20,389 protein sequences from S8 PCS G1 of genomic strand of IAV segment 8 were clustered into 413 subgroups by CD-HIT software based on 95% sequence identity. Phylogenetic tree of 413 representative sequences was constructed using the Neighbor joining method with bootstrapping 1000 times by MEGA 6.0.
