# Supplementary material for: Uncovering the Potential Pan Proteomes Encoded by Genomic Strand RNAs of Influenza A Viruses
Source: PLoS One. 2016 Jan 13;11(1):e0146936. doi: 10.1371/journal.pone.0146936 (PMC4711952; doi:10.1371/journal.pone.0146936)

Influenza A virus (A/Brevig Mission/1/1918(H1N1))

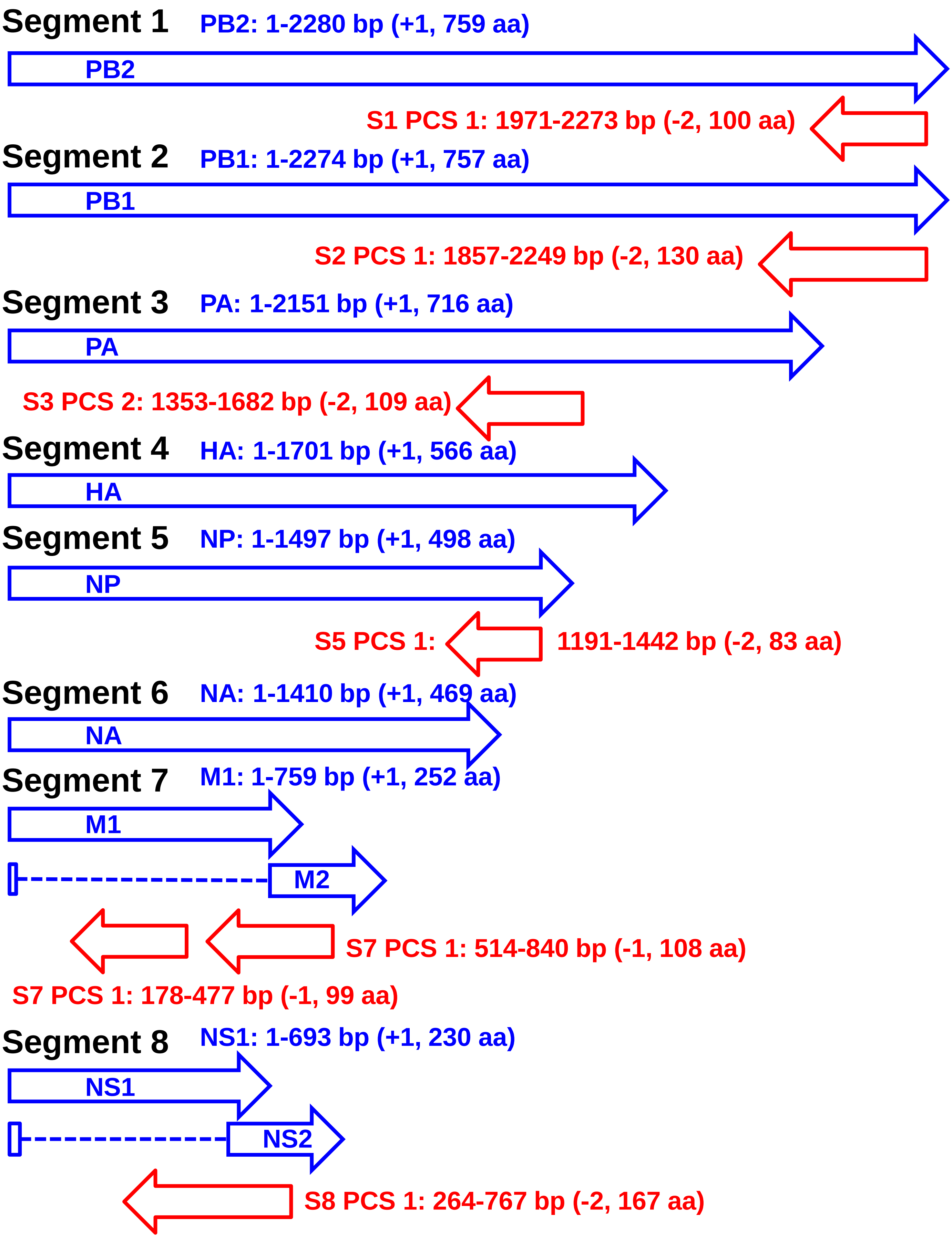

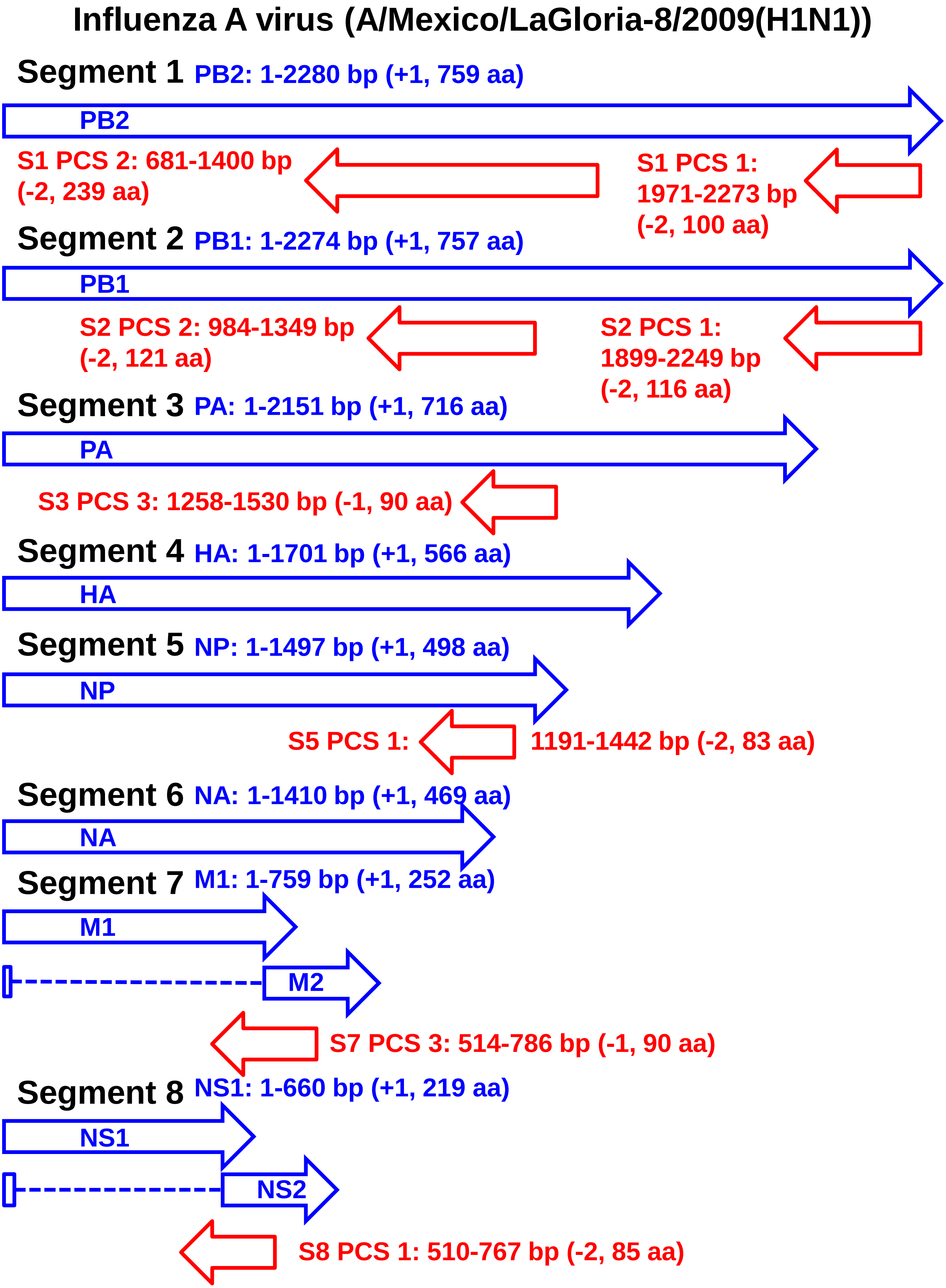
Influenza A virus (A/Mexico/LaGloria-8/2009(H1N1))
Segment 1 PB2: 1-2280 bp (+1, 759 aa)
PB2
S1 PCS 2: 681-1400 bp (-2, 239 aa)
S1 PCS 1: 1971-2273 bp (-2, 100 aa)
Segment 2 PB1: 1-2274 bp (+1, 757 aa)
PB1
S2 PCS 2: 984-1349 bp (-2, 121 aa)
S2 PCS 1: 1899-2249 bp (-2, 116 aa)
Segment 3 PA: 1-2151 bp (+1, 716 aa)
PA
S3 PCS 3: 1258-1530 bp (-1, 90 aa)
Segment 4 HA: 1-1701 bp (+1, 566 aa)
HA
Segment 5 NP: 1-1497 bp (+1, 498 aa)
NP
S5 PCS 1:
1191-1442 bp (-2, 83 aa)
Segment 6 NA: 1-1410 bp (+1, 469 aa)
NA
Segment 7 M1: 1-759 bp (+1, 252 aa)
M1
M2
S7 PCS 3: 514-786 bp (-1, 90 aa)
Segment 8 NS1: 1-660 bp (+1, 219 aa)
NS1
NS2
S8 PCS 1: 510-767 bp (-2, 85 aa)

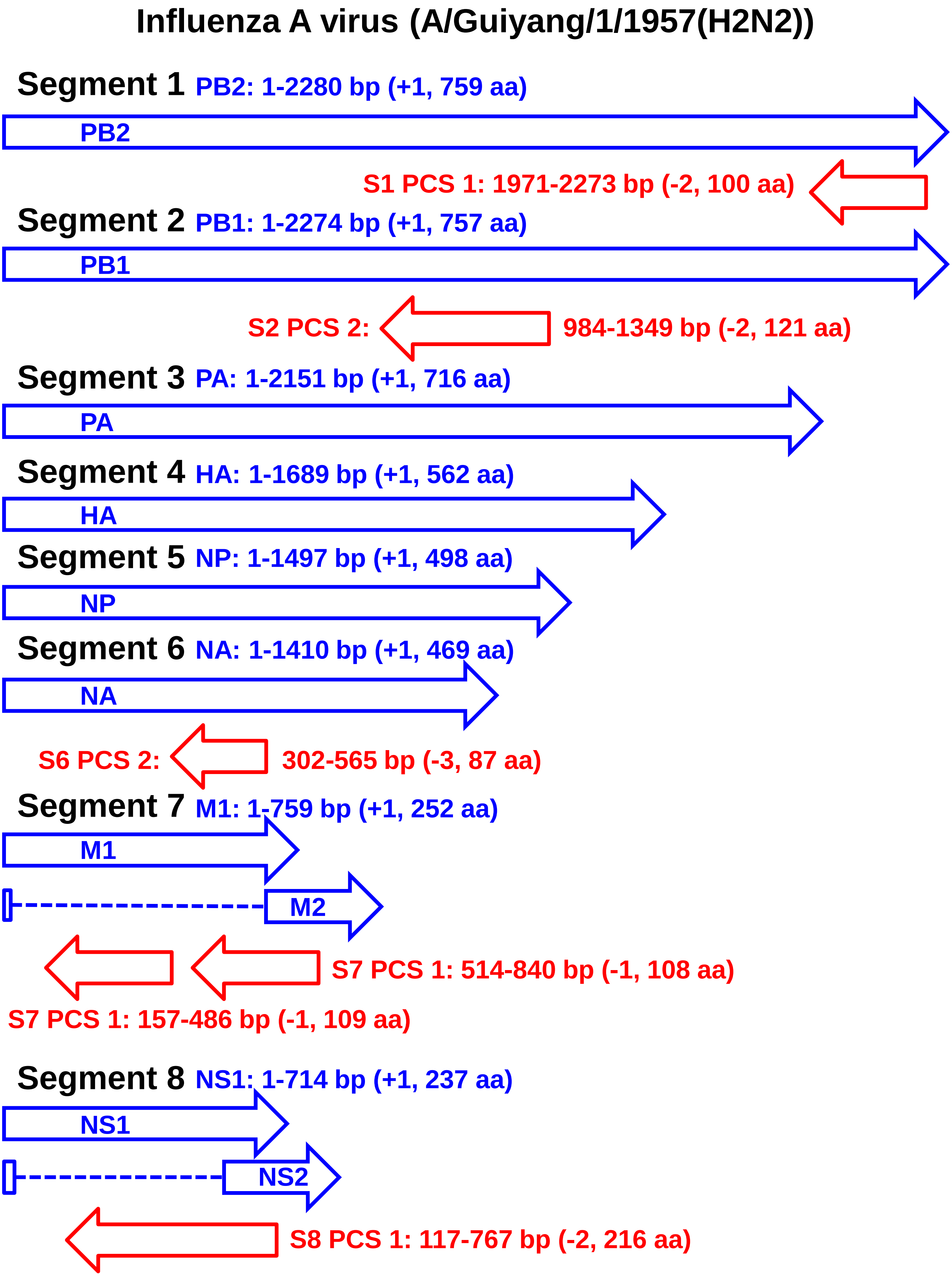
Influenza A virus (A/Guiyang/1/1957(H2N2))
Segment 1 PB2: 1-2280 bp (+1, 759 aa)
PB2
S1 PCS 1: 1971-2273 bp (-2, 100 aa)
Segment 2 PB1: 1-2274 bp (+1, 757 aa)
PB1
S2 PCS 2:
984-1349 bp (-2, 121 aa)
Segment 3 PA: 1-2151 bp (+1, 716 aa)
PA
Segment 4 HA: 1-1689 bp (+1, 562 aa)
HA
Segment 5 NP: 1-1497 bp (+1, 498 aa)
NP
Segment 6 NA: 1-1410 bp (+1, 469 aa)
NA
S6 PCS 2:
302-565 bp (-3, 87 aa)
Segment 7 M1: 1-759 bp (+1, 252 aa)
M1
M2
S7 PCS 1: 514-840 bp (-1, 108 aa)
S7 PCS 1: 157-486 bp (-1, 109 aa)
Segment 8 NS1: 1-714 bp (+1, 237 aa)
NS1
NS2
S8 PCS 1: 117-767 bp (-2, 216 aa)

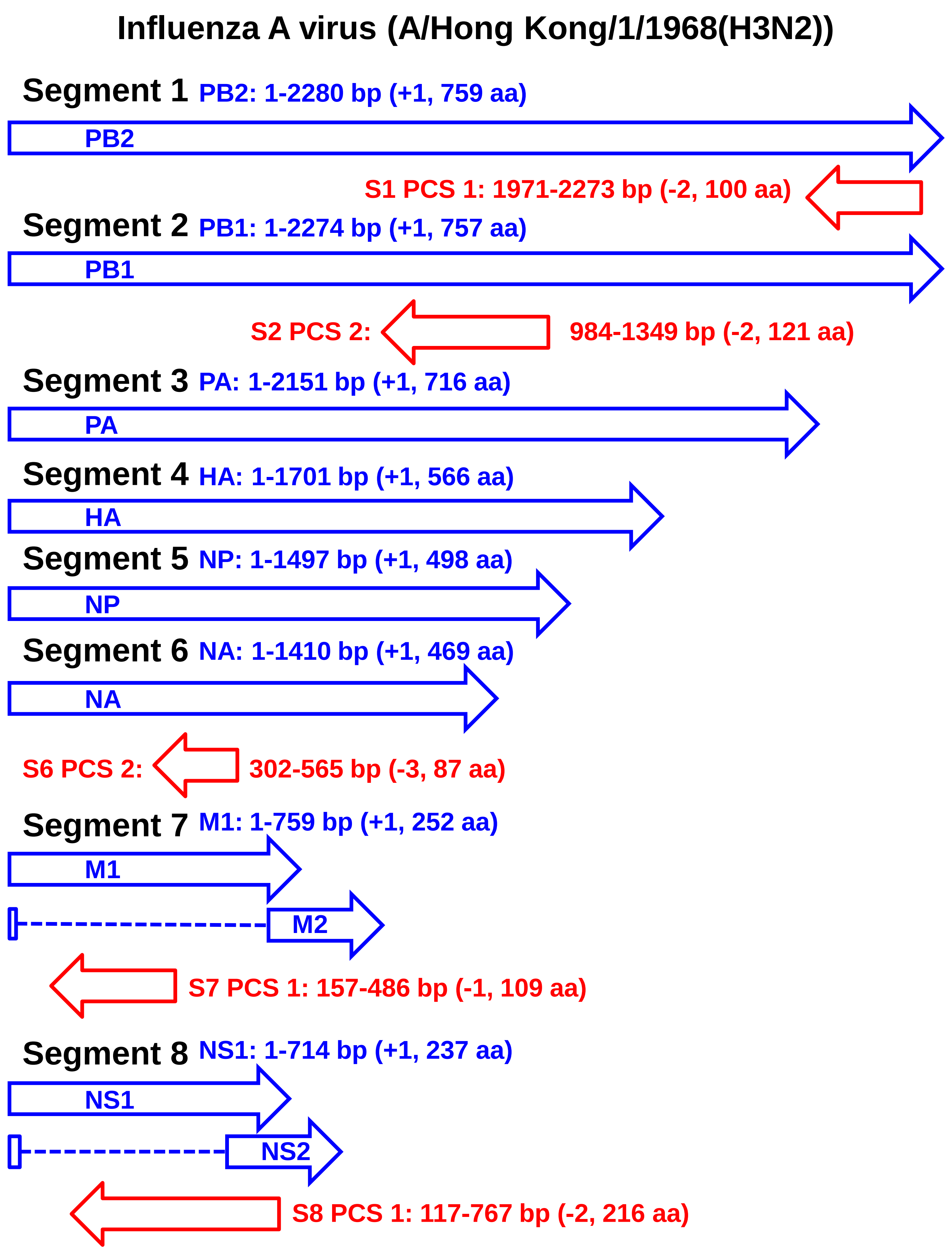
Influenza A virus (A/Hong Kong/1/1968(H3N2))
Segment 1 PB2: 1-2280 bp (+1, 759 aa)
PB2
S1 PCS 1: 1971-2273 bp (-2, 100 aa)
Segment 2 PB1: 1-2274 bp (+1, 757 aa)
PB1
S2 PCS 2:
984-1349 bp (-2, 121 aa)
Segment 3 PA: 1-2151 bp (+1, 716 aa)
PA
Segment 4 HA: 1-1701 bp (+1, 566 aa)
HA
Segment 5 NP: 1-1497 bp (+1, 498 aa)
NP
Segment 6 NA: 1-1410 bp (+1, 469 aa)
NA
S6 PCS 2:
302-565 bp (-3, 87 aa)
Segment 7 M1: 1-759 bp (+1, 252 aa)
M1
M2
S7 PCS 1: 157-486 bp (-1, 109 aa)
Segment 8 NS1: 1-714 bp (+1, 237 aa)
NS1
NS2
S8 PCS 1: 117-767 bp (-2, 216 aa)

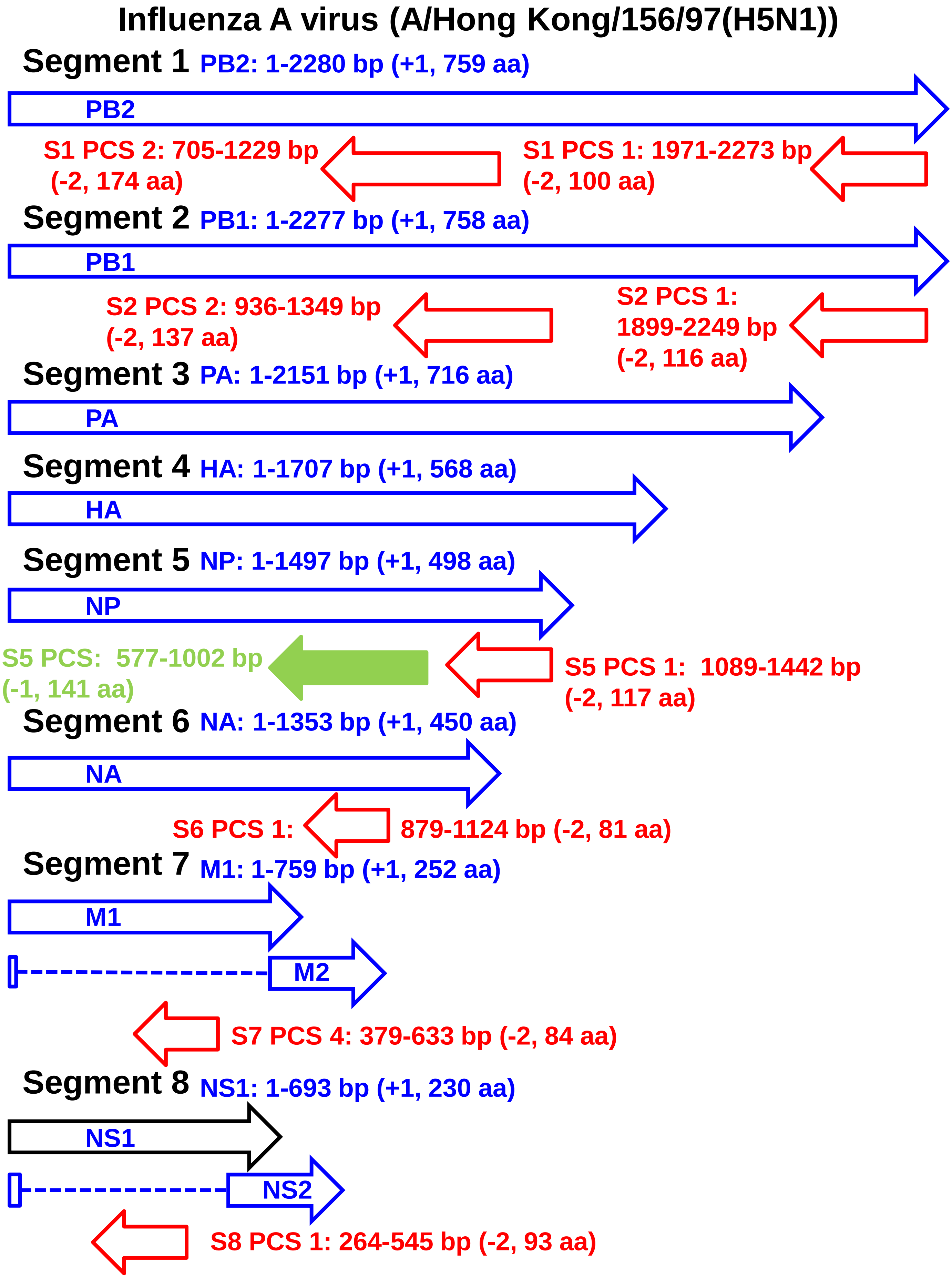

Influenza A virus (A/Hong Kong/156/97(H5N1))
Segment 1 PB2: 1-2280 bp (+1, 759 aa)
PB2
S1 PCS 2: 705-1229 bp (-2, 174 aa)
S1 PCS 1: 1971-2273 bp (-2, 100 aa)
Segment 2 PB1: 1-2277 bp (+1, 758 aa)
PB1
S2 PCS 2: 936-1349 bp (-2, 137 aa)
S2 PCS 1: 1899-2249 bp (-2, 116 aa)
Segment 3 PA: 1-2151 bp (+1, 716 aa)
PA
Segment 4 HA: 1-1707 bp (+1, 568 aa)
HA
Segment 5 NP: 1-1497 bp (+1, 498 aa)
NP
S5 PCS: 577-1002 bp (-1, 141 aa)
S5 PCS 1: 1089-1442 bp (-2, 117 aa)
Segment 6 NA: 1-1353 bp (+1, 450 aa)
NA
S6 PCS 1: 879-1124 bp (-2, 81 aa)
Segment 7 M1: 1-759 bp (+1, 252 aa)
M1
M2
S7 PCS 4: 379-633 bp (-2, 84 aa)
Segment 8 NS1: 1-693 bp (+1, 230 aa)
NS1
NS2
S8 PCS 1: 264-545 bp (-2, 93 aa)

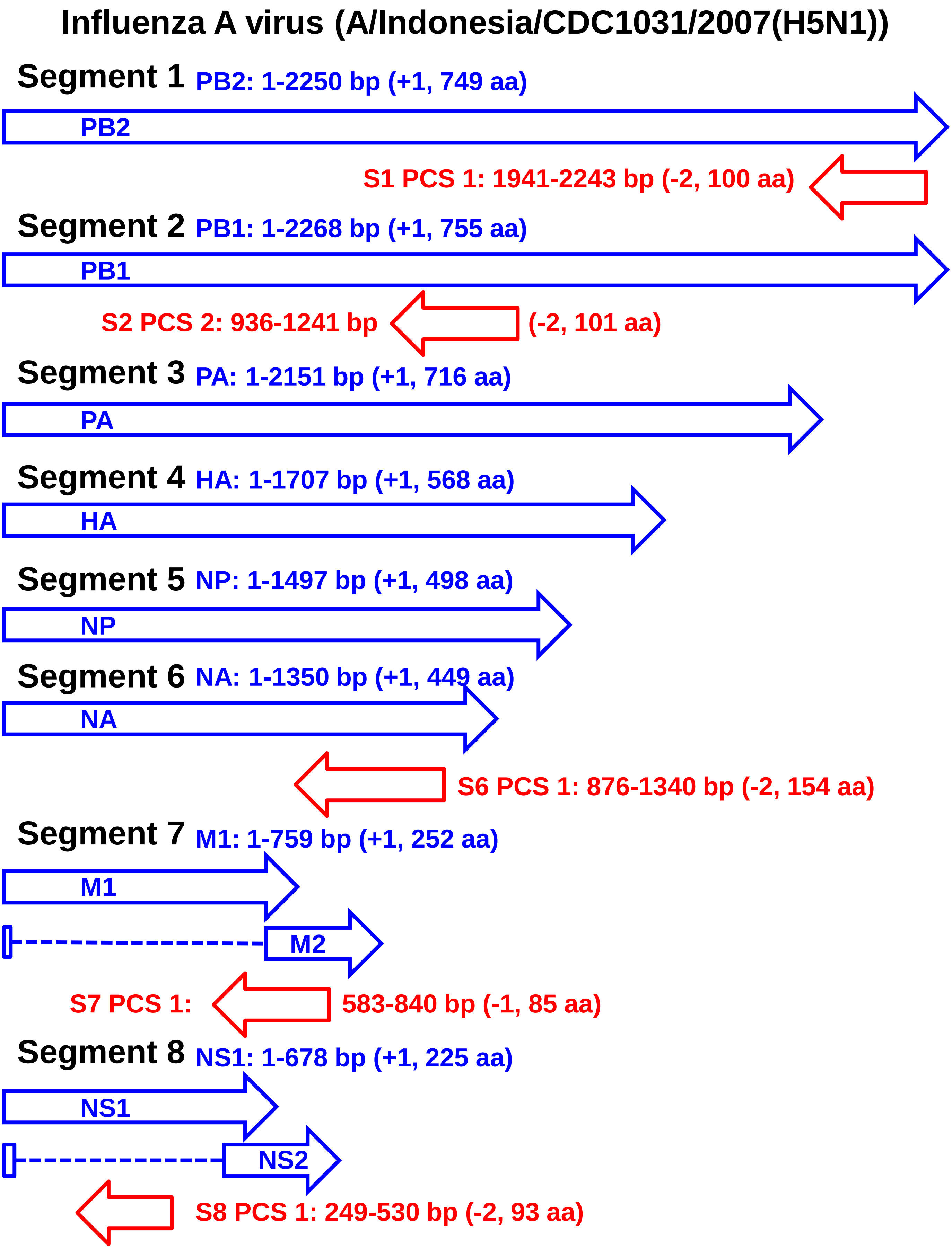
Influenza A virus (A/Indonesia/CDC1031/2007(H5N1))
Segment 1 PB2: 1-2250 bp (+1, 749 aa)
PB2
S1 PCS 1: 1941-2243 bp (-2, 100 aa)
Segment 2 PB1: 1-2268 bp (+1, 755 aa)
PB1
S2 PCS 2: 936-1241 bp (-2, 101 aa)
Segment 3 PA: 1-2151 bp (+1, 716 aa)
PA
Segment 4 HA: 1-1707 bp (+1, 568 aa)
HA
Segment 5 NP: 1-1497 bp (+1, 498 aa)
NP
Segment 6 NA: 1-1350 bp (+1, 449 aa)
NA
S6 PCS 1: 876-1340 bp (-2, 154 aa)
Segment 7 M1: 1-759 bp (+1, 252 aa)
M1
M2
S7 PCS 1: 583-840 bp (-1, 85 aa)
Segment 8 NS1: 1-678 bp (+1, 225 aa)
NS1
NS2
S8 PCS 1: 249-530 bp (-2, 93 aa)

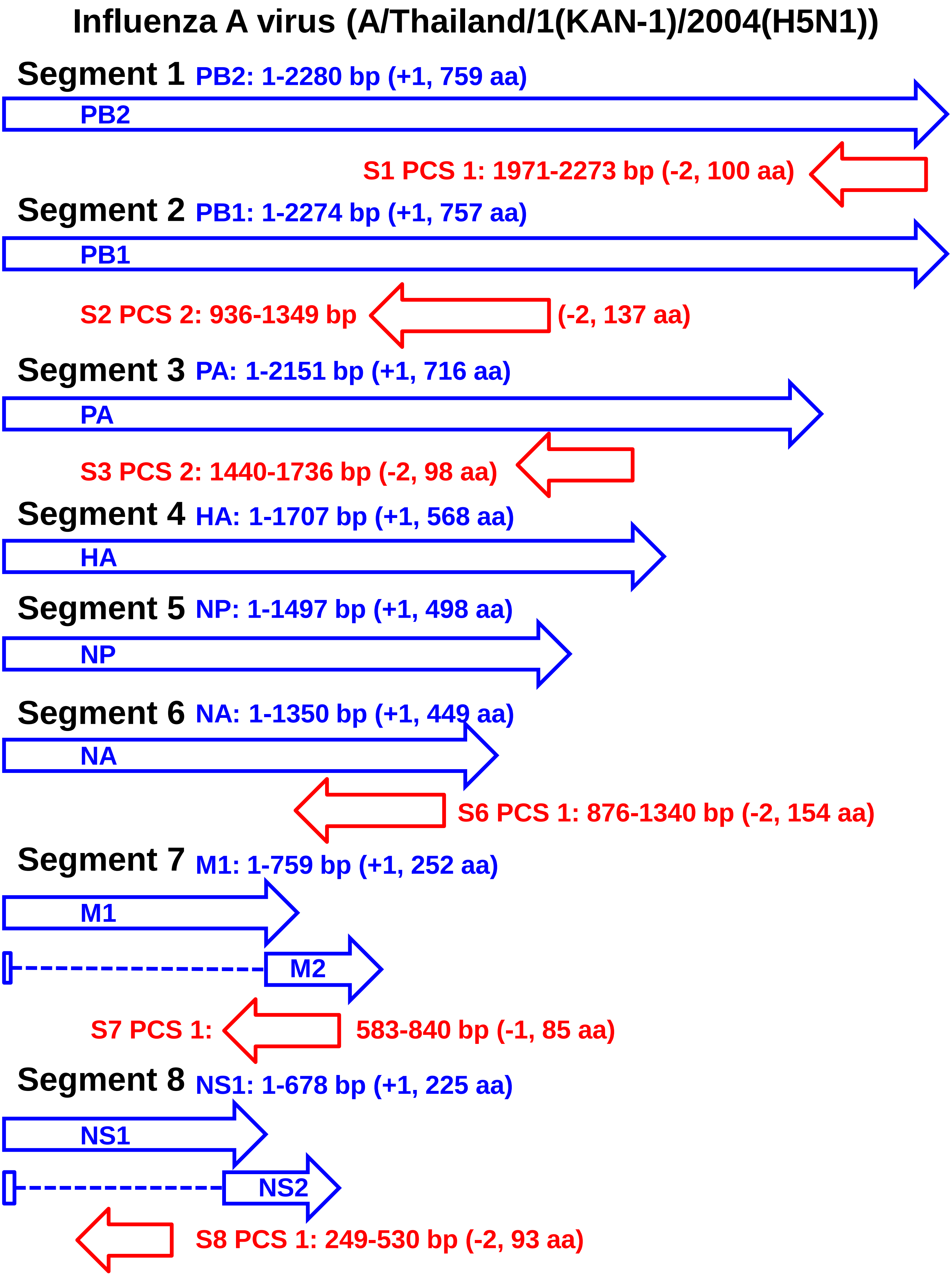
Influenza A virus (A/Thailand/1(KAN-1)/2004(H5N1))
Segment 1 PB2: 1-2280 bp (+1, 759 aa)
PB2
S1 PCS 1: 1971-2273 bp (-2, 100 aa)
Segment 2 PB1: 1-2274 bp (+1, 757 aa)
PB1
S2 PCS 2: 936-1349 bp
(-2, 137 aa)
Segment 3 PA: 1-2151 bp (+1, 716 aa)
PA
S3 PCS 2: 1440-1736 bp (-2, 98 aa)
Segment 4 HA: 1-1707 bp (+1, 568 aa)
HA
Segment 5 NP: 1-1497 bp (+1, 498 aa)
NP
Segment 6 NA: 1-1350 bp (+1, 449 aa)
NA
S6 PCS 1: 876-1340 bp (-2, 154 aa)
Segment 7 M1: 1-759 bp (+1, 252 aa)
M1
M2
S7 PCS 1:
583-840 bp (-1, 85 aa)
Segment 8 NS1: 1-678 bp (+1, 225 aa)
NS1
NS2
S8 PCS 1: 249-530 bp (-2, 93 aa)

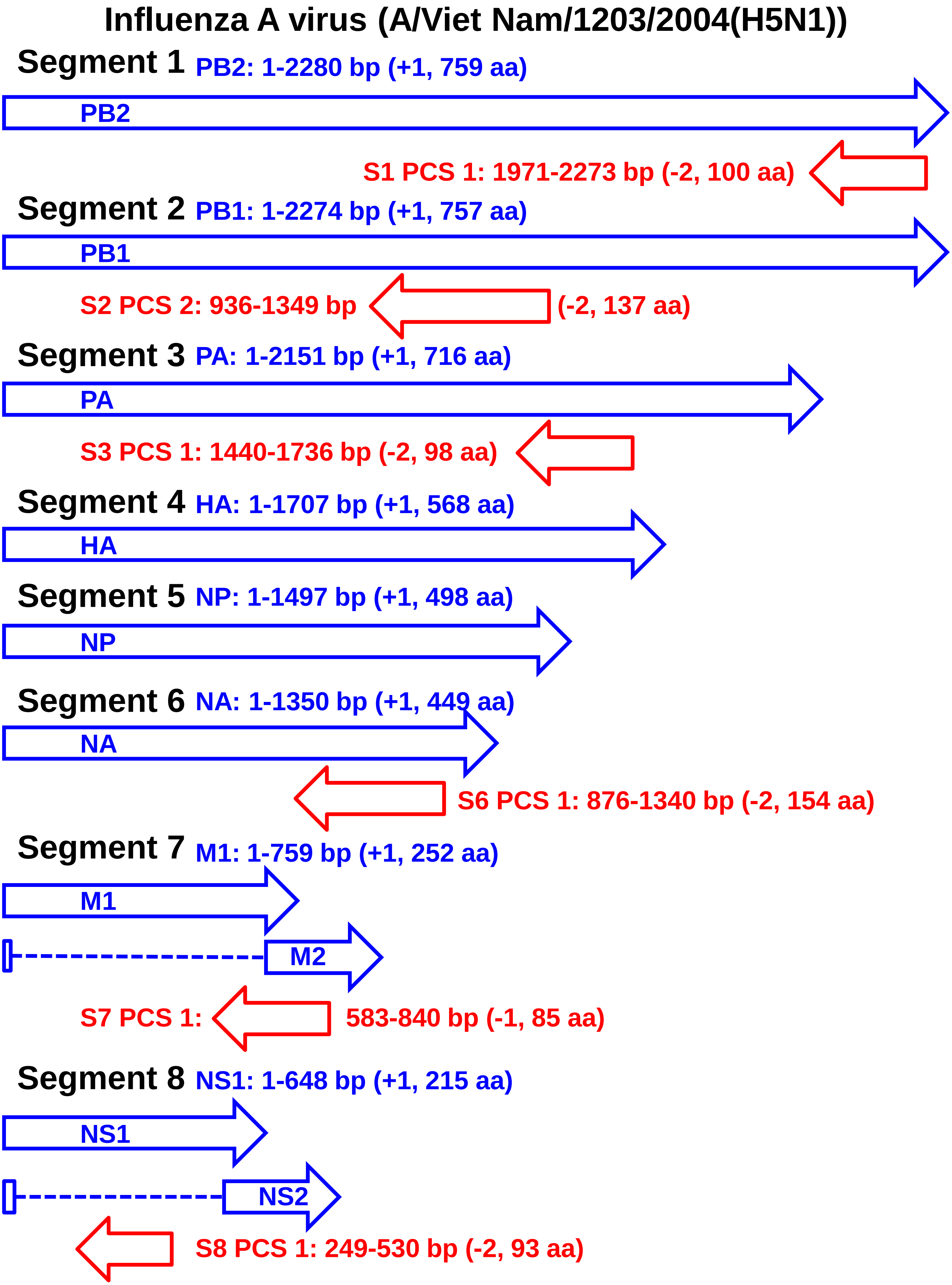

Influenza A virus (A/Viet Nam/1203/2004(H5N1))
Segment 1 PB2: 1-2280 bp (+1, 759 aa)
PB2
S1 PCS 1: 1971-2273 bp (-2, 100 aa)
Segment 2 PB1: 1-2274 bp (+1, 757 aa)
PB1
S2 PCS 2: 936-1349 bp (-2, 137 aa)
Segment 3 PA: 1-2151 bp (+1, 716 aa)
PA
S3 PCS 1: 1440-1736 bp (-2, 98 aa)
Segment 4 HA: 1-1707 bp (+1, 568 aa)
HA
Segment 5 NP: 1-1497 bp (+1, 498 aa)
NP
Segment 6 NA: 1-1350 bp (+1, 449 aa)
NA
S6 PCS 1: 876-1340 bp (-2, 154 aa)
Segment 7 M1: 1-759 bp (+1, 252 aa)
M1
M2
S7 PCS 1: 583-840 bp (-1, 85 aa)
Segment 8 NS1: 1-648 bp (+1, 215 aa)
NS1
NS2
S8 PCS 1: 249-530 bp (-2, 93 aa)

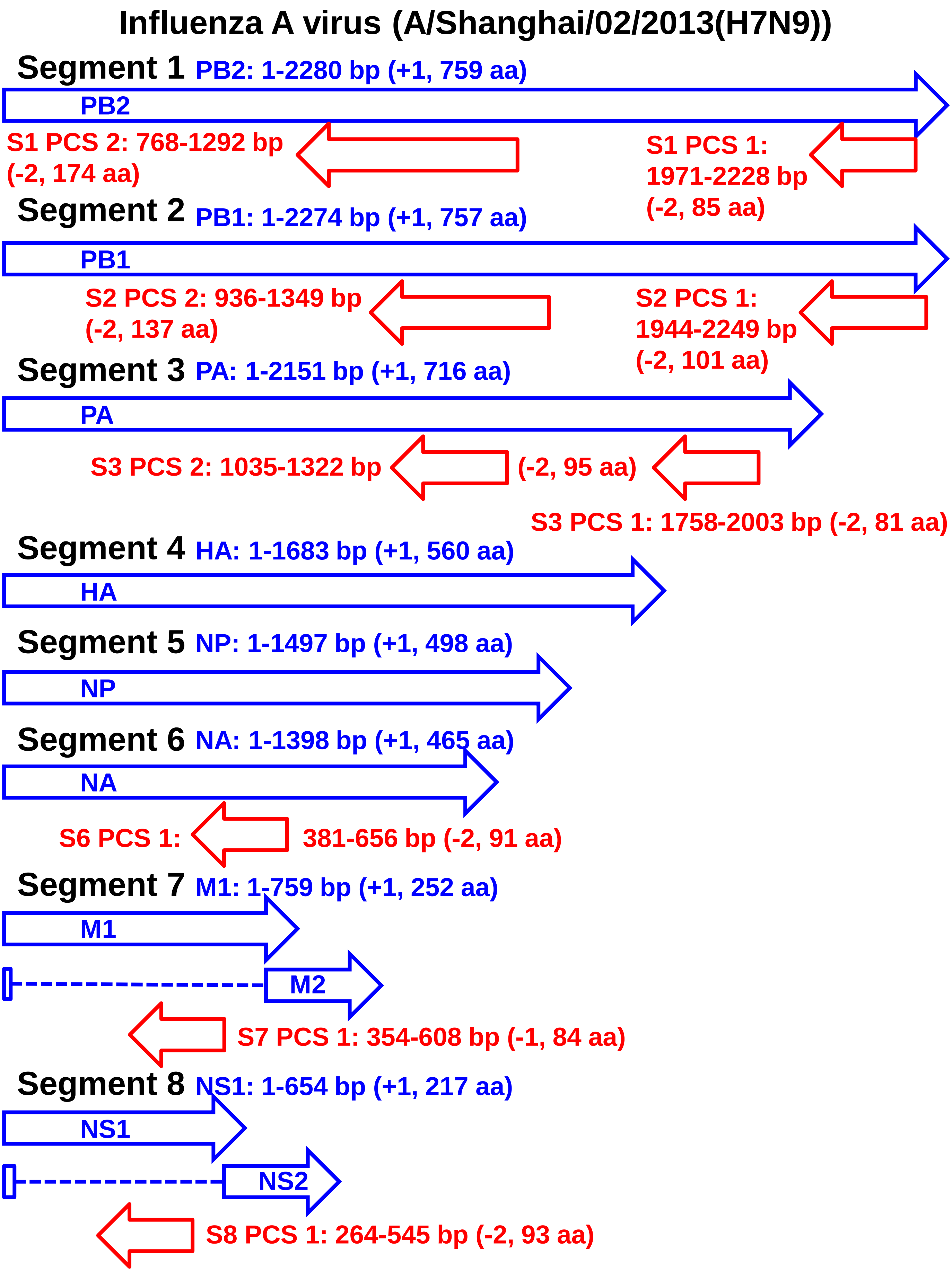
Influenza A virus (A/Shanghai/02/2013(H7N9))
Segment 1 PB2: 1-2280 bp (+1, 759 aa)
PB2
S1 PCS 2: 768-1292 bp (-2, 174 aa)
S1 PCS 1: 1971-2228 bp (-2, 85 aa)
Segment 2 PB1: 1-2274 bp (+1, 757 aa)
PB1
S2 PCS 2: 936-1349 bp (-2, 137 aa)
S2 PCS 1: 1944-2249 bp (-2, 101 aa)
Segment 3 PA: 1-2151 bp (+1, 716 aa)
PA
S3 PCS 2: 1035-1322 bp
(-2, 95 aa)
S3 PCS 1: 1758-2003 bp (-2, 81 aa)
Segment 4 HA: 1-1683 bp (+1, 560 aa)
HA
Segment 5 NP: 1-1497 bp (+1, 498 aa)
NP
Segment 6 NA: 1-1398 bp (+1, 465 aa)
NA
S6 PCS 1:
381-656 bp (-2, 91 aa)
Segment 7 M1: 1-759 bp (+1, 252 aa)
M1
M2
S7 PCS 1: 354-608 bp (-1, 84 aa)
Segment 8 NS1: 1-654 bp (+1, 217 aa)
NS1
NS2
S8 PCS 1: 264-545 bp (-2, 93 aa)

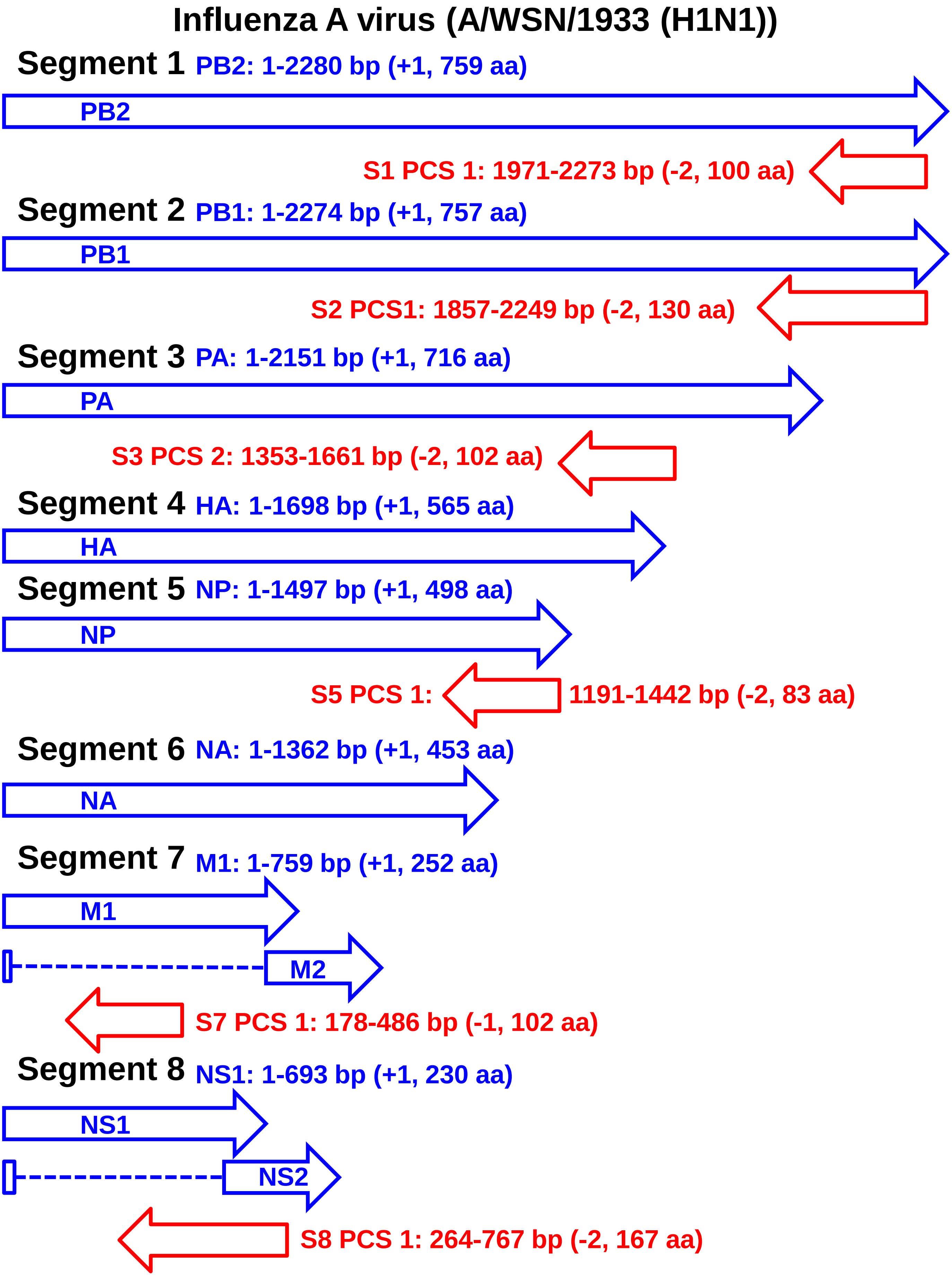
Influenza A virus (A/WSN/1933 (H1N1))
Segment 1 PB2: 1-2280 bp (+1, 759 aa)
PB2
S1 PCS 1: 1971-2273 bp (-2, 100 aa)
Segment 2 PB1: 1-2274 bp (+1, 757 aa)
PB1
S2 PCS1: 1857-2249 bp (-2, 130 aa)
Segment 3 PA: 1-2151 bp (+1, 716 aa)
PA
S3 PCS 2: 1353-1661 bp (-2, 102 aa)
Segment 4 HA: 1-1698 bp (+1, 565 aa)
HA
Segment 5 NP: 1-1497 bp (+1, 498 aa)
NP
S5 PCS 1:
1191-1442 bp (-2, 83 aa)
Segment 6 NA: 1-1362 bp (+1, 453 aa)
NA
Segment 7 M1: 1-759 bp (+1, 252 aa)
M1
M2
S7 PCS 1: 178-486 bp (-1, 102 aa)
Segment 8 NS1: 1-693 bp (+1, 230 aa)
NS1
NS2
S8 PCS 1: 264-767 bp (-2, 167 aa)

Influenza A virus (A/Puerto Rico/8/1934(H1N1))

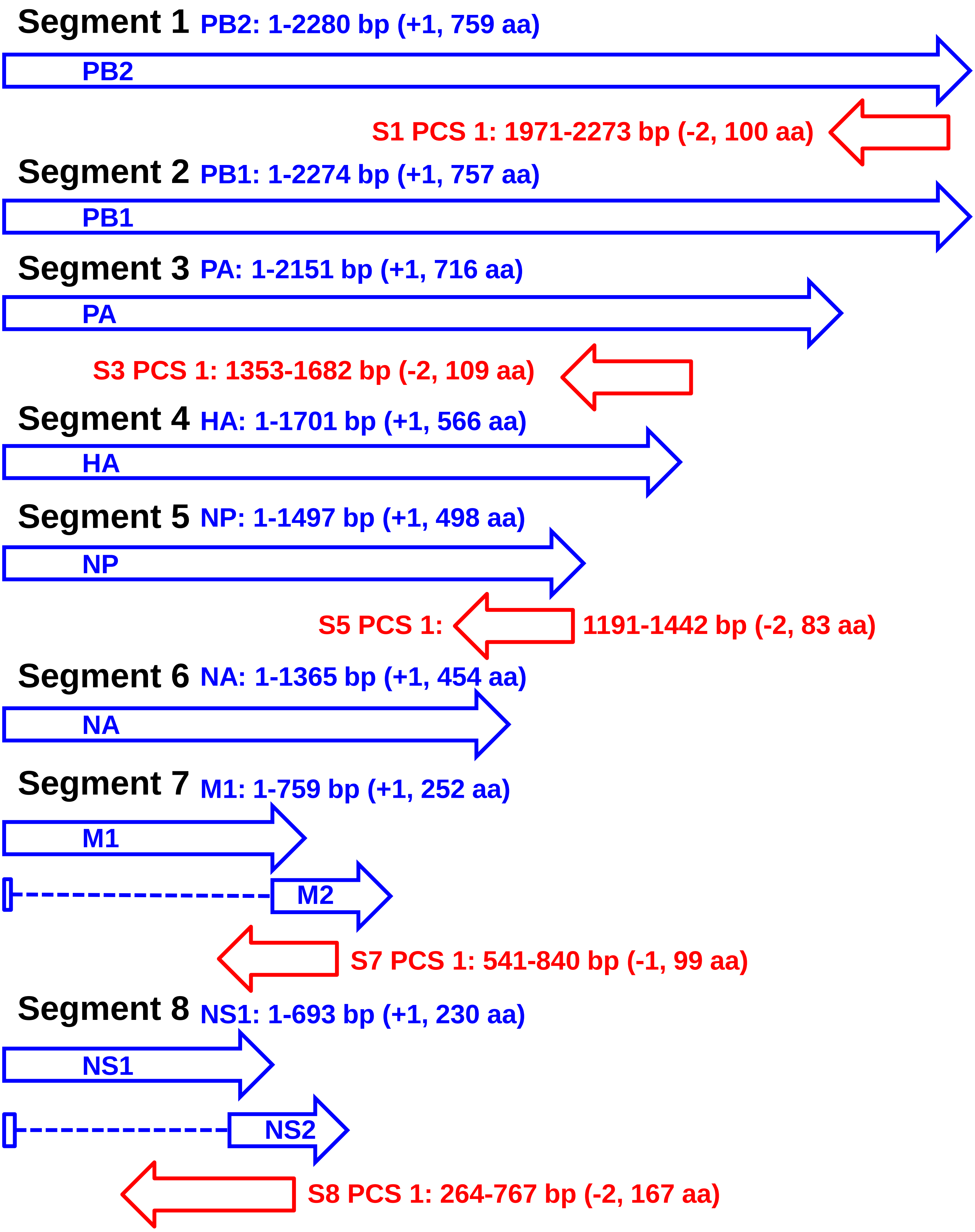

Supplement: S3 Fig — (PDF) [file pone.0146936.s003.pdf]
