# Supplementary material for: Uncovering the Potential Pan Proteomes Encoded by Genomic Strand RNAs of Influenza A Viruses
Source: PLoS One. 2016 Jan 13;11(1):e0146936. doi: 10.1371/journal.pone.0146936 (PMC4711952; doi:10.1371/journal.pone.0146936)
Supplement: S1 Table — (DOC) [file pone.0146936.s004.doc]

S2 Table. Number of IAV genomic RNA sequences used in this study.

|  | SEG1 | SEG2 | SEG3 | SEG4 | SEG5 | SEG6 | SEG7 | SEG8 | Sum |
| --- | --- | --- | --- | --- | --- | --- | --- | --- | --- |
| AIAV original SEQs | 13,336 | 13,279 | 13,290 | 19,418 | 12,945 | 15,588 | 14,014 | 13,580 | 115,450 |
| AIAV SEQs used for PCS prediction | 11,038 | 11,303 | 11,511 | 14,673 | 11,165 | 10,644 | 12,652 | 12,622 | 95,608 |
| AIAV PCSs | 23,222 | 14,681 | 21,613 | 6,223 | 19,297 | 9,617 | 19,876 | 9,704 | 124,233 |
| MIAV original SEQs | 3,521 | 3,889 | 3,865 | 18,024 | 3,924 | 6,976 | 6,426 | 4,132 | 50,757 |
| MIAV SEQs used for PCS prediction | 3,297 | 3,357 | 3,390 | 6,224 | 3,535 | 6,167 | 5,889 | 3,840 | 35,699 |
| MIAV PCSs | 6,403 | 6,857 | 7,925 | 2,843 | 4,625 | 6,739 | 8,824 | 3,639 | 47,855 |
| HIAV original SEQs | 13,445 | 13,388 | 13,302 | 39,827 | 13,966 | 27,176 | 20,521 | 14,103 | 155,728 |
| HIAV SEQs used for PCS prediction | 12,238 | 12,206 | 12,304 | 22,440 | 12,806 | 21,512 | 17,222 | 13,237 | 123,965 |
| HIAV PCSs | 18,511 | 22,267 | 17,711 | 16,762 | 8,515 | 26,713 | 29,999 | 13,503 | 153,981 |
| Total original SEQs | 30,602 | 30,556 | 30,457 | 77,269 | 30,835 | 49,740 | 40,961 | 31,815 | 322,235 |
| Total SEQs used for PCS prediction | 26,573 | 26,866 | 27,205 | 43,337 | 27,506 | 38,324 | 35,763 | 29,699 | 255,273 |
| Total PCSs | 48,136 | 43,805 | 47,249 | 25,828 | 32,437 | 43,069 | 58,699 | 26,846 | 326,069 |
