# Supplementary material for: Uncovering the Potential Pan Proteomes Encoded by Genomic Strand RNAs of Influenza A Viruses
Source: PLoS One. 2016 Jan 13;11(1):e0146936. doi: 10.1371/journal.pone.0146936 (PMC4711952; doi:10.1371/journal.pone.0146936)
Supplement: S2 Table — (DOC) [file pone.0146936.s005.doc]

**S3 Table. Number of sequences and sub-clusters of the 20 PCSGs.**

| PCSG | Seq. No | Cluster No | S/C | PCSG | Seq. No | Cluster No | S/C |
| --- | --- | --- | --- | --- | --- | --- | --- |
| S1 PCS G1 | 26527 | 149 | 178.0 | S5 PCS G1 | 19571 | 131 | 149.4 |
| S1 PCS G2 | 15207 | 163 | 93.3 | S5 PCS G2 | 4375 | 52 | 84.1 |
| S2 PCS G1 | 15436 | 60 | 257.3 | S5 PCS G3 | 7169 | 604 | 11.9 |
| S2 PCS G2 | 27254 | 225 | 121.1 | S6 PCS G1 | 7319 | 94 | 77.9 |
| S3 PCS G1 | 5594 | 109 | 51.3 | S6 PCS G2 | 9841 | 155 | 63.5 |
| S3 PCS G2 | 18216 | 204 | 89.3 | S7 PCS G1 | 9580 | 447 | 21.4 |
| S3 PCS G3 | 9554 | 319 | 29.9 | S7 PCS G2 | 14945 | 32 | 467.0 |
| S4 PCS G1 | 10130 | 200 | 50.7 | S7 PCS G3 | 10398 | 152 | 68.4 |
| S4 PCS G2 | 6098 | 27 | 225.9 | S7 PCS G4 | 23751 | 93 | 255.4 |
| S4 PCS G3 | 2632 | 85 | 31.0 | S8 PCS G1 | 20389 | 413 | 49.4 |

S1, S2, ... S8 in the first column represent Segment 1 ... Segment 8 of influenza a virus genomes.

Seq. No: Number of predicted protein sequences in the PCSG.

Cluster No: Number of sequences cluster.

S/C: ratio of sequence to cluster.
